# *In silico* Analysis of Peptide-Based Biomarkers for the Diagnosis and Prevention of Latent Tuberculosis Infection

**DOI:** 10.3389/fmicb.2022.947852

**Published:** 2022-06-28

**Authors:** Peng Cheng, Liang Wang, Wenping Gong

**Affiliations:** ^1^Tuberculosis Prevention and Control Key Laboratory/Beijing Key Laboratory of New Techniques of Tuberculosis Diagnosis and Treatment, Senior Department of Tuberculosis, The 8^th^ Medical Center of PLA General Hospital, Beijing, China; ^2^Hebei North University, Zhangjiakou, China; ^3^Department of Geriatrics, The 8^th^ Medical Center of PLA General Hospital, Beijing, China

**Keywords:** tuberculosis (TB), latent tuberculosis infection (LTBI), biomarker, vaccine, diagnosis

## Abstract

**Background:**

Latent tuberculosis infection (LTBI) is the primary source of active tuberculosis (ATB), but there are no specific methods for diagnosing and preventing LTBI.

**Methods:**

Dominant T and B cell epitopes predicted from five antigens related to LTBI and *Mycobacterium tuberculosis* region of difference (LTBI-RD) were used to construct a novel polypeptide molecule (PPM). Then, the physicochemical properties, secondary structure, tertiary structure of the PPM, and its binding ability to toll-like receptor 2 (TLR2) and TLR4 were analyzed by bioinformatics tools. Finally, immune stimulation and expression optimization of the PPM were carried out.

**Results:**

Four helper T lymphocytes (HTL) epitopes, five cytotoxic T lymphocytes (CTL) epitopes, and three B cell epitopes were predicted and screened from five LTBI-RD related antigens. These epitopes were connected in series with linkers and adjuvants to construct a novel PPM termed C543P. The results indicated that antigenicity and immunogenicity scores of the C543P candidate were 0.936399 and 1.36469, respectively. The structural analysis results showed that the C543P candidate had good stability. Its secondary structure contained 43.6% α-helix, the Z-score after tertiary structure optimization was −7.9, and the Ramachandran diagram showed that 88.77% amino acid residues of the C543P candidate were in the allowable region. Furthermore, the C543P candidate showed an excellent affinity to TLR2 (−1091.7kcal/mol) and TLR4 (−1102.7kcal/mol). In addition, we also analyzed the immunological characteristics of the C543P candidate. Immune stimulation prediction showed that the C543P candidate could effectively activate T and B lymphocytes and produce high levels of Th1 cytokines such as IFN-γ and IL-2.

**Conclusion:**

We constructed a novel PPM with acceptable antigenicity, immunogenicity, stability, and ability to induce robust immune responses. This study provides a new diagnostic biomarker or peptides-based vaccine for LTBI diagnosis and prevention.

## Introduction

Tuberculosis (TB), a respiratory infectious disease caused by *Mycobacterium tuberculosis* (MTB) infection, is the second leading cause of death from a single infectious agent after coronavirus disease 2019 (COVID-19). The Global Tuberculosis Report released by the World Health Organization (WHO) pointed out that there were 10 million new TB cases and 1.3 million deaths globally in 2020 (WHO, 2021). TB prevention and control are facing severe challenges, such as latent TB infection (LTBI), multidrug-resistant TB (MDR-TB), rifampicin-resistant TB (RR-TB), and co-infection with SARS-CoV-2 or human immunodeficiency virus (HIV) (WHO, 2021). Previous studies have shown that more than 85% of ATB patients originate from LTBI (Gong and Wu, 2021). LTBI is a subclinical mycobacterial infection defined on the basis of cellular immune response to mycobacterial antigens without clinical manifestations in patients with ATB (Carranza et al., 2020). Previous studies reported that about 23% of the global population had been infected with MTB, but only 10% developed ATB, and nearly 90% of these people are LTBI (Houben and Dodd, 2016; Gong et al., 2018). However, there are no specific diagnostic methods for LTBI and ATB differential diagnosis. Only two methods were approved by the WHO for auxiliary diagnosis of LTBI, including tuberculin skin test (TST) and interferon-gamma (IFN-γ) release assay (IGRA) (Zellweger et al., 2020; Mosavari et al., 2021).

Traditional TST uses purified protein derivative (PPD) as an antigen stimulant. Although this method has the advantages of low price, simplicity, and no need for special equipment, it can not eliminate the interference caused by Bacillus Calmette–Guérin (BCG) vaccination. Therefore, some new TST and IGRAs diagnosis methods have been developed in recent years, including Diaskintest, C-Tb skin test, EC-Test, T-cell spot of TB assay (T-SPOT.TB), QuantiFERON-TB Gold In-Tube (QFT-GIT), QuantiFERON-TB Gold-Plus (QFT-Plus), LIAISON QFT-Plus, and LIOFeron TB/LTBI (Gong and Wu, 2021). These new technologies coincidentally replace the PPD antigen used in the traditional TST with early secreted antigenic target 6 (EAST-6) and culture filtrate protein 10 (CFP-10) antigens. EAST-6 and CFP10 are encoded by Rv3874 and Rv3875 genes, located in the MTB Region of Difference 1 (RD1) (Tang et al., 2014). Both antigens can eliminate BCG vaccination’s interference in LTBI diagnosis and reduce the false-positive diagnosis rate. Unfortunately, EAST-6 and CFP10 antigens are not expressed in the latent period of MTB, so it is still not possible to distinguish the LTBI population from ATB patients.

Our previous study found that antigens related to LTBI and RD (LTBI-RD) were promising candidates for LTBI differential diagnosis (Gong and Wu, 2021). Among these 21 LTBI-RD related antigens, five antigens (Rv1737c, Rv2659c, Rv2660c, Rv1981c, and Rv3879c) have been widely reported to be candidate biomarkers for discriminating diagnosis of LTBI. It has been reported that, compared with patients with ATB, the Rv1737c antigen located in the DosR region of MTB could induce significantly higher levels of IFN-γ and TNF-α and promote the expression of CD4^+^ T cells and CD8^+^ T cells with CD45RO^+^CD27^+^ phenotype in peripheral blood in individuals with LTBI (Arroyo et al., 2016, 2018). Furthermore, Bai et al. (2015) compared the ability of Rv2659c to stimulate the release of IFN-γ in TB patients, non-TB patients, and LTBI individuals, and they found that the IFN-γ level in LTBI individuals was significantly higher than that in ATB patients. In addition, a study in China using Rv2659c to screen LTBI in recruits also proved that Rv2659c can identify individuals with LTBI with an accuracy rate of 70%, so this antigen may be one of the candidate antigens for discriminating LTBI (Bai et al., 2017). He and colleagues used recombinant protein Rv2660c to evaluate its diagnostic value by enzyme-linked immunosorbent assay (ELISA) (He et al., 2015). The results showed that the number of plaques formed in the LTBI individuals stimulated by the Rv2660c antigen was significantly higher than in ATB patients and healthy populations (He et al., 2015). Furthermore, cytokines such as IL-2, IL-10, IFN-γ, and MIP-1a induced by Rv2660c antigen in LTBI individuals were significantly higher than those in TB patients. These results indicated that Rv2660 could induce more robust immune responses in individuals with LTBI (He et al., 2015).

Based on the evidence mentioned above, in this study, we predicted and screened helper T lymphocytes (HTL), cytotoxic T lymphocytes (CTL), and B cell epitopes from five antigens related to LTBI-RD to construct a polypeptide molecule (PPM) for diagnosing and preventing LTBI. We predicted the antigenicity, immunity, sensitization, physicochemical properties, and secondary structure of the PPM. We also performed the tertiary structure modeling and model validation of the PPM and docked the model with the human toll-like receptor (TLR) to understand its binding affinity. Subsequently, the thermodynamic stability of the PPM was analyzed, and the immune response process of immune cells *in vivo* after antigen injection was predicted by the C-ImmSim server. Finally, electronic cloning and codon optimization were carried out to better express this PPM. This study aimed to design a PPM covering HTL, CTL and B cell epitopes simultaneously, which could provide a novel candidate target for the diagnosis and prevention of LTBI.

## Materials and Methods

### Antigens Selection

In our previous study, we screened 21 most potential LTBI-RD related antigens from 133 RD antigens and 124 latency-associated antigens, including Rv1736c, Rv1737c, Rv2031c, Rv2626c, Rv2653c, Rv2654c, Rv2656c, Rv2657c, Rv2658c, Rv2659c, Rv2660c, Rv1511, Rv1978, Rv1980c, Rv1981c, Rv3872, Rv3873, Rv3878, Rv3879c, Rv3425, and Rv3429 (Gong and Wu, 2021). Through extensive literature reading, we found that five antigens have great potential in identifying LTBI, including Rv1737c (NarK2), Rv2659c, Rv2660c, Rv1981c (nrdf1), and Rv3879c (espk) (Table 1). The amino acid sequences of these candidate antigens were downloaded through the Mycobrowser server^1^ (Kapopoulou et al., 2011).

**TABLE 1:** Characteristics of five selected LTBI-RD-associated antigens.

Antigen name	Gene	Product	Length (aa)	Functions	References
Rv1737c	*narK2*	Possible nitrate/nitrite transporter NarK2	395	(1) Higher TNF-α^+^ CD4^+^ T cells and IFN-γ^+^ TNF-α^+^ CD4^+^ T cells in LTBI vs. PTB. (2) Higher IFN-γ^+^ TNF-α^+^ CD8^+^ T cells in LTBI vs. HC	Arroyo et al., 2016; Chegou et al., 2012
Rv2659c	*Rv2659c*	Probable PhiRv2 prophage integrase	375	higher IFN-γ producing T cells in LTBI vs. aTB & HC	Bai, 2015
Rv2660c	*Rv2660c*	Hypothetical protein	75	(1) Induces stronger immune response in LTBI vs aTB (2) A component of vaccine H56:IC31 and will affect the diagnosis of Rv2660c once the vaccine is available	He et al., 2015; Luabeya et al., 2015
Rv1981c	*nrdF1*	Ribonucleoside-diphosphate reductase (beta chain) NrdF1	322	ELISPOT of Rv1981c achieved sensitivities of 60% in aTB and specificities of 90% in BCG-vaccinated HC.	Chen et al., 2009
Rv3879c	*espK*	ESX-1 secretion-associated protein EspK	729	The immunodominance of Rv3879c is higher than that of Rv3878 and Rv3873 in aTB and LTBI subjects.	Hinks et al., 2009

### Prediction of HTL Epitopes

Immune Epitope Database (IEDB) is a free resource funded by the National Institute of Allergy and Infectious Diseases (NIAID). This database documents experimental data on antibodies and T-cell epitopes studied in humans, non-human primates, and other animal species in infectious diseases, allergy, autoimmunity, and transplantation. This study used IEDB’s major histocompatibility complex (MHC) II server^2^ to predict HTL epitopes (Zhang et al., 2008). Parameter settings are as follows: Prediction Method = IEDB recommended 2.22, MHC source species = Human, MHC allele(s) = total human leukocyte antigen (HLA) reference set (HLA-DR, HLA-DP, HLA-DQ), epitope length = 15. Potential HTL epitopes were selected using the percentile rank method. The percentile rank is obtained by comparing the peptide score with five million 15-mers in the SWISSPROT database. The lower the percentile rank score represents the higher binding of the epitope to MHC II. We choose the epitopes whose percentile rank is less than 0.5 for further exploration in this study. Then, the VaxiJen v2.0^3^ was used to predict the antigenicity of the epitopes, and the threshold was 0.5 (Doytchinova and Flower, 2007). VaxJen v2.0 lists selected targets by auto-cross-covariance (ACC) transformation and gives predicted probabilities and protective antigen or non-protective claims. Next, the IFN-gamma epitope server^4^ was used to predict the IFN-γ inducibility of epitopes (Dhanda et al., 2013). The prediction method was Motif and SVM hybrid, and the model was IFN-γ versus Non-IFN-gamma. Then, epitopes with positive predictions were selected. Finally, the epitopes that passed the above screening were determined as the immunodominant HTL epitopes for constructing the PPM.

### Prediction of CTL Epitopes

Cytotoxic T lymphocytes plays a vital role in controlling the occurrence and development of TB. Therefore, we predicted the CTL epitopes of five LTBI-RD related antigens using the IEDB MHC I server.^5^ This server predicts epitopes that bind to MHC I molecules and uses Artificial Neural Network (ANN) 4.0 for 36 HLA-A alleles, 34 HLA-B alleles, and 10 HLA-C alleles (Kim et al., 2012). Parameter settings: Prediction Method = IEDB recommended 2020.09 (NetMHCpan EL4.1), MHC source species = Human, MHC allele(s) = HLA allele reference set, and epitope length(s) = All lengths. We selected CTL epitopes with a percentile rank of <0.5 for subsequent analysis. Subsequently, the Class I Immunogenicity server^6^ was selected to analyze the immunogenicity of these epitopes. The CTL epitopes with a percentile rank of <0.5 and immune scores >0 were selected for further analysis. Finally, the VaxiJen v2.0 server was used to predict the antigenicity of the above epitopes, and the selection threshold was set at 0.5 (Doytchinova and Flower, 2007). The epitopes that passed the above screening were determined as the immunodominant CTL epitopes for constructing PPM.

### Prediction of Linear B-cell Epitopes

Recent studies have shown that B cells play an essential role in antigen presentation and killing MTB, but they are often overlooked. Herein, the ABCpred server^7^ was used to predict linear B-cell epitopes of the five LTBI-RD antigens. The ABCpred server uses artificial neural networks to predict linear B-cell epitope regions in antigen sequences and is widely used in candidate diagnostic antigen and vaccine research (Saha and Raghava, 2006). The server predicted epitopes with an accuracy of 65.93%. Parameter setting: epitope length = 20, the screening threshold = 0.51. Thresholds range from +0.1 to +1.0, and an increased threshold will enhance the specificity but decrease the sensitivity. Finally, the epitopes that passed the above screening were determined to be the immunodominant B cell epitopes for constructing the PPM.

### Construction of PPM for LTBI Diagnosis and Prevention

We linked the predicted and screened immunodominant HTL, CTL, and B cell epitopes by using linkers to construct a novel PPM for LTBI diagnosis and prevention. Adjuvant and pan HLA DR-binding epitope (PADRE) were added at the amino terminal of PPM. Besides, adjuvant and 6 × His tag (HHHHHH) were added at the carboxyl-terminal of PPM. The adjuvants used in the PPM included TLR4 receptor agonist CTB (AIE88420.1) (Meza et al., 2017) and TLR2 receptor agonist Pam2Cys (Jackson et al., 2004), and the linkers chosen in this study included GGPPG, AAY, and KK.

### Immunogenicity, Antigenicity, Allergenicity, and Toxicity Prediction of PPM

The immunogenicity of the PPM was predicted by using the IEDB Immunogenicity server (see footnote 6). The antigenicity of PPM was predicted by using VaxiJen v2.0 (see footnote 3) and ANTIGENpro server,^8^ respectively. The parameters in both servers were set as default values. The ANTIGENpro server is based on cross-validation experiments with 76% accuracy on the combined dataset (Magnan et al., 2010). Identifying allergens is of great significance to developing diagnostic candidates and vaccines. Herein, two servers, AllerTOP v.2.0 server^9^ and Allergen FP v.1.0 server,^10^ were used to predict the sensitization of the PPM. AllerTOP v.2.0 develops the k nearest neighbors (kNN), ACC transformation, and amino acid E-descriptors machine learning techniques for classifying allergens by exploring the physiochemical properties of proteins. The accuracy of the AllerTOP v.2.0 was stated as 85.3% at fivefold cross-validation (Dimitrov et al., 2014a). AllerFP v.1.0 uses an alignment-free, descriptor-based fingerprint method to identify known allergens and non-allergens. AllerFP v.1.0 correctly recognized 88% of them with a Matthews correlation coefficient of 0.75945 (Dimitrov et al., 2014b). Finally, the ToxinPred server (http://crdd.osdd.net/raghava/toxinpred/) was used to predict the toxicity of the PPM.

### Physiochemical Properties and Solubility Prediction

The physicochemical properties of the PPM were predicted using the Expasy Protparam server.^11^ This server can indicate molecular protein weight, theoretical isoelectric point (pI), amino acid composition, *in vitro* and *in vivo* half-life, instability, aliphatic index, and grand average of hydrophilicity (GRAVY) (Gasteiger, 2005). Then, the Protein-Sol server^12^ was used to predict the solubility of the PPM. The population average for the experimental dataset (PopAvrSol) is 0.45. Therefore, if the prediction result is >0.45, the candidate protein shows a higher solubility than the average soluble *Escherichia coli* (*E. coli*) protein from the experimental solubility dataset (Hebditch et al., 2017).

### Secondary Structure and Three-Dimensional Structure Prediction and Refinement

The PSIPRED^13^ and Prabi servers^14^ were used to predict the secondary structure of the PPM. PSIPRED is a secondary structure generation tool that provides an excellent prediction of transmembrane topology, transmembrane helix, folds, domain recognition, etc. (McGuffin et al., 2000). Prabi severs use GOR4 to predict the secondary structure of peptide molecules, and the average accuracy of this method is 64.4% (Garnier et al., 1996). In addition, the I-TASSER server^15^ was used to predict the three-dimensional (3D) spatial structure of polypeptide molecules. The I-TASSER server is a platform for computerized protein structure and function prediction based on sequence-to-structure-to-function paradigms (Yang et al., 2015). At the same time, the I-TASSER server generates 3D atomic models through several thread alignments and iterative structural assembly simulations, and the estimated accuracy of the predictions is based on the modeled confidence score (C-score) of the modeling. In general, the value of the C-score is between −5 and 2. Therefore, the higher the value, the higher the accuracy (Roy et al., 2010). Then, the 3D model of the PPM model was optimized using the GalaxyRefine web server^16^ to improve the quality of its partial or complete structure (Heo et al., 2013).

### Validation of 3D Structure

The ProSA-web server^17^ and the ERRAT-web server^18^ were used to evaluate the possible errors in the 3D structure of the PPM. The ProSA-web server accurately enters the frame by estimating the total quality score exact input structure and displays it in the form of a Z score. When the Z score exceeds the properties of the native protein, it indicates that the structure contains errors (Wiederstein and Sippl, 2007). The ERRAT-web server predicts the high-resolution crystallography structure of the PPM from non-bonded atom–atom interactions (Colovos and Yeates, 1993). Then, the SWISS-MODEL server^19^ was used to draw a Ramachandran diagram for the PPM (Waterhouse et al., 2018). The Ramachandran diagram shows the favorable region of the dihedral angle of the main chain relative to the amino acid residues in the protein structure (Waterhouse et al., 2018). The structure evaluation page shows the most relevant scores provided by MolProbity and helps us quickly identify the location of low-quality residues in its model or structure (Waterhouse et al., 2018).

### Prediction of Discontinuous B-cell Epitopes

Most B cell epitopes are discontinuous, and the constructed proteins are folded in different ways to form discontinuous B cell epitopes. So we predict their discontinuous epitopes by using the ElliPro server^20^ following a previous study (Ponomarenko et al., 2008).

### Molecular Docking With Immune Receptors

Molecular docking is the most basic and promising method to describe the interaction and binding affinity between peptides and human TLR2 and TLR4. We obtained PDB files for TLR2 (PDB ID: 6NIG) and TLR4 (PDB ID: 4G8A) from the NCBI Molecular Modeling Database (MMDB).^21^ Firstly, the ClusPro 2.0 online server^22^ was used for ligand-receptor docking analysis (Kozakov et al., 2017). Secondly, the protein-protein docking complexes were refined and re-scored using the PatchDock server^23^ and FireDock server^24^ (Schneidman-Duhovny et al., 2005; Andrusier et al., 2007; Mashiach et al., 2008). Finally, the HawDOCK server^25^ was used to measure the Molecular Mechanics/Generalized Born Surface Area (MM-GBSA) score. The lower the prediction score, the better the prediction result (Weng et al., 2019).

### Molecular Dynamics Simulation and Immune Simulation

Molecular dynamics simulation of the PPM was explored using the iMODS web-server,^26^ through which the flexibility of the PPM can be defined and calculated (Lopéz-Blanco et al., 2011). Then, the C-ImmSim server^27^ was used to predict the PPM’s ability to induce immune cells to produce specific antibodies and various cytokines. In addition, this server can also estimate the immune response of B lymphocytes and T lymphocytes (including Th1 and Th2 lymphocytes) (Rapin et al., 2010). The parameters are set as follows: random seed, simulation volume, and simulation steps were selected default values; HLA selection: A0101, A0201, B0702, B0801, DRB10101, and DRB1501. Finally, one injection of the PPM was conducted in the human host.

### Codon Optimization and *in silico* Cloning

Codon optimization can improve the expression of recombinant proteins. We used the Java Codon Adaptation Tool (JCAT) server^28^ to provide a feasible method for constructing a multi-epitopes-based recombinant plasmid. This server offers a codon-optimized version of the interest DNA sequence based on a chosen organism (Grote et al., 2005). The expression vector *E. coli* (K12) was selected. The output includes two parameters: codon adaptation index (CAI) and GC content percentage. In general, the ideal value of CAI is 1, and the GC content is 30–70%. The PPM sequence was inserted between *EcoR I* and *Xbn I* restriction sites of the pET30a (+) plasmid. SnapGene software was used to finalize *in silico* cloning.

## Results

### Selection of Immunodominant Epitopes and Construction of the PPM

**FIGURE 1:**
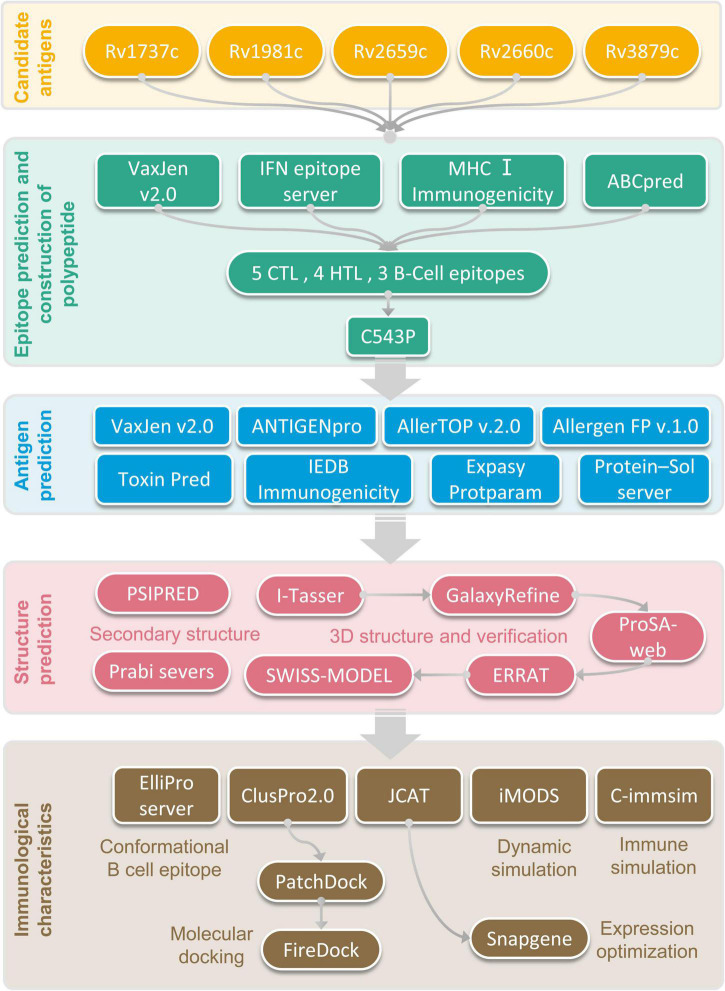
Flow chart of epitope prediction, screening, construction, and PPM analysis.

The flow chart of prediction, screening, construction, and physicochemical properties of immunodominant epitopes was shown in Figure 1. One hundred and fifty-seven HTL epitopes (Supplementary Table 1) and 1184 CTL epitopes (Supplementary Table 2) with a percentile rank score of <0.5 were identified by using IEDB MHC II and MHC I web servers, respectively. Then, 18 epitopes with antigenicity score >0.7 and positive IFN-γ inducibility were screened from 157 HTL epitopes. In addition, 60 epitopes with immunogenicity > 0 and antigenicity > 1 were screened from 1184 CTL epitopes (Supplementary Table 3). Finally, we selected 8 epitopes with a truncated binding score > 0.9 from 154 B cell epitopes using the ABCpred server (Supplementary Table 4).

**FIGURE 2:**
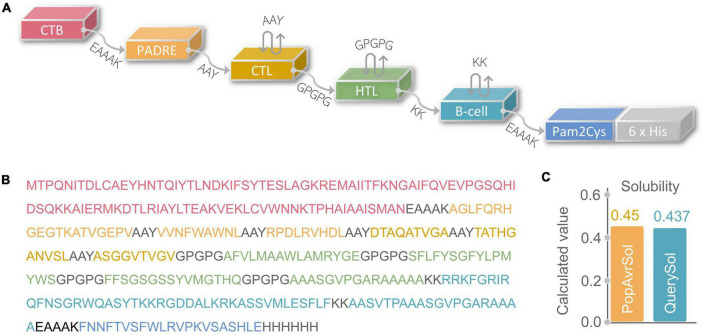
**(A)** Schematic diagram of C543P polypeptide molecular construction. **(B)** Amino acid sequence. **(C)** Solubility analysis predicted by the Protein-Sol server.

The potential immunodominant epitopes were selected following the below principles: (1) HTL epitopes with antigenicity > 0.7 and the highest IFN prediction score in each antigen were selected; (2) CTL epitopes with the highest immunogenicity score in each antigen were selected; (3) B cell epitopes with the highest ABCpred prediction score (>0.9) were selected. Thus, four HTL, five CTL, and three B cell epitopes were finally selected to construct a new PPM (Table 2). The GGPPG, AAY, or KK linker was chosen to connect HTL, CTL, or B cell epitopes, respectively. Adjuvant or agonist CTB (MTPQNITDLCAEYHNTQIYTLNDKIFSYTESLAGKREMAII TFKNGAIFQVEVPGSQHIDSQKKAIERMKDTLRIAYLTEAKV EKLCVWNNKTPHAIAAISMAN), PADRE (AGLFQRHGEGT KATVGEPV), and Pam2Cys (FNNFTVSFWLRVPKVSASHLE) were linked with EAAAK linkers. The above-constructed PPM was named C543P, and its construction pattern and amino acid sequence were shown in Figures 2A,B, respectively.

**TABLE 2:** Overall the HTL, CTL, and B-cell epitopes selected to construct the C543P candidate.

Protein	Peptide sequence	Length	Alleles	Percentile rank	Antigenicity score	IFN-γ score	Immunogenicity score	ABC pred score
**HTL epitopes**								
Rv1981c	SFLFYSGFYLPMYWS	15	HLA-DPA1*01:03/DPB1*04:01	0.01	0.8327	1	–	–
	FFSGSGSSYVMGTHQ	15	HLA-DRB1*09:01	0.3	0.7221	0.23620419	–	–
Rv2659c	AFVLMAAWLAMRYGE	15	HLA-DRB1*01:01	0.16	0.7809	0.301353	–	–
Rv3879c	AAASGVPGARAAAAA	15	HLA-DQA1*05:01/DQB1*03:01	0.09	0.9853	0.787876	–	–
**CTL epitopes**								
Rv1737c	VVNFWAWNL	9	HLA-A*32:01	0.36	2.3392	–	0.55139	–
Rv1981c	DTAQATVGA	9	HLA-A*68:02	0.07	1.3115	–	0.02542	–
Rv2659c	RPDLRVHDL	9	HLA-B*07:02	0.03	2.4462	–	0.12556	–
Rv2660c	ASGGVTVGV	9	HLA-A*68:02	0.47	1.8481	–	0.17684	–
Rv3879c	TATHGANVSL	10	HLA-A*68:02	0.43	1.8613	–	0.04834	–
**B cellular epitopes**								
Rv1981c	RGDDALKRKASSVMLESFLF	20	–	–	–	–	–	0.95
Rv2659c	RRKFGRIRQFNSGRWQASYT	20	–	–	–	–	–	0.92
Rv3879c	AASVTPAAASGVPGARAAAA	20	–	–	–	–	–	0.93

### Antigenicity, Immunogenicity, Sensitization, and Toxicity of the C543P Candidate

An ideal LTBI diagnostic and prophylactic candidate should be immunogenic, antigenic, non-toxic, and non-allergenic. Herein, to clarify these characteristics of the C543P candidate, we used multiple servers or databases for analysis. The results showed that the antigenicity predicted by VaxiJen v2.0 and ANTIGENpro were 0.7501 and 0.936399, respectively. The immunogenicity of the C543P candidate predicted by the IEDB Immunogenicity server was 1.36469. These data suggested that the C543P candidate had excellent antigenicity and immunogenicity, and showed a potential ability to induce strong immune responses. Furthermore, the prediction results of AllerTOP v.2.0 and AllergenFP v.1.0 servers showed that the C543P candidate was non-allergenic, and the prediction results of Toxin Pred sever revealed that the C543P candidate was non-toxic. These results indicate that the C543P candidate is immunogenic, antigenic, non-toxic, and non-allergenic, highlighting that the C543P candidate may be a promising biomarker or vaccine for LTBI diagnosis and prevention.

### Physicochemical Properties and Solubility of C543P Candidate

The physicochemical properties of a diagnostic biomarker or a vaccine significantly impact their immunological functions (Ikai, 1980). Therefore, various physical and chemical properties of the C543P candidate were analyzed by the Expasy Protparam server, and the results showed that the C543P candidate is a protein with 367 amino acids, the weight of 39188.54 Da, the theoretical PI value of 9.74, instability index of 27.36, and fat index of 69.35 (Table 3). In addition, we also found that the estimated half-life of the C543P candidate in mammalian reticulocytes (*in vitro*), yeast (*in vivo*), and *E. coli* (*in vivo*) were 30 hours, > 20 hours, and > 10 hours, respectively (Table 3). These data suggest that the C543P candidate is very stable and has thermal stability (Table 3). Furthermore, the solubility of C543P predicted by Protein-Sol server was 0.437 (Figure 2C), which indicates that the C543P candidate had an acceptable solubility.

**TABLE 3:** Physicochemical property of C543P predicted by the Expasy Protparam.

Number of amino acids	Weight	Theoretical pI	Estimated half-life:	Instability index:	Aliphatic index	GRAVY
367 aa	39,188.54	9.74	30 h (mammalian reticulocytes, *in vitro*). > 20 hours (yeast, *in vivo*). > 10 hours (Escherichia coli, *in vivo*).	27.36	69.35	−0.16

### Secondary Structure, Tertiary Structure, and Tertiary Structure Validation of C543P Candidate

**FIGURE 3:**
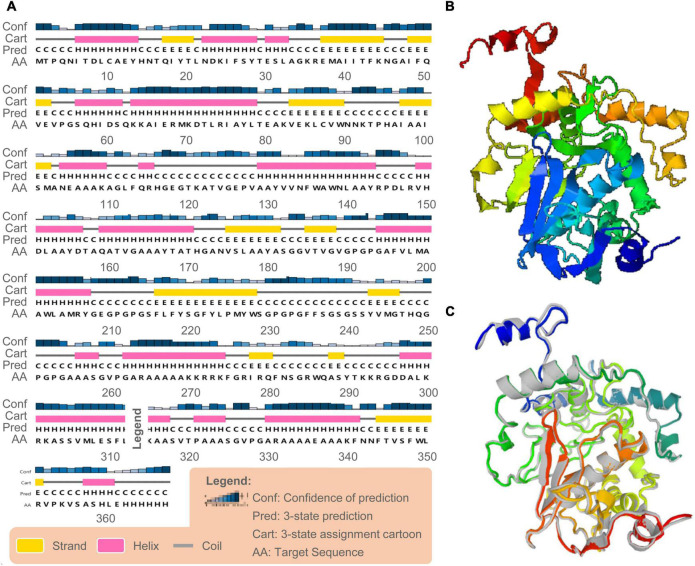
Secondary and tertiary structure prediction of the C543P candidate. **(A)** The C543P candidate was predicted to contain 43.6% α-helices, 15.8% β-strand, and 40.6% random coils by the PSIPRED server. **(B)** The 3D model of the C543P candidate was obtained on the I-TASSER server. **(C)** The 3D model of the peptide molecule after optimization by the Galaxy refining server, and the colored structure was superimposed on the rough gray model.

Based on the PSIPRED server and Prabi server, we presented the secondary structure of the C543P candidate (Figure 3A). The C543P candidate contains 43.6% α-helix, 15.8% β-strand, and 40.6% random coil. Subsequently, we predicted the 3D structures of the five 3D models designed by the I-TASSER server. The results showed that the Z scores of the five 3D models were between 0.82 and 9.12. Their confidence scores (C-score) were −1.55, −2.36, −2.33, −2.30, and −2.42, respectively. We selected the optimal 3D model with C-score = −2.36 to simulate the 3D structure of the C543P candidate (Figure 3B). We found that under this 3D model, the TM score of the C543P candidate was 0.52 ± 0.15, and the expected root-mean-square deviation (RMSD) was 10.2 ± 4.6A. The TM-value has been recommended as a calculating scale for the structural resemblance among the structures. The TM-value was suggested to address the issue of RMSD, which is delicate to native errors. Then, the GalaxyRefine web server was used to optimize the 3D model of the C543P candidate. GalaxyRefine web server can improve the consistency of modeled proteins to obtain higher quality prediction results. Through loop refinement and energy minimization on the rough model, we again obtained five optimized 3D models, from which model 2 was selected for further study (Figure 3C). The GDT-HA value of this model was 0.9469, RMSD value was 0.429, MolProbity value was 2.13, Clash score was 10.7, low Rotamers was 0.7, and Rama favored was 88.8.

**FIGURE 4:**
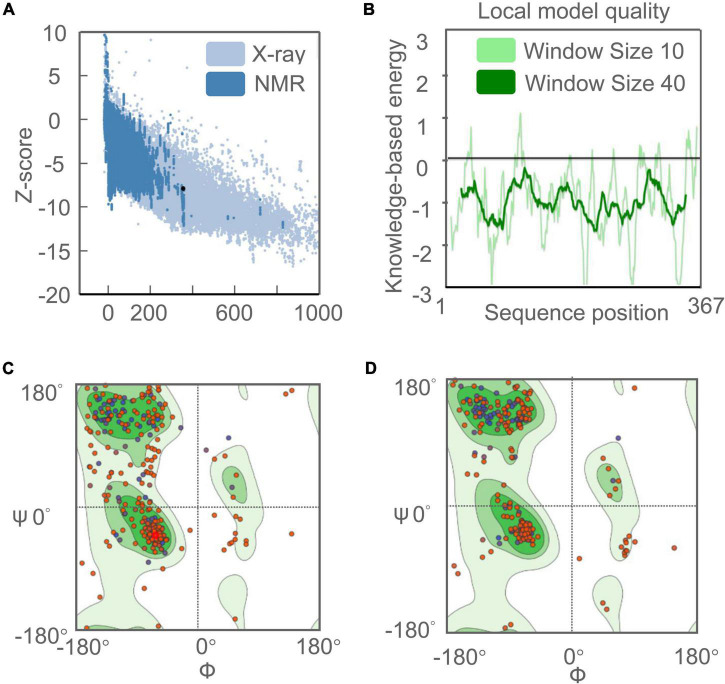
Evaluation and validation of the tertiary structure model of the C543P candidate. **(A)** Z-score predicted by ProSA-web server: −7.9. **(B)** Energy map of C543P peptide molecule validation. **(C)** Ramachandran diagram analysis of C543P peptide molecule before optimization shows Favored region: 75.39%, outliers region: 7.85%, rotamer region: 9.70%. **(D)** Ramachandran diagram of C543P peptide molecule optimization: Favored region: 88.77%, outliers region: 1.92%, rotamer region: 0.37%.

ProSA-web server and ERRAT-web server were used to verify the quality and potential errors in the 3D model of the C543P candidate. The results showed that the Z score of the optimized C543P candidate was −7.9 (Figure 4A), and the energy map was shown in Figure 4B. The Overall Quality Factor of the C543P candidate was improved from 86.9436 to 91% after being optimized by the ERRAT-web server. The Ramachandran diagram showed that, before optimizing the C543P candidate, the Favored region was 75.39%, the outliers region was 7.85%, and the rotamer region was 9.70% (Figure 4C). After optimization, the Favored region was 88.77%, the outliers region was 1.92%, and the rotamer region was 0.37% (Figure 4D). Therefore, the Favored region of the C543P candidate was increased from 75.39 to 88.77%.

### Conformational B-cell Epitopes

**FIGURE 5:**
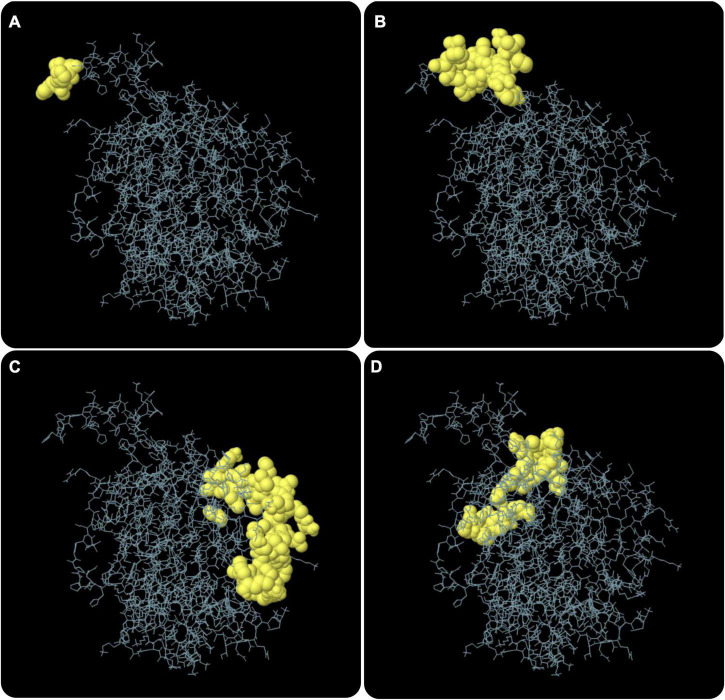
The conformation or discontinuous B cell epitope of the C543P candidate. The yellow balls in panels **(A–D)** represented 4 conformations or discontinuous B cell epitopes, respectively, and the rest were shown in gray.

According to data obtained from the ElliPro server, 193 residues were distributed on 10 putative B-cell epitopes with values ranging from 0.501 to 0.992 (Supplementary Table 5). For discontinuous epitopes predicted by the ElliPro server, epitopes with a score > 0.69 are usually selected. The conformational B-cell epitopes of the C543P candidate were estimated to have 74 residues with scores ranging from 0.709 to 0.992 (Figure 5 and Table 4).

**TABLE 4:** The conformational B cell epitopes residues of the C543P candidate predicted by the ElliPro.

No	Residues	Number of residues	Score
1	A:H365, A:H366, A:H367	3	0.992
2	A:W349, A:L350, A:R351, A:V352, A:P353, A:K354, A:V355, A:S356, A:A357, A:S358, A:H359, A:L360, A:E361, A:H362, A:H363, A:H364	16	0.918
3	A:R144, A:P145, A:D146, A:L147, A:R148, A:D151, A:L152, A:G163, A:S289, A:Y290, A:T291, A:K292, A:R294, A:G295, A:D296, A:D297, A:A298, A:L299, A:K300, A:R301, A:K302, A:A303, A:S304, A:S305, A:V306, A:M307, A:L308, A:S310, A:F311, A:L312, A:K314, A:K315	32	0.712
4	A:R206, A:G238, A:S239, A:G240, A:S241, A:S242, A:Y243, A:S318, A:V319, A:T320, A:P321, A:A322, A:A323, A:A324, A:S325, A:G326, A:V327, A:P328, A:G329, A:A330, A:R331, A:A332, A:E336	23	0.709

### Molecular Docking

**FIGURE 6:**
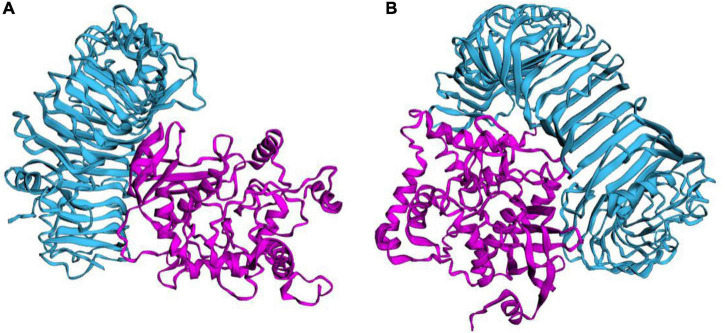
Ligand–receptor interaction. C543P was shown in purple and TLR2 **(A)** and TLR4 **(B)** were shown in blue.

This study used several servers to predict protein-protein docking to improve prediction accuracy. Firstly, the docking exploration of TLR2 and TLR4 was performed using the ClusPro 2.0 server, which generated 30 predicted complexes. Secondly, two models (1091.7 and 1102.7 kcal/mol models) with lower complex binding energies were selected for further optimization. Then, the C543P candidate was evaluated using the PatchDock server, which generated different score sheets by identifying different models. Finally, the top 10 complexes identified by the above methods were refined through the FireDock server, from which the model with the lowest required binding energy was selected. The optimization results of the TLR2-C543P complex showed that the global energy was 1.34, the attractive van der Waals energy (VdW) was −5.62, the repulsive (VdW) was 2.38, and the atomic contact energy was 6.04 (Figure 6A). The optimization results of the TLR4-C543P complex showed that the global energy was −0.35, the attractive (VdW) was −3.49, the repulsive (VdW) was 1.66, and the atomic contact energy was −1.96 (Figure 6B). The HawDock server predicted the relative binding free energies of the C543P candidate to TLR2 and TLR4 in the MM-GBSA were −4.86 and −22.58 kcal/mol, respectively.

### Molecular Dynamics Simulation Between C543P Candidate and TLRs

**FIGURE 7:**
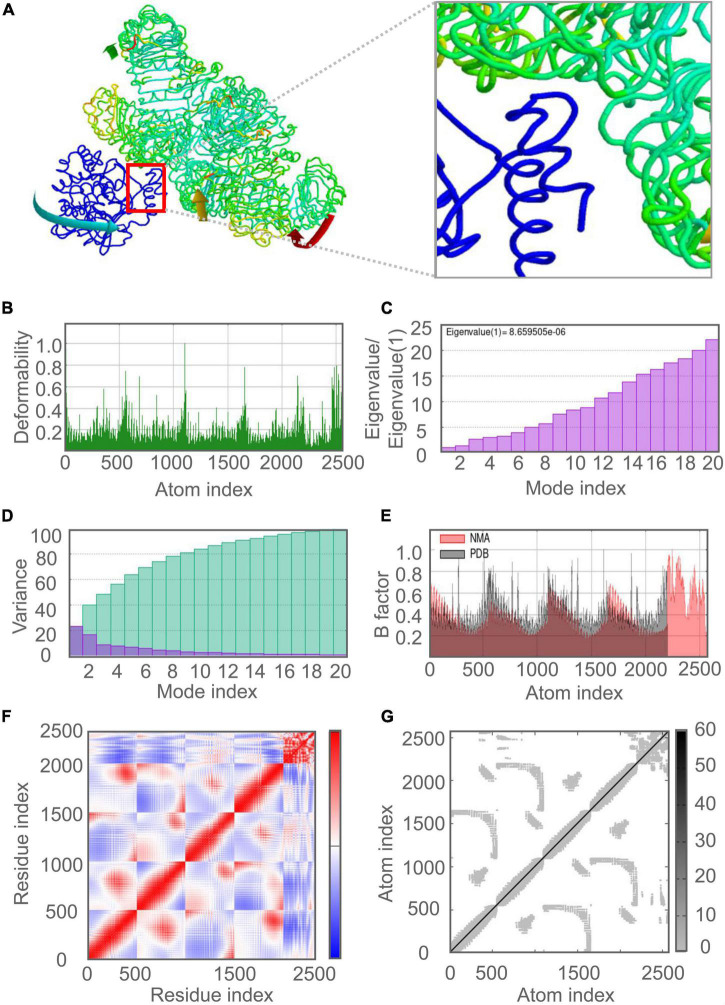
Molecular dynamics simulation of the C543P candidate and TLR2. **(A)** NMA mobility. The direction of the arrow indicates the direction of molecular motion. Blue represents the C543P molecule and green expresses the TLR2 receptor. **(B)** variability; **(C)** eigenvalues; **(D)** variance (Purple for individual variance, green for cumulative variance); **(E)** B-factor; **(F)** covariance plot (red for correlated motion, white for uncorrelated motion, the blue color indicates anti-correlated motion); **(G)** elastic network (dark gray areas indicate harder areas).

**FIGURE 8:**
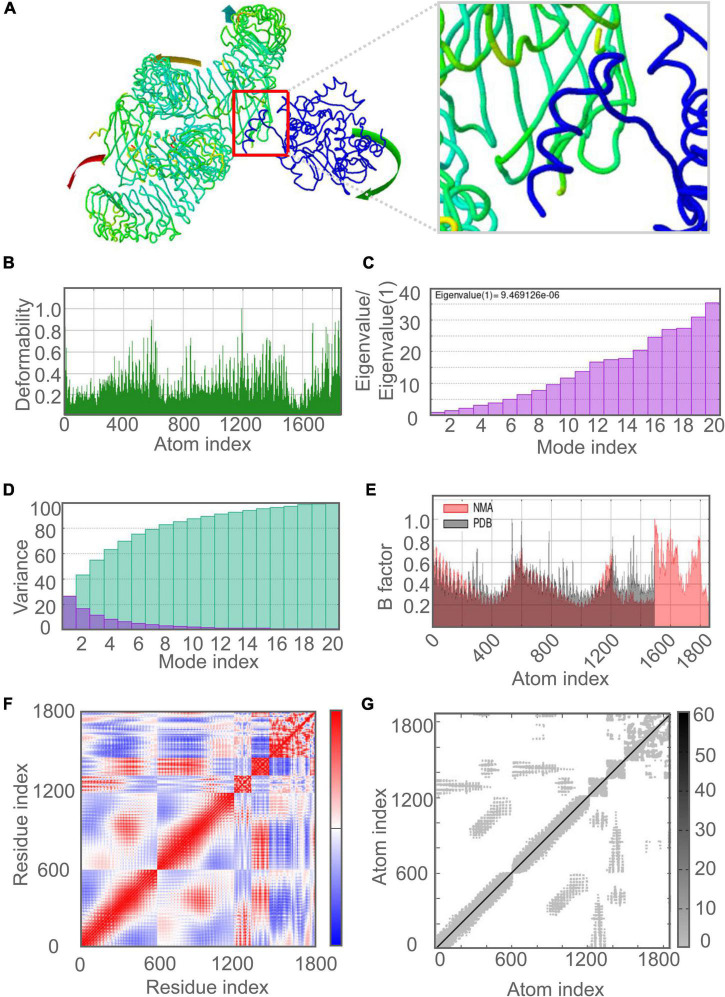
Molecular dynamics simulation of the C543P candidate and TLR4. **(A)** NMA mobility. The direction of the arrow indicates the direction of molecular motion. Blue represents the C543P molecule and green expresses the TLR2 receptor. **(B)** variability; **(C)** eigenvalues; **(D)** variance (Purple for individual variance, green for cumulative variance); **(E)** B-factor; (F) covariance plot (red for correlated motion, white for uncorrelated motion, the blue color indicates anti-correlated motion); **(G)** elastic network (dark gray areas indicate harder areas).

The molecular dynamics simulation and normal mode analysis (NMA) of the docking complex of the C543P candidate with TLR2 and TLR4 were shown in Figures 7A, 8A, respectively. Simulation prediction was performed to determine the movement of molecules and atoms in the antigenic structure. The deformability of TLR2-C543P (Figure 7B) or TLR4-C543P (Figure 8B) complex was shown with peaks in the deformable regions of the C543P candidate. The simulation results showed that the TLR2-C543P complex and TLR4-C543P complex eigenvalues were 8.659505e-06 (Figure 7C) and 9.469126e-06 (Figure 8C), respectively. Variance plots showed a cumulative or individual variance of TLR2-C543P complex (Figure 7D) and TLR4-C543P complex (Figure 8D) with green or purple, respectively. The relation of the docked TLR2-C543P complex (Figure 7E) and TLR4-C543P complex (Figure 8E) between the NMA and the PDB sector was shown by the B-factor graph. The co-variance map of the complex where the correlated motion between a pair of residues was indicated by red color, uncorrelated by white color, and anti-correlated by blue color (Figures 7F, 8F). The elastic network model showed that the docked protein molecule (C-α) atoms were interconnected with some degree of “spring” (harder springs were shown in darker gray) (Figures 7G, 8G).

### Immune Responses Induced by the Candidate C543P

**FIGURE 9:**
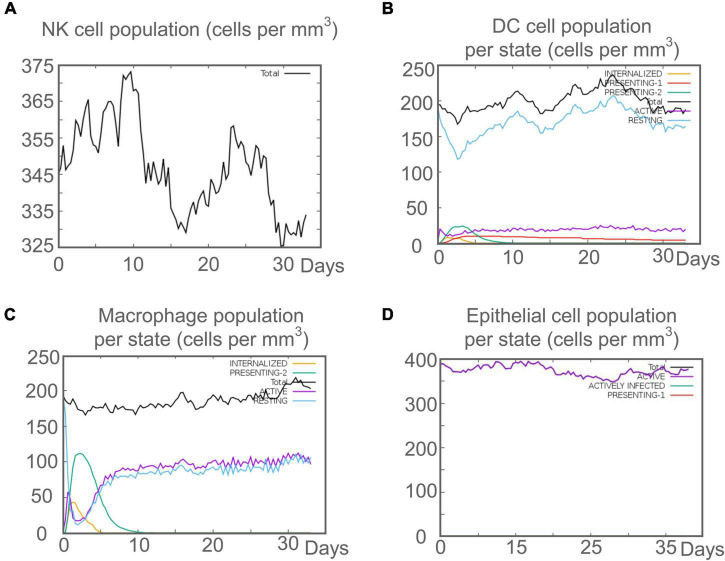
The innate immune responses induced by C543P in C-ImmSim server. **(A)** Expression of NK cells after antigen stimulation. **(B)** Expression of DC cells (black) after antigen stimulation. **(C)** Expression in macrophages (black) after antigen stimulation. **(D)** Secretion from active epithelial cells (purple) after antigen stimulation.

*Mycobacterium tuberculosis* is an intracellular pathogen, and cellular and humoral immunity play essential roles in killing and eliminating MTB. We found that the C543P candidate could effectively stimulate the innate immune system to induce immune responses in the immune simulation prediction. The results showed that after C543P stimulation, the population per state of NK cells increased in a staged and fluctuating manner, reaching a peak on day 9 (Figure 9A). The population per state of dendritic cells (DCs) reached a peak on day 23, mainly resting DCs. Interestingly, we found that presenting-2 DCs populations per state peaked on day 2 after C543P stimulation and gradually decreased (Figure 9B). In addition to DCs, macrophages can clear infected cells. Our study showed that the presenting-2 macrophages population per state started to proliferate after stimulation with C543P, peaked on day 2, and gradually decreased (Figure 9C). When presenting-2 macrophages reached their peak, the active macrophages population per state began to proliferate rapidly, reaching a peak on day 7 and maintaining a high level of activation (Figure 9C). Finally, considering that MTB first interacts with alveolar epithelial cells after entering the lungs, we also analyzed the effect of C543P on epithelial cells. The results showed that the population per state of epithelial cells would maintain a significantly high secretion level after being stimulated by C543P (Figure 9D).

**FIGURE 10:**
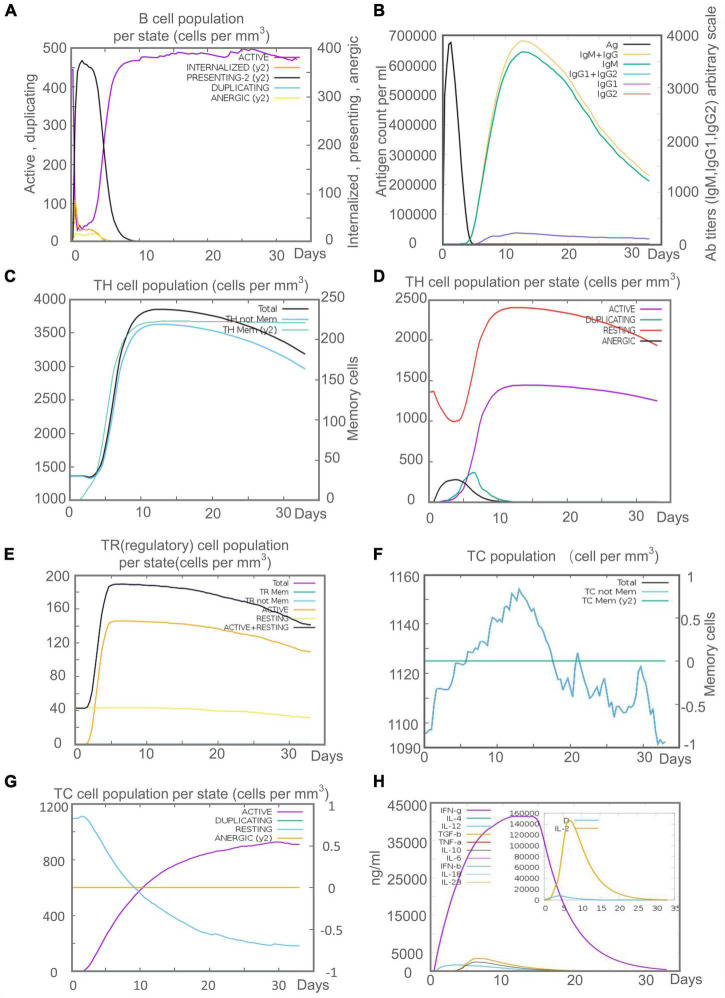
The adaptive immune responses induced by C543P in C-ImmSim server. **(A)** Active B cell (purple) secretion after antigen stimulation. **(B)** The primary B cell antibody produced after antigen stimulation is IgM + IgG (yellow) changes over time. **(C,D)** Changes in the secretion level of helper T lymphocytes and the secretion levels of helper T lymphocytes of different memory types. **(E)** Changes in Treg T cell secretion after antigen stimulation. **(F,G)** Changes in the level of CD8^+^ T secretion after antigen stimulation and the secretion of different types of CD8^+^ T cells. **(H)**. Changes in secretion levels of cytokines, mainly IFN-γ (purple) and IL-2 (yellow).

Innate immunity plays a vital role in the initial stage of MTB infection, but the clearance and killing of MTB mainly depend on the adaptive immune responses. Recent studies suggested that B lymphocyte-mediated humoral immune responses also play a role in MTB clearance (Gong et al., 2021, 2022). C-ImmSim server prediction showed that after the C543P candidate stimulation, B lymphocytes mainly performed an antigen-presenting function in the initial stage. The presenting-2 B lymphocytes population per state reached 450 cells/mm^3^ on day 2 after C543P stimulation. Then, the active B lymphocytes population per state proliferated in large numbers, reaching a peak on day 7 after C543P stimulation (Figure 10A). C543P also induced B lymphocytes to produce high levels of IgG and IgM antibodies, peaking on day 6 (Figure 10B). CD4^+^ T lymphocytes are the central effector cells that kill and clear MTB. The C-ImmSim server showed that C543P could stimulate the proliferation of memory and non-memory CD4^+^ T lymphocytes significantly, reaching a peak on day 10 (>3,500 cells/mm^3^) and then decreasing slowly (Figure 10C). The population per state of active and resting HTL peaked on day 7 and then gradually declined (Figure 10D). Regulatory T cells can regulate the immune response of helper T cells to avoid the overreaction of Th1 and Th2 cells. The results showed that the regulatory T cell population per state reached as high as 140 cells/mm^3^ on day 5 after C543P stimulation, followed by a decrease (Figure 10E). Furthermore, it is reported that CTL plays an essential role in killing the host cells infected by MTB via producing granular enzymes and nitric oxide. Herein, we found that the population per state of non-memory cytotoxic T cells gradually increased and peaked (1,150 cells/mm^3^) on day 13, and that of memory cytotoxic T cells remained at the level of 1,125 cells/mm^3^ (Figure 10F). Moreover, we also found that the population per state of active cytotoxic T lymphocytes gradually increased and reached a peak on the 30th day after stimulation. In contrast, resting cytotoxic T lymphocytes showed an opposite trend (Figure 10G). Interestingly, our results indicated that C543P could induce significantly high levels of IFN-γ and IL-2 (Figure 10H).

### Codon Optimization and *in-silico* Cloning

To predict the molecular cloning and expression levels of the C543P candidate *in vitro*, we used the Java Adaptation Tool (JACT) server to predict maximum protein expression in *E. coli* (K12). The data showed that the CAI value of the C543P candidate was 1, and the average GC content of the adapted sequence was 52.6%, indicating that the C543P candidate could be highly expressed in *E. coli*. Finally, using SnapGene software, the gene sequence of the optimized C543P candidate was inserted into the pET30a(+) plasmid to construct a recombinant plasmid (Supplementary Figure 1).

## Discussion

Rapid advances in bioinformatics, structural biology, and computational tools have revolutionized the development of diagnostic biomarkers and vaccines. The use of these tools has greatly aided in the processing and analyzing basic data on microorganisms (Kardani et al., 2020). The methods for predicting and designing diagnostic biomarkers and vaccines *in-silico* have greatly improved, and they have been applied to bacteria, viruses, fungi, and even cancers (Raoufi et al., 2020). Previous studies have designed several peptides-based vaccine candidates against MTB infection (Bibi et al., 2021; Sharma et al., 2021; Shiraz et al., 2021). The bioinformatics technologies used in these studies promote the development of TB vaccines and reduce cost and save time. However, the epitopes of these TB vaccines were predicted and selected from antigens secreted by MTB during proliferation, which means that these TB vaccines may not fight against LTBI. Thus, we designed a PPM named C543P based on epitopes selected from five LTBI-RD-associated antigens, including Rv1737c, Rv1981c, Rv2659c, Rv2660c, and Rv3879c. The C543P candidate might be a promising candidate biomarker for the diagnosis and prevention of LTBI.

LTBI is a dynamic equilibrium state achieved by mutual antagonism between host immune function and invasiveness of MTB (Gong and Wu, 2021). The activation of CD4^+^ T cells and CD8^+^ T cells depends on recognizing the MHC-II and MHC-I molecules on the surface of antigen-presenting cells. Therefore, the C543P candidate has the following advantages: (1) As a preventive vaccine for LTBI, the C543P can activate CD4^+^ T cells and CD8^+^ T cells to produce high levels of cytokines such as IFN-γ and IL-2, enhancing the anti-MTB activity of macrophages (Bertholet et al., 1950), and inhibit the transformation of LTBI to ATB. (2) As a biomarker for the differential diagnosis of LTBI, C543P could stimulate the peripheral blood mononuclear cells (PBMCs) of the LTBI population to produce significantly high levels of IFN-γ in a short time, thereby separating the LTBI population from ATB patients.

The enzyme-linked immunosorbent spot (ELISpot) assay is a commonly used method to detect human-specific T cells, accurately assessing the number of T cells with IFN-γ releasing ability. Therefore, the C543P was constructed based on immunodominant epitopes inducing a high level of IFN-γ in individuals with LTBI. However, differences in racial genetic background constitute a significant obstacle to developing epitope-based diagnostic markers and vaccines. HLA is highly polymorphic, thus, selecting immunodominant epitopes that can be recognized by numerous HLAs is beneficial to improving the coverage of the C543P candidate and avoiding racial bias (Bui et al., 2006). Therefore, when predicting HTL and CTL epitopes, we choose the default HLA-I and HLA-II alleles most frequently distributed in the population from the IEDB database. On this basis, the immunodominant epitopes with better antigenicity and immunogenicity were selected. In addition, the addition of B cell epitopes allows C543P to activate B lymphocytes more efficiently to induce humoral immunity.

The C543P candidate also contains several vital adjuvants and TLR agonists to enhance its ability to induce immune responses. These TLR agonists used in C543P can be recognized by cognate TLRs on different immune cells and polarize naive CD4^+^ T cells through the Th1 pathway to induce an immune response (Kawai and Akira, 2010). Our prediction data showed that the antigenicity of C543P was 0.7501, and the immunogenicity was 1.36469. These data indicate that the C543P has a good antigenicity and can induce a high level of immune response. Furthermore, we found that C543P was a non-toxic, non-allergenic, and stable candidate for LTBI diagnosis and prevention. The limitation of the C543P is that the solubility of C543P was 0.437, so it is necessary to improve its solubility in the subsequent optimization for better cloning and expression in *E. coli*.

The secondary structure of C543P mainly contains 43.6% α-helix, 15.8% β-strand, and 40.6% random coil. Naturally unfolded protein regions and α-helices are considered essential types of “structural antigens.” Thus, the increase of these two structures contributes to the recognition of antibodies induced after infection (Corradin et al., 2007). It was found that 88.77% of the amino acid residues of C543P were in the Favored region, indicating that the entire model’s prediction quality was acceptable. The main targets of host recognition of MTB are TLR2 and TLR4, so adding TLR2 and TLR4 agonists as adjuvants in the design of C543P can significantly improve the presentation efficiency of C543P. We tested the binding ability of C543P to TLR2 and TLR4, and the results showed that the C543P has a high binding affinity, suggesting that the C543P can be efficiently recognized by TLR2 and TLR4 and induce a more robust immune response.

Whether as a biomarker for differential diagnosis of LTBI or as a vaccine for LTBI prevention, the ability of C543P to induce immune responses in T and B lymphocytes determines its potential value. Previous studies have shown that macrophages clear MTB through oxygen, chloride, cytokine production, phagosome acidification, and intracellular MTB autophagy (Liu et al., 2017). In addition, some macrophages phagocytose MTB present antigens or epitopes to T cells for recognition and activate adaptive immune responses (Gong et al., 2022). Our immune prediction found that the C543P could induce effective innate and adaptive immune responses in humans. As the first line of defense against MTB infection, alveolar epithelial cells can use MHC-II molecules to influence the lungs’ immune system. Furthermore, the high level of alveolar epithelial cells can promote the secretion of cytokines by HTL to kill MTB (Shenoy, 2021). Our study found that macrophages play an essential role in C543P antigen presentation. The population per state of macrophages exceeded 100 cells/mm^3^ on the second day after C543P stimulation. The Th1 subset of CD4^+^ T lymphocytes plays a central role in the host killing of MTB infection, mainly secreting IFN-γ, IL-2, IL-12, and TNF-α (Boom et al., 2003). The results of C-ImmSim server prediction found that the C543P induced T lymphocytes to secrete high levels of Th1-type cytokines such as IFN-γ and IL-2, indicating that the C543P mainly induced the secretion of Th1-type cytokines. Apart from this, the C543P can also effectively activate CD8^+^ T lymphocytes, and the population per state of CD8^+^ T cells reached 1155 cells/mm^3^ on the 13th day after stimulation. These massively proliferated and activated CD8^+^ T cells can effectively lyse and kill cells infected with MTB by secreting a large amount of granzyme B, perforin, and other cytotoxic factors (Kumar and Babu, 2017).

## Conclusion

In summary, a novel diagnostic biomarker or vaccine candidate named C543P was designed in this study. The C543P candidate consists of four HTL, five CTL, and three B cell epitopes as well as adjuvant or agonists. Our results showed that C543P was highly antigenic and immunogenic while non-sensitizing and non-toxic. Furthermore, it was found that the C543P candidate had a good affinity with both TLR2 and TLR4, and the kinetic simulation showed that C543P had good stability and could promote its interaction with TLRs. In addition, the C543P candidate can induce high levels of innate and adaptive immune responses in humans, characterized by markedly higher levels of the Th1-type cytokines such as IFN-γ and IL-2. This study provides a promising diagnostic biomarker and a vaccine candidate for diagnosing and preventing LTBI.

## Data Availability Statement

The original contributions presented in the study are included in the article/Supplementary Material, further inquiries can be directed to the corresponding authors.

## Author Contributions

WG and LW: conceptualization and writing—review and editing. PC: data curation, formal analysis and writing—original draft. WG: funding acquisition. PC and WG: methodology and software. All authors contributed to the article and approved the submitted version.

## Conflict of Interest

The authors declare that the research was conducted in the absence of any commercial or financial relationships that could be construed as a potential conflict of interest.

## Publisher’s Note

All claims expressed in this article are solely those of the authors and do not necessarily represent those of their affiliated organizations, or those of the publisher, the editors and the reviewers. Any product that may be evaluated in this article, or claim that may be made by its manufacturer, is not guaranteed or endorsed by the publisher.

## Footnotes

1
https://mycobrowser.epfl.ch/

2
http://tools.iedb.org/mhcii/

3
http://www.ddg-pharmfac.net/vaxijen/VaxiJen/VaxiJen.html

4
http://crdd.osdd.net/raghava/ifnepitope/index.php

5
http://tools.iedb.org/mhci/

6
http://tools.iedb.org/immunogenicity/

7
https://webs.iiitd.edu.in/raghava/abcpred/ABC_submission.html

8
http://scratch.proteomics.ics.uci.edu/

9
http://www.ddg-pharmfac.net/AllerTOP/

10
http://ddg-pharmfac.net/AllergenFP/

11
https://web.expasy.org/protparam/

12
https://protein-sol.manchester.ac.uk/

13
http://bioinf.cs.ucl.ac.uk/psipred/

14
https://npsa-prabi.ibcp.fr/cgi-bin/npsa_automat.pl?page=/NPSA/npsa_gor4.html

15
https://zhanggroup.org//I-TASSER/

16
https://galaxy.seoklab.org/cgi-bin/submit.cgi?type=REFINE

17
https://prosa.services.came.sbg.ac.at/prosa.php

18
https://saves.mbi.ucla.edu/

19
https://swissmodel.expasy.org/assess

20
http://tools.iedb.org/ellipro/

21
https://www.ncbi.nlm.nih.gov/structure/

22
https://cluspro.bu.edu/home.php

23
https://bioinfo3d.cs.tau.ac.il/PatchDock/php.php

24
http://bioinfo3d.cs.tau.ac.il/FireDock/

25
http://cadd.zju.edu.cn/hawkdock/

26
https://imods.iqfr.csic.es/

27
https://150.146.2.1/C-IMMSIM/index.php

28
http://www.jcat.de/Result.jsp

## Supplementary Material

Supplementary Figure 1 The polypeptide molecular sequence was electronically cloned into the pET30a expression vector by SnapGene software. The diagnostic antigen is the gene coding for the insertion of antigen molecules, and the rest is the expression vector.

Supplementary Table 1 The detailed information of the whole HTL epitopes predicted by IEDB database. The potential HTL epitopes were identified by adjusted rank, antigenicity score, and IFN score, and marked with yellow color.

Supplementary Table 2 The detailed information of the whole CTL epitopes predicted by IEDB database.

Supplementary Table 3 The potential CTL epitopes identified by immunogenicity and antigenicity.

Supplementary Table 4 The detailed information of linear B-cell epitopes predicted by the ABCpred. The epitopes selected for further study were marked with yellow color.

Supplementary Table 5 The detailed information of conformational B cell epitopes residues of C543P predicted by the ElliPro.

## Abbreviations

ACC: auto-cross-covariance
ATB: active tuberculosis
ANN: Artificial Neural Network
BCG: Bacillus Calmette–Guérin
CTL: cytotoxic T lymphocytes
CFP10: culture filtrate protein 10
CAI: codon adaptation index
DCs: Dendritic cells
E. coli: Escherichia coli
EAST-6: early secreted antigenic target 6
ELISpot: enzyme-linked immunosorbent spot
ELISA: enzyme-linked immunosorbent assay
GRAVY: grand average of hydrophilicity
HIV: human immunodeficiency virus
HLA: Human leukocyte antigen
HTL: helper T lymphocytes
IFN-γ: interferon gamma
IGRA: interferon gamma release assays
IEDB: Immune Epitope Database
JACT: Java Adaptation Tool
kNN: k nearest neighbors
LTBI: latent tuberculosis infection
MTB: *Mycobacterium tuberculosis*
MDR-TB: multidrug-resistant TB
MHC: major histocompatibility complex
NIAID: National Institute of Allergy and Infectious Diseases
NMA: normal mode analysis
PopAvrSol: population average for the experimental dataset
PBMCs: peripheral blood mononuclear cells
PPD: purified protein derivative
QFT-Plus: QuantiFERON-TB Gold-Plus
QFT-GIT: QuantiFERON-TB Gold In-Tube
RR-TB: rifampicin-resistant TB
RMSD: root mean square deviation
RD1: Region of Difference 1
T-SPOT.TB: T-cell spot of TB assay
TST: tuberculin skin test
TB: Tuberculosis
TLR: toll-like receptor
VdW: van der Waals energy
WHO: World Health Organization.
