# Supplementary material for: In silico Analysis of Peptide-Based Biomarkers for the Diagnosis and Prevention of Latent Tuberculosis Infection
Source: Front Microbiol. 2022 Jun 28;13:947852. doi: 10.3389/fmicb.2022.947852 (PMC9273951; doi:10.3389/fmicb.2022.947852)
Supplement: Supplementary Figure 1 — The polypeptide molecular sequence was electronically cloned into the pET30a expression vector by SnapGene software. The diagnostic antigen is the gene coding for the insertion of antigen molecules, and the rest is the expression vector. [file Data_Sheet_1.zip › Supplementary Files/Table S1.docx]

Rv1737c HTL epitope

| allele | start | end | length | method | peptide | percentile_rank | adjusted_rank | antigenicity score |
| --- | --- | --- | --- | --- | --- | --- | --- | --- |
| HLA-DRB1*15:01 | 97 | 111 | 15 | Consensus (smm/nn/sturniolo) | YALLVFFGLFLGVAG | 0.37 | 0.37 | 0.6899 |
| HLA-DRB1*04:01 | 29 | 43 | 15 | Consensus (smm/nn/sturniolo) | STSYARDMSLSSAEA | 0.29 | 0.29 | 0.6436 |
| HLA-DRB1*15:01 | 96 | 110 | 15 | Consensus (smm/nn/sturniolo) | SYALLVFFGLFLGVA | 0.37 | 0.37 | 0.6347 |
| HLA-DRB1*04:01 | 30 | 44 | 15 | Consensus (smm/nn/sturniolo) | TSYARDMSLSSAEAS | 0.32 | 0.32 | 0.6175 |
| HLA-DRB1*15:01 | 95 | 109 | 15 | Consensus (smm/nn/sturniolo) | GSYALLVFFGLFLGV | 0.37 | 0.37 | 0.6136 |
| HLA-DPA1*02:01/DPB1*14:01 | 245 | 259 | 15 | NetMHCIIpan | GARTAGFALAAVLAR | 0.39 | 0.39 | 0.5954 |
| HLA-DRB1*04:01 | 28 | 42 | 15 | Consensus (smm/nn/sturniolo) | LSTSYARDMSLSSAE | 0.33 | 0.33 | 0.5528 |
| HLA-DPA1*01:03/DPB1*04:01 | 144 | 158 | 15 | NetMHCIIpan | TALSAFFTPRFVRWF | 0.35 | 0.35 | 0.4686 |
| HLA-DRB1*01:01 | 273 | 287 | 15 | Consensus (comb.lib./smm/nn) | HVVLASLAGTALLAF | 0.24 | 0.24 | 0.4651 |
| HLA-DQA1*05:01/DQB1*03:01 | 162 | 176 | 15 | Consensus (comb.lib./smm/nn) | TTHAIVAAALASTAV | 0.15 | 0.15 | 0.4531 |
| HLA-DRB1*04:01 | 27 | 41 | 15 | Consensus (smm/nn/sturniolo) | PLSTSYARDMSLSSA | 0.34 | 0.34 | 0.4369 |
| HLA-DPA1*02:01/DPB1*14:01 | 249 | 263 | 15 | NetMHCIIpan | AGFALAAVLARPVGG | 0.2 | 0.2 | 0.402 |
| HLA-DPA1*02:01/DPB1*14:01 | 246 | 260 | 15 | NetMHCIIpan | ARTAGFALAAVLARP | 0.21 | 0.21 | 0.384 |
| HLA-DQA1*05:01/DQB1*03:01 | 164 | 178 | 15 | Consensus (comb.lib./smm/nn) | HAIVAAALASTAVVA | 0.15 | 0.15 | 0.3602 |
| HLA-DRB1*15:01 | 94 | 108 | 15 | Consensus (smm/nn/sturniolo) | MGSYALLVFFGLFLG | 0.39 | 0.39 | 0.3589 |
| HLA-DRB1*01:01 | 361 | 375 | 15 | Consensus (comb.lib./smm/nn) | NDYTVGLLLLVATAL | 0.1 | 0.1 | 0.3581 |
| HLA-DQA1*05:01/DQB1*03:01 | 165 | 179 | 15 | Consensus (comb.lib./smm/nn) | AIVAAALASTAVVAM | 0.21 | 0.21 | 0.3574 |
| HLA-DPA1*02:01/DPB1*14:01 | 247 | 261 | 15 | NetMHCIIpan | RTAGFALAAVLARPV | 0.12 | 0.12 | 0.3479 |
| HLA-DRB1*01:01 | 274 | 288 | 15 | Consensus (comb.lib./smm/nn) | VVLASLAGTALLAFA | 0.24 | 0.24 | 0.3173 |
| HLA-DQA1*05:01/DQB1*03:01 | 161 | 175 | 15 | Consensus (comb.lib./smm/nn) | FTTHAIVAAALASTA | 0.15 | 0.15 | 0.3006 |
| HLA-DQA1*05:01/DQB1*03:01 | 163 | 177 | 15 | Consensus (comb.lib./smm/nn) | THAIVAAALASTAVV | 0.15 | 0.15 | 0.2987 |
| HLA-DQA1*05:01/DQB1*03:01 | 166 | 180 | 15 | Consensus (comb.lib./smm/nn) | IVAAALASTAVVAMV | 0.32 | 0.32 | 0.292 |
| HLA-DRB1*01:01 | 270 | 284 | 15 | Consensus (comb.lib./smm/nn) | APRHVVLASLAGTAL | 0.38 | 0.38 | 0.2872 |
| HLA-DRB1*01:01 | 362 | 376 | 15 | Consensus (comb.lib./smm/nn) | DYTVGLLLLVATALV | 0.01 | 0.01 | 0.2756 |
| HLA-DQA1*01:02/DQB1*06:02 | 276 | 290 | 15 | Consensus (comb.lib./smm/nn) | LASLAGTALLAFAAA | 0.41 | 0.41 | 0.2678 |
| HLA-DRB1*01:01 | 363 | 377 | 15 | Consensus (comb.lib./smm/nn) | YTVGLLLLVATALVA | 0.01 | 0.01 | 0.2301 |
| HLA-DPA1*01:03/DPB1*04:01 | 146 | 160 | 15 | NetMHCIIpan | LSAFFTPRFVRWFGL | 0.43 | 0.43 | 0.2222 |
| HLA-DQA1*05:01/DQB1*03:01 | 160 | 174 | 15 | Consensus (comb.lib./smm/nn) | LFTTHAIVAAALAST | 0.27 | 0.27 | 0.2165 |
| HLA-DPA1*02:01/DPB1*14:01 | 248 | 262 | 15 | NetMHCIIpan | TAGFALAAVLARPVG | 0.16 | 0.16 | 0.2079 |
| HLA-DRB1*01:01 | 364 | 378 | 15 | Consensus (comb.lib./smm/nn) | TVGLLLLVATALVAC | 0.01 | 0.01 | 0.1701 |
| HLA-DPA1*01:03/DPB1*02:01 | 296 | 310 | 15 | Consensus (comb.lib./smm/nn) | EVWSAATFITLAVCL | 0.47 | 0.47 | 0.1464 |
| HLA-DPA1*01:03/DPB1*02:01 | 293 | 307 | 15 | Consensus (comb.lib./smm/nn) | PPPEVWSAATFITLA | 0.47 | 0.47 | 0.1031 |
| HLA-DPA1*01:03/DPB1*02:01 | 294 | 308 | 15 | Consensus (comb.lib./smm/nn) | PPEVWSAATFITLAV | 0.45 | 0.45 | 0.0708 |
| HLA-DRB1*01:01 | 271 | 285 | 15 | Consensus (comb.lib./smm/nn) | PRHVVLASLAGTALL | 0.38 | 0.38 | 0.0685 |
| HLA-DRB1*01:01 | 272 | 286 | 15 | Consensus (comb.lib./smm/nn) | RHVVLASLAGTALLA | 0.38 | 0.38 | 0.0588 |
| HLA-DRB1*01:01 | 366 | 380 | 15 | Consensus (comb.lib./smm/nn) | GLLLLVATALVACTY | 0.24 | 0.24 | 0.0469 |
| HLA-DRB1*01:01 | 365 | 379 | 15 | Consensus (comb.lib./smm/nn) | VGLLLLVATALVACT | 0.1 | 0.1 | 0.0388 |
| HLA-DPA1*02:01/DPB1*14:01 | 250 | 264 | 15 | NetMHCIIpan | GFALAAVLARPVGGW | 0.3 | 0.3 | 0.032 |
| HLA-DPA1*01:03/DPB1*02:01 | 292 | 306 | 15 | Consensus (comb.lib./smm/nn) | QPPPEVWSAATFITL | 0.47 | 0.47 | -0.007 |
| HLA-DPA1*01:03/DPB1*02:01 | 295 | 309 | 15 | Consensus (comb.lib./smm/nn) | PEVWSAATFITLAVC | 0.45 | 0.45 | -0.008 |
| HLA-DQA1*05:01/DQB1*03:01 | 213 | 227 | 15 | Consensus (comb.lib./smm/nn) | FLYAIVFGGFVAFSN | 0.2 | 0.2 | -0.0546 |
| HLA-DQA1*05:01/DQB1*03:01 | 214 | 228 | 15 | Consensus (comb.lib./smm/nn) | LYAIVFGGFVAFSNY | 0.37 | 0.37 | -0.1965 |

Rv1981c HTL epitope

| allele | start | end | length | method | peptide | percentile_rank | adjusted_rank | antigenicity score | IFN score |
| --- | --- | --- | --- | --- | --- | --- | --- | --- | --- |
| HLA-DPA1*01:03/DPB1*02:01 | 152 | 166 | 15 | Consensus (comb.lib./smm/nn) | KRKASSVMLESFLFY | 0.33 | 0.33 | 0.9047 | NEGATIVE |
| HLA-DPA1*02:01/DPB1*14:01 | 152 | 166 | 15 | NetMHCIIpan | KRKASSVMLESFLFY | 0.33 | 0.33 | 0.9047 | NEGATIVE |
| HLA-DPA1*01:03/DPB1*04:01 | 162 | 176 | 15 | NetMHCIIpan | SFLFYSGFYLPMYWS | 0.01 | 0.01 | 0.8237 | 1 |
| HLA-DPA1*02:01/DPB1*01:01 | 162 | 176 | 15 | Consensus (comb.lib./smm/nn) | SFLFYSGFYLPMYWS | 0.17 | 0.17 | 0.8237 | 1 |
| HLA-DQA1*01:01/DQB1*05:01 | 162 | 176 | 15 | Consensus (comb.lib./smm/nn) | SFLFYSGFYLPMYWS | 0.25 | 0.25 | 0.8237 | 1 |
| HLA-DPA1*01:03/DPB1*02:01 | 162 | 176 | 15 | Consensus (comb.lib./smm/nn) | SFLFYSGFYLPMYWS | 0.3 | 0.3 | 0.8237 | 1 |
| HLA-DPA1*01:03/DPB1*04:01 | 163 | 177 | 15 | NetMHCIIpan | FLFYSGFYLPMYWSS | 0.02 | 0.02 | 0.8181 | 1 |
| HLA-DQA1*01:01/DQB1*05:01 | 163 | 177 | 15 | Consensus (comb.lib./smm/nn) | FLFYSGFYLPMYWSS | 0.18 | 0.18 | 0.8181 | 1 |
| HLA-DPA1*01:03/DPB1*04:01 | 161 | 175 | 15 | NetMHCIIpan | ESFLFYSGFYLPMYW | 0.01 | 0.01 | 0.757 | 1 |
| HLA-DPA1*02:01/DPB1*01:01 | 161 | 175 | 15 | Consensus (comb.lib./smm/nn) | ESFLFYSGFYLPMYW | 0.09 | 0.09 | 0.757 | 1 |
| HLA-DQA1*01:01/DQB1*05:01 | 161 | 175 | 15 | Consensus (comb.lib./smm/nn) | ESFLFYSGFYLPMYW | 0.24 | 0.24 | 0.757 | 1 |
| HLA-DPA1*01:03/DPB1*02:01 | 161 | 175 | 15 | Consensus (comb.lib./smm/nn) | ESFLFYSGFYLPMYW | 0.25 | 0.25 | 0.757 | 1 |
| HLA-DRB1*09:01 | 299 | 313 | 15 | Consensus (comb.lib./smm/nn) | FFSGSGSSYVMGTHQ | 0.3 | 0.3 | 0.7221 | 0.23620419 |
| HLA-DPA1*02:01/DPB1*14:01 | 151 | 165 | 15 | NetMHCIIpan | LKRKASSVMLESFLF | 0.07 | 0.07 | 0.7179 | NEGATIVE |
| HLA-DPA1*01:03/DPB1*02:01 | 153 | 167 | 15 | Consensus (comb.lib./smm/nn) | RKASSVMLESFLFYS | 0.35 | 0.35 | 0.6229 |  |
| HLA-DPA1*01:03/DPB1*04:01 | 164 | 178 | 15 | NetMHCIIpan | LFYSGFYLPMYWSSR | 0.11 | 0.11 | 0.6032 |  |
| HLA-DQA1*01:01/DQB1*05:01 | 164 | 178 | 15 | Consensus (comb.lib./smm/nn) | LFYSGFYLPMYWSSR | 0.3 | 0.3 | 0.6032 |  |
| HLA-DPA1*01:03/DPB1*04:01 | 160 | 174 | 15 | NetMHCIIpan | LESFLFYSGFYLPMY | 0.01 | 0.01 | 0.5945 |  |
| HLA-DPA1*02:01/DPB1*01:01 | 160 | 174 | 15 | Consensus (comb.lib./smm/nn) | LESFLFYSGFYLPMY | 0.09 | 0.09 | 0.5945 |  |
| HLA-DQA1*01:01/DQB1*05:01 | 160 | 174 | 15 | Consensus (comb.lib./smm/nn) | LESFLFYSGFYLPMY | 0.17 | 0.17 | 0.5945 |  |
| HLA-DPA1*01:03/DPB1*02:01 | 160 | 174 | 15 | Consensus (comb.lib./smm/nn) | LESFLFYSGFYLPMY | 0.25 | 0.25 | 0.5945 |  |
| HLA-DPA1*02:01/DPB1*14:01 | 149 | 163 | 15 | NetMHCIIpan | DALKRKASSVMLESF | 0.14 | 0.14 | 0.538 |  |
| HLA-DPA1*01:03/DPB1*02:01 | 157 | 171 | 15 | Consensus (comb.lib./smm/nn) | SVMLESFLFYSGFYL | 0.04 | 0.04 | 0.4263 |  |
| HLA-DPA1*02:01/DPB1*14:01 | 150 | 164 | 15 | NetMHCIIpan | ALKRKASSVMLESFL | 0.07 | 0.07 | 0.4166 |  |
| HLA-DPA1*01:03/DPB1*04:01 | 159 | 173 | 15 | NetMHCIIpan | MLESFLFYSGFYLPM | 0.01 | 0.01 | 0.4006 |  |
| HLA-DPA1*02:01/DPB1*01:01 | 159 | 173 | 15 | Consensus (comb.lib./smm/nn) | MLESFLFYSGFYLPM | 0.09 | 0.09 | 0.4006 |  |
| HLA-DPA1*01:03/DPB1*02:01 | 159 | 173 | 15 | Consensus (comb.lib./smm/nn) | MLESFLFYSGFYLPM | 0.25 | 0.25 | 0.4006 |  |
| HLA-DPA1*01:03/DPB1*04:01 | 158 | 172 | 15 | NetMHCIIpan | VMLESFLFYSGFYLP | 0.18 | 0.18 | 0.341 |  |
| HLA-DPA1*01:03/DPB1*02:01 | 158 | 172 | 15 | Consensus (comb.lib./smm/nn) | VMLESFLFYSGFYLP | 0.23 | 0.23 | 0.341 |  |
| HLA-DPA1*02:01/DPB1*01:01 | 158 | 172 | 15 | Consensus (comb.lib./smm/nn) | VMLESFLFYSGFYLP | 0.29 | 0.29 | 0.341 |  |
| HLA-DPA1*01:03/DPB1*02:01 | 154 | 168 | 15 | Consensus (comb.lib./smm/nn) | KASSVMLESFLFYSG | 0.33 | 0.33 | 0.3396 |  |
| HLA-DPA1*01:03/DPB1*02:01 | 155 | 169 | 15 | Consensus (comb.lib./smm/nn) | ASSVMLESFLFYSGF | 0.24 | 0.24 | 0.3392 |  |
| HLA-DRB3*02:02 | 254 | 268 | 15 | NetMHCIIpan | LPYMRYNANKALANL | 0.01 | 0.01 | 0.3316 |  |
| HLA-DRB3*02:02 | 257 | 271 | 15 | NetMHCIIpan | MRYNANKALANLGYQ | 0.15 | 0.15 | 0.3103 |  |
| HLA-DRB3*02:02 | 253 | 267 | 15 | NetMHCIIpan | VLPYMRYNANKALAN | 0.11 | 0.11 | 0.2852 |  |
| HLA-DPA1*01:03/DPB1*02:01 | 156 | 170 | 15 | Consensus (comb.lib./smm/nn) | SSVMLESFLFYSGFY | 0.15 | 0.15 | 0.2306 |  |
| HLA-DRB1*04:01 | 106 | 120 | 15 | Consensus (smm/nn/sturniolo) | KSYSSIFSTLCSTKQ | 0.33 | 0.33 | 0.2156 |  |
| HLA-DRB3*02:02 | 255 | 269 | 15 | NetMHCIIpan | PYMRYNANKALANLG | 0.01 | 0.01 | 0.1937 |  |
| HLA-DRB3*02:02 | 252 | 266 | 15 | NetMHCIIpan | DVLPYMRYNANKALA | 0.49 | 0.49 | 0.1165 |  |
| HLA-DRB3*02:02 | 256 | 270 | 15 | NetMHCIIpan | YMRYNANKALANLGY | 0.01 | 0.01 | 0.0557 |  |
| HLA-DQA1*01:01/DQB1*05:01 | 243 | 257 | 15 | Consensus (comb.lib./smm/nn) | LYDELGWTDDVLPYM | 0.12 | 0.12 | 0.0301 |  |
| HLA-DQA1*01:01/DQB1*05:01 | 245 | 259 | 15 | Consensus (comb.lib./smm/nn) | DELGWTDDVLPYMRY | 0.4 | 0.4 | 0.0064 |  |
| HLA-DRB1*04:01 | 107 | 121 | 15 | Consensus (smm/nn/sturniolo) | SYSSIFSTLCSTKQI | 0.24 | 0.24 | -0.0127 |  |
| HLA-DQA1*01:01/DQB1*05:01 | 242 | 256 | 15 | Consensus (comb.lib./smm/nn) | DLYDELGWTDDVLPY | 0.13 | 0.13 | -0.0552 |  |
| HLA-DQA1*01:01/DQB1*05:01 | 241 | 255 | 15 | Consensus (comb.lib./smm/nn) | HDLYDELGWTDDVLP | 0.14 | 0.14 | -0.1047 |  |
| HLA-DQA1*05:01/DQB1*02:01 | 233 | 247 | 15 | Consensus (comb.lib./smm/nn) | YANEIDYAHDLYDEL | 0.36 | 0.36 | -0.1128 |  |
| HLA-DQA1*01:01/DQB1*05:01 | 244 | 258 | 15 | Consensus (comb.lib./smm/nn) | YDELGWTDDVLPYMR | 0.22 | 0.22 | -0.1636 |  |

Rv2659c HTL epitope

| allele | start | end | length | method | peptide | percentile_rank | adjusted_rank | antigenicity score | IFN score |
| --- | --- | --- | --- | --- | --- | --- | --- | --- | --- |
| HLA-DRB1*01:01 | 198 | 212 | 15 | Consensus (comb.lib./smm/nn) | AFVLMAAWLAMRYGE | 0.16 | 0.16 | 0.7809 | 0.301353 |
| HLA-DRB1*01:01 | 199 | 213 | 15 | Consensus (comb.lib./smm/nn) | FVLMAAWLAMRYGEL | 0.16 | 0.16 | 0.7013 | 0.178036 |
| HLA-DRB1*01:01 | 196 | 210 | 15 | Consensus (comb.lib./smm/nn) | YQAFVLMAAWLAMRY | 0.01 | 0.01 | 0.6981 |  |
| HLA-DRB1*01:01 | 197 | 211 | 15 | Consensus (comb.lib./smm/nn) | QAFVLMAAWLAMRYG | 0.01 | 0.01 | 0.6379 |  |
| HLA-DRB1*01:01 | 195 | 209 | 15 | Consensus (comb.lib./smm/nn) | PYQAFVLMAAWLAMR | 0.01 | 0.01 | 0.5578 |  |
| HLA-DQA1*05:01/DQB1*03:01 | 317 | 331 | 15 | Consensus (comb.lib./smm/nn) | DLRHSGAVLAASTGA | 0.08 | 0.08 | 0.4936 |  |
| HLA-DQA1*01:02/DQB1*06:02 | 317 | 331 | 15 | Consensus (comb.lib./smm/nn) | DLRHSGAVLAASTGA | 0.18 | 0.18 | 0.4936 |  |
| HLA-DQA1*05:01/DQB1*03:01 | 320 | 334 | 15 | Consensus (comb.lib./smm/nn) | HSGAVLAASTGATLA | 0.39 | 0.39 | 0.4509 |  |
| HLA-DRB1*01:01 | 194 | 208 | 15 | Consensus (comb.lib./smm/nn) | DPYQAFVLMAAWLAM | 0.01 | 0.01 | 0.4091 |  |
| HLA-DQA1*05:01/DQB1*03:01 | 319 | 333 | 15 | Consensus (comb.lib./smm/nn) | RHSGAVLAASTGATL | 0.05 | 0.05 | 0.403 |  |
| HLA-DQA1*05:01/DQB1*03:01 | 318 | 332 | 15 | Consensus (comb.lib./smm/nn) | LRHSGAVLAASTGAT | 0.04 | 0.04 | 0.388 |  |
| HLA-DQA1*01:02/DQB1*06:02 | 318 | 332 | 15 | Consensus (comb.lib./smm/nn) | LRHSGAVLAASTGAT | 0.26 | 0.26 | 0.388 |  |
| HLA-DQA1*05:01/DQB1*03:01 | 314 | 328 | 15 | Consensus (comb.lib./smm/nn) | RVHDLRHSGAVLAAS | 0.19 | 0.19 | 0.3728 |  |
| HLA-DQA1*01:02/DQB1*06:02 | 314 | 328 | 15 | Consensus (comb.lib./smm/nn) | RVHDLRHSGAVLAAS | 0.22 | 0.22 | 0.3728 |  |
| HLA-DRB1*01:01 | 193 | 207 | 15 | Consensus (comb.lib./smm/nn) | PDPYQAFVLMAAWLA | 0.01 | 0.01 | 0.2805 |  |
| HLA-DQA1*05:01/DQB1*03:01 | 316 | 330 | 15 | Consensus (comb.lib./smm/nn) | HDLRHSGAVLAASTG | 0.08 | 0.08 | 0.2782 |  |
| HLA-DQA1*01:02/DQB1*06:02 | 316 | 330 | 15 | Consensus (comb.lib./smm/nn) | HDLRHSGAVLAASTG | 0.2 | 0.2 | 0.2782 |  |
| HLA-DRB5*01:01 | 298 | 312 | 15 | Consensus (smm/nn/sturniolo) | YRMFYKARKAAGRPD | 0.21 | 0.21 | 0.2387 |  |
| HLA-DRB1*11:01 | 298 | 312 | 15 | Consensus (smm/nn/sturniolo) | YRMFYKARKAAGRPD | 0.27 | 0.27 | 0.2387 |  |
| HLA-DRB5*01:01 | 296 | 310 | 15 | Consensus (smm/nn/sturniolo) | ALYRMFYKARKAAGR | 0.13 | 0.13 | 0.2041 |  |
| HLA-DRB1*11:01 | 296 | 310 | 15 | Consensus (smm/nn/sturniolo) | ALYRMFYKARKAAGR | 0.27 | 0.27 | 0.2041 |  |
| HLA-DRB5*01:01 | 299 | 313 | 15 | Consensus (smm/nn/sturniolo) | RMFYKARKAAGRPDL | 0.21 | 0.21 | 0.1784 |  |
| HLA-DRB5*01:01 | 300 | 314 | 15 | Consensus (smm/nn/sturniolo) | MFYKARKAAGRPDLR | 0.46 | 0.46 | 0.1732 |  |
| HLA-DRB5*01:01 | 297 | 311 | 15 | Consensus (smm/nn/sturniolo) | LYRMFYKARKAAGRP | 0.21 | 0.21 | 0.1124 |  |
| HLA-DRB1*11:01 | 297 | 311 | 15 | Consensus (smm/nn/sturniolo) | LYRMFYKARKAAGRP | 0.27 | 0.27 | 0.1124 |  |
| HLA-DQA1*05:01/DQB1*03:01 | 315 | 329 | 15 | Consensus (comb.lib./smm/nn) | VHDLRHSGAVLAAST | 0.08 | 0.08 | 0.0749 |  |
| HLA-DQA1*01:02/DQB1*06:02 | 315 | 329 | 15 | Consensus (comb.lib./smm/nn) | VHDLRHSGAVLAAST | 0.2 | 0.2 | 0.0749 |  |
| HLA-DRB1*11:01 | 295 | 309 | 15 | Consensus (smm/nn/sturniolo) | SALYRMFYKARKAAG | 0.27 | 0.27 | 0.0287 |  |
| HLA-DRB5*01:01 | 295 | 309 | 15 | Consensus (smm/nn/sturniolo) | SALYRMFYKARKAAG | 0.37 | 0.37 | 0.0287 |  |
| HLA-DRB1*11:01 | 294 | 308 | 15 | Consensus (smm/nn/sturniolo) | PSALYRMFYKARKAA | 0.27 | 0.27 | -0.0795 |  |
| HLA-DRB5*01:01 | 294 | 308 | 15 | Consensus (smm/nn/sturniolo) | PSALYRMFYKARKAA | 0.4 | 0.4 | -0.0795 |  |

Rv2660c HTL epitope

| allele | start | end | length | method | peptide | percentile_rank | adjusted_rank | antigenicity score |
| --- | --- | --- | --- | --- | --- | --- | --- | --- |
|  |  |  |  |  |  |  |  |  |

Rv3879c HTL epitope

| allele | start | end | length | method | peptide | percentile_rank | adjusted_rank | Antigenicity score | IFN score |
| --- | --- | --- | --- | --- | --- | --- | --- | --- | --- |
| HLA-DQA1*05:01/DQB1*03:01 | 381 | 395 | 15 | Consensus (comb.lib./smm/nn) | SGTAVGAGARSSVGT | 0.32 | 0.32 | 1.6513 | NEGATIVE |
| HLA-DQA1*05:01/DQB1*03:01 | 382 | 396 | 15 | Consensus (comb.lib./smm/nn) | GTAVGAGARSSVGTA | 0.34 | 0.34 | 1.5201 | NEGATIVE |
| HLA-DQA1*05:01/DQB1*03:01 | 380 | 394 | 15 | Consensus (comb.lib./smm/nn) | PSGTAVGAGARSSVG | 0.35 | 0.35 | 1.376 | NEGATIVE |
| HLA-DQA1*05:01/DQB1*03:01 | 378 | 392 | 15 | Consensus (comb.lib./smm/nn) | AAPSGTAVGAGARSS | 0.31 | 0.31 | 1.2542 | NEGATIVE |
| HLA-DQA1*05:01/DQB1*03:01 | 379 | 393 | 15 | Consensus (comb.lib./smm/nn) | APSGTAVGAGARSSV | 0.27 | 0.27 | 1.2012 | NEGATIVE |
| HLA-DQA1*05:01/DQB1*03:01 | 376 | 390 | 15 | Consensus (comb.lib./smm/nn) | AAAAPSGTAVGAGAR | 0.35 | 0.35 | 1.1352 | NEGATIVE |
| HLA-DQA1*05:01/DQB1*03:01 | 375 | 389 | 15 | Consensus (comb.lib./smm/nn) | AAAAAPSGTAVGAGA | 0.15 | 0.15 | 0.988 | NEGATIVE |
| HLA-DQA1*05:01/DQB1*03:01 | 365 | 379 | 15 | Consensus (comb.lib./smm/nn) | AAASGVPGARAAAAA | 0.09 | 0.09 | 0.9853 | 0.787876 |
| HLA-DQA1*05:01/DQB1*03:01 | 364 | 378 | 15 | Consensus (comb.lib./smm/nn) | PAAASGVPGARAAAA | 0.09 | 0.09 | 0.9405 | 0.649999 |
| HLA-DQA1*05:01/DQB1*03:01 | 374 | 388 | 15 | Consensus (comb.lib./smm/nn) | RAAAAAPSGTAVGAG | 0.13 | 0.13 | 0.9297 | NEGATIVE |
| HLA-DQA1*05:01/DQB1*03:01 | 367 | 381 | 15 | Consensus (comb.lib./smm/nn) | ASGVPGARAAAAAPS | 0.03 | 0.03 | 0.901 | 0.40506 |
| HLA-DQA1*05:01/DQB1*03:01 | 363 | 377 | 15 | Consensus (comb.lib./smm/nn) | TPAAASGVPGARAAA | 0.19 | 0.19 | 0.8836 | 0.445355 |
| HLA-DQA1*05:01/DQB1*03:01 | 366 | 380 | 15 | Consensus (comb.lib./smm/nn) | AASGVPGARAAAAAP | 0.03 | 0.03 | 0.8794 | 0.417119 |
| HLA-DQA1*05:01/DQB1*03:01 | 370 | 384 | 15 | Consensus (comb.lib./smm/nn) | VPGARAAAAAPSGTA | 0.09 | 0.09 | 0.8714 | NEGATIVE |
| HLA-DQA1*05:01/DQB1*03:01 | 372 | 386 | 15 | Consensus (comb.lib./smm/nn) | GARAAAAAPSGTAVG | 0.15 | 0.15 | 0.8694 | NEGATIVE |
| HLA-DQA1*05:01/DQB1*03:01 | 369 | 383 | 15 | Consensus (comb.lib./smm/nn) | GVPGARAAAAAPSGT | 0.09 | 0.09 | 0.852 | NEGATIVE |
| HLA-DQA1*05:01/DQB1*03:01 | 368 | 382 | 15 | Consensus (comb.lib./smm/nn) | SGVPGARAAAAAPSG | 0.03 | 0.03 | 0.7702 | NEGATIVE |
| HLA-DQA1*05:01/DQB1*03:01 | 373 | 387 | 15 | Consensus (comb.lib./smm/nn) | ARAAAAAPSGTAVGA | 0.09 | 0.09 | 0.7684 | NEGATIVE |
| HLA-DQA1*05:01/DQB1*03:01 | 371 | 385 | 15 | Consensus (comb.lib./smm/nn) | PGARAAAAAPSGTAV | 0.09 | 0.09 | 0.7456 | NEGATIVE |
| HLA-DPA1*02:01/DPB1*14:01 | 675 | 689 | 15 | NetMHCIIpan | AHLRAFRAYAAHSQE | 0.43 | 0.43 | 0.6861 |  |
| HLA-DPA1*02:01/DPB1*14:01 | 676 | 690 | 15 | NetMHCIIpan | HLRAFRAYAAHSQEI | 0.3 | 0.3 | 0.6815 |  |
| HLA-DPA1*02:01/DPB1*14:01 | 674 | 688 | 15 | NetMHCIIpan | AAHLRAFRAYAAHSQ | 0.41 | 0.41 | 0.6792 |  |
| HLA-DPA1*02:01/DPB1*14:01 | 490 | 504 | 15 | NetMHCIIpan | ARRIAAALNASDNNA | 0.12 | 0.12 | 0.5311 |  |
| HLA-DQA1*05:01/DQB1*03:01 | 58 | 72 | 15 | Consensus (comb.lib./smm/nn) | EGGLWSGGAANAANG | 0.25 | 0.25 | 0.4737 |  |
| HLA-DQA1*05:01/DQB1*03:01 | 59 | 73 | 15 | Consensus (comb.lib./smm/nn) | GGLWSGGAANAANGA | 0.31 | 0.31 | 0.4716 |  |
| HLA-DPA1*02:01/DPB1*14:01 | 677 | 691 | 15 | NetMHCIIpan | LRAFRAYAAHSQEIA | 0.34 | 0.34 | 0.4259 |  |
| HLA-DPA1*02:01/DPB1*14:01 | 489 | 503 | 15 | NetMHCIIpan | LARRIAAALNASDNN | 0.03 | 0.03 | 0.3765 |  |
| HLA-DPA1*02:01/DPB1*14:01 | 488 | 502 | 15 | NetMHCIIpan | RLARRIAAALNASDN | 0.01 | 0.01 | 0.3699 |  |
| HLA-DPA1*02:01/DPB1*14:01 | 484 | 498 | 15 | NetMHCIIpan | GDALRLARRIAAALN | 0.11 | 0.11 | 0.3522 |  |
| HLA-DPA1*02:01/DPB1*14:01 | 486 | 500 | 15 | NetMHCIIpan | ALRLARRIAAALNAS | 0.01 | 0.01 | 0.3486 |  |
| HLA-DPA1*02:01/DPB1*14:01 | 487 | 501 | 15 | NetMHCIIpan | LRLARRIAAALNASD | 0.01 | 0.01 | 0.3247 |  |
| HLA-DQA1*05:01/DQB1*03:01 | 60 | 74 | 15 | Consensus (comb.lib./smm/nn) | GLWSGGAANAANGAL | 0.37 | 0.37 | 0.3112 |  |
| HLA-DQA1*05:01/DQB1*03:01 | 57 | 71 | 15 | Consensus (comb.lib./smm/nn) | FEGGLWSGGAANAAN | 0.27 | 0.27 | 0.2476 |  |
| HLA-DPA1*02:01/DPB1*14:01 | 485 | 499 | 15 | NetMHCIIpan | DALRLARRIAAALNA | 0.01 | 0.01 | 0.1427 |  |
| HLA-DQA1*05:01/DQB1*02:01 | 15 | 29 | 15 | Consensus (comb.lib./smm/nn) | LDPGGWVEADEDTFY | 0.36 | 0.36 | -0.5236 |  |
| HLA-DQA1*05:01/DQB1*02:01 | 16 | 30 | 15 | Consensus (comb.lib./smm/nn) | DPGGWVEADEDTFYD | 0.29 | 0.29 | -0.6944 |  |
| HLA-DQA1*05:01/DQB1*02:01 | 17 | 31 | 15 | Consensus (comb.lib./smm/nn) | PGGWVEADEDTFYDR | 0.47 | 0.47 | -0.7016 |  |
