# Supplementary material for: In silico Analysis of Peptide-Based Biomarkers for the Diagnosis and Prevention of Latent Tuberculosis Infection
Source: Front Microbiol. 2022 Jun 28;13:947852. doi: 10.3389/fmicb.2022.947852 (PMC9273951; doi:10.3389/fmicb.2022.947852)
Supplement: Supplementary Figure 1 — The polypeptide molecular sequence was electronically cloned into the pET30a expression vector by SnapGene software. The diagnostic antigen is the gene coding for the insertion of antigen molecules, and the rest is the expression vector. [file Data_Sheet_1.zip › Supplementary Files/Table S2.docx]

Rv1737c CTL epitope

| allele | start | end | length | peptide | score | percentile_rank |
| --- | --- | --- | --- | --- | --- | --- |
| HLA-B*58:01 | 201 | 209 | 9 | AAARLPVTW | 0.995401 | 0.01 |
| HLA-B*57:01 | 201 | 209 | 9 | AAARLPVTW | 0.994148 | 0.01 |
| HLA-B*57:01 | 200 | 209 | 10 | KAAARLPVTW | 0.990272 | 0.01 |
| HLA-B*35:01 | 229 | 237 | 9 | LPTYITTIY | 0.988518 | 0.01 |
| HLA-B*58:01 | 200 | 209 | 10 | KAAARLPVTW | 0.981536 | 0.01 |
| HLA-B*58:01 | 5 | 13 | 9 | AANLVLATW | 0.979894 | 0.01 |
| HLA-B*58:01 | 289 | 298 | 10 | AALQPPPEVW | 0.977133 | 0.01 |
| HLA-A*24:02 | 231 | 239 | 9 | TYITTIYGF | 0.976293 | 0.01 |
| HLA-A*23:01 | 231 | 239 | 9 | TYITTIYGF | 0.975753 | 0.01 |
| HLA-A*01:01 | 354 | 363 | 10 | ATYDPVDNDY | 0.96262 | 0.01 |
| HLA-A*68:02 | 234 | 242 | 9 | TTIYGFSTV | 0.943923 | 0.01 |
| HLA-A*31:01 | 181 | 189 | 9 | VLRDAPYFR | 0.937235 | 0.01 |
| HLA-B*57:01 | 5 | 13 | 9 | AANLVLATW | 0.985319 | 0.02 |
| HLA-B*07:02 | 270 | 279 | 10 | APRHVVLASL | 0.975829 | 0.02 |
| HLA-B*35:01 | 205 | 213 | 9 | LPVTWEMSF | 0.966879 | 0.02 |
| HLA-A*31:01 | 148 | 156 | 9 | AFFTPRFVR | 0.90662 | 0.02 |
| HLA-A*32:01 | 217 | 225 | 9 | IVFGGFVAF | 0.781107 | 0.02 |
| HLA-B*57:01 | 289 | 298 | 10 | AALQPPPEVW | 0.978364 | 0.03 |
| HLA-A*02:01 | 228 | 236 | 9 | YLPTYITTI | 0.931305 | 0.03 |
| HLA-A*02:03 | 228 | 236 | 9 | YLPTYITTI | 0.919848 | 0.03 |
| HLA-B*15:01 | 24 | 32 | 9 | LIGPLSTSY | 0.868073 | 0.03 |
| HLA-B*53:01 | 205 | 213 | 9 | LPVTWEMSF | 0.863269 | 0.03 |
| HLA-B*08:01 | 268 | 276 | 9 | RIAPRHVVL | 0.836175 | 0.03 |
| HLA-A*32:01 | 290 | 298 | 9 | ALQPPPEVW | 0.75562 | 0.03 |
| HLA-B*57:01 | 11 | 20 | 10 | ATWISVVNFW | 0.967517 | 0.04 |
| HLA-A*02:06 | 108 | 116 | 9 | GVAGTIFAV | 0.892739 | 0.04 |
| HLA-A*02:01 | 78 | 86 | 9 | TLASILPVL | 0.883691 | 0.04 |
| HLA-A*02:03 | 9 | 17 | 9 | VLATWISVV | 0.8525 | 0.04 |
| HLA-B*15:01 | 217 | 225 | 9 | IVFGGFVAF | 0.852009 | 0.04 |
| HLA-B*53:01 | 229 | 237 | 9 | LPTYITTIY | 0.775055 | 0.04 |
| HLA-B*58:01 | 4 | 13 | 10 | QAANLVLATW | 0.925822 | 0.05 |
| HLA-A*02:03 | 78 | 86 | 9 | TLASILPVL | 0.822079 | 0.05 |
| HLA-A*23:01 | 149 | 157 | 9 | FFTPRFVRW | 0.766018 | 0.05 |
| HLA-A*30:02 | 207 | 215 | 9 | VTWEMSFLY | 0.717927 | 0.05 |
| HLA-A*30:02 | 354 | 363 | 10 | ATYDPVDNDY | 0.715288 | 0.05 |
| HLA-A*30:02 | 338 | 346 | 9 | AAAGGLGGY | 0.709809 | 0.05 |
| HLA-A*32:01 | 201 | 209 | 9 | AAARLPVTW | 0.669329 | 0.05 |
| HLA-A*32:01 | 268 | 276 | 9 | RIAPRHVVL | 0.65371 | 0.05 |
| HLA-B*57:01 | 14 | 22 | 9 | ISVVNFWAW | 0.955523 | 0.06 |
| HLA-B*57:01 | 4 | 13 | 10 | QAANLVLATW | 0.955445 | 0.06 |
| HLA-B*58:01 | 116 | 124 | 9 | VGIPFANNW | 0.913184 | 0.06 |
| HLA-B*58:01 | 14 | 22 | 9 | ISVVNFWAW | 0.910796 | 0.06 |
| HLA-A*02:06 | 228 | 236 | 9 | YLPTYITTI | 0.851478 | 0.06 |
| HLA-B*07:02 | 196 | 205 | 10 | LPRLKAAARL | 0.849409 | 0.06 |
| HLA-B*35:01 | 347 | 356 | 10 | FPPLVMGATY | 0.843716 | 0.06 |
| HLA-A*31:01 | 316 | 324 | 9 | GVFAWVARR | 0.822447 | 0.06 |
| HLA-A*31:01 | 180 | 189 | 10 | VVLRDAPYFR | 0.812505 | 0.06 |
| HLA-B*15:01 | 23 | 32 | 10 | NLIGPLSTSY | 0.805336 | 0.06 |
| HLA-A*23:01 | 12 | 20 | 9 | TWISVVNFW | 0.758442 | 0.06 |
| HLA-A*33:01 | 148 | 156 | 9 | AFFTPRFVR | 0.721763 | 0.06 |
| HLA-B*53:01 | 201 | 209 | 9 | AAARLPVTW | 0.710113 | 0.06 |
| HLA-A*30:02 | 224 | 232 | 9 | AFSNYLPTY | 0.686425 | 0.06 |
| HLA-A*30:02 | 24 | 32 | 9 | LIGPLSTSY | 0.685572 | 0.06 |
| HLA-B*07:02 | 268 | 276 | 9 | RIAPRHVVL | 0.826349 | 0.07 |
| HLA-A*68:02 | 162 | 170 | 9 | TTHAIVAAA | 0.749154 | 0.07 |
| HLA-A*68:02 | 296 | 304 | 9 | EVWSAATFI | 0.737563 | 0.07 |
| HLA-A*33:01 | 181 | 189 | 9 | VLRDAPYFR | 0.702236 | 0.07 |
| HLA-A*30:02 | 89 | 97 | 9 | GVAATMGSY | 0.665027 | 0.07 |
| HLA-A*26:01 | 23 | 32 | 10 | NLIGPLSTSY | 0.65261 | 0.07 |
| HLA-B*53:01 | 5 | 13 | 9 | AANLVLATW | 0.635562 | 0.07 |
| HLA-A*26:01 | 89 | 97 | 9 | GVAATMGSY | 0.626201 | 0.07 |
| HLA-A*32:01 | 11 | 19 | 9 | ATWISVVNF | 0.579924 | 0.07 |
| HLA-B*57:01 | 116 | 124 | 9 | VGIPFANNW | 0.929719 | 0.08 |
| HLA-B*58:01 | 290 | 298 | 9 | ALQPPPEVW | 0.868966 | 0.08 |
| HLA-B*07:02 | 325 | 333 | 9 | APAASVGSV | 0.812005 | 0.08 |
| HLA-B*35:01 | 228 | 237 | 10 | YLPTYITTIY | 0.806038 | 0.08 |
| HLA-B*35:01 | 171 | 179 | 9 | LASTAVVAM | 0.795631 | 0.08 |
| HLA-A*02:06 | 78 | 86 | 9 | TLASILPVL | 0.788119 | 0.08 |
| HLA-A*31:01 | 377 | 386 | 10 | ACTYTALHAR | 0.759665 | 0.08 |
| HLA-A*31:01 | 378 | 386 | 9 | CTYTALHAR | 0.754729 | 0.08 |
| HLA-A*24:02 | 149 | 157 | 9 | FFTPRFVRW | 0.73921 | 0.08 |
| HLA-A*68:02 | 363 | 371 | 9 | YTVGLLLLV | 0.719621 | 0.08 |
| HLA-A*30:02 | 372 | 380 | 9 | ATALVACTY | 0.643313 | 0.08 |
| HLA-A*01:01 | 207 | 215 | 9 | VTWEMSFLY | 0.728352 | 0.09 |
| HLA-A*02:03 | 108 | 116 | 9 | GVAGTIFAV | 0.723589 | 0.09 |
| HLA-A*24:02 | 227 | 236 | 10 | NYLPTYITTI | 0.714835 | 0.09 |
| HLA-A*24:02 | 12 | 20 | 9 | TWISVVNFW | 0.71032 | 0.09 |
| HLA-A*33:01 | 378 | 386 | 9 | CTYTALHAR | 0.63778 | 0.09 |
| HLA-A*32:01 | 5 | 13 | 9 | AANLVLATW | 0.527368 | 0.09 |
| HLA-B*58:01 | 11 | 20 | 10 | ATWISVVNFW | 0.827638 | 0.1 |
| HLA-B*58:01 | 312 | 320 | 9 | VGTGGVFAW | 0.826922 | 0.1 |
| HLA-A*02:01 | 108 | 116 | 9 | GVAGTIFAV | 0.757326 | 0.1 |
| HLA-A*02:06 | 289 | 297 | 9 | AALQPPPEV | 0.737745 | 0.1 |
| HLA-A*31:01 | 147 | 156 | 10 | SAFFTPRFVR | 0.737472 | 0.1 |
| HLA-A*01:01 | 372 | 380 | 9 | ATALVACTY | 0.675969 | 0.1 |
| HLA-B*51:01 | 147 | 155 | 9 | SAFFTPRFV | 0.644711 | 0.1 |
| HLA-A*23:01 | 153 | 161 | 9 | RFVRWFGLF | 0.631642 | 0.1 |
| HLA-A*33:01 | 147 | 156 | 10 | SAFFTPRFVR | 0.624504 | 0.1 |
| HLA-A*30:01 | 268 | 276 | 9 | RIAPRHVVL | 0.555264 | 0.1 |
| HLA-A*32:01 | 207 | 215 | 9 | VTWEMSFLY | 0.503702 | 0.1 |
| HLA-A*31:01 | 145 | 153 | 9 | ALSAFFTPR | 0.732367 | 0.11 |
| HLA-A*68:02 | 173 | 181 | 9 | STAVVAMVV | 0.643982 | 0.11 |
| HLA-A*23:01 | 227 | 236 | 10 | NYLPTYITTI | 0.618899 | 0.11 |
| HLA-A*68:01 | 378 | 386 | 9 | CTYTALHAR | 0.865434 | 0.12 |
| HLA-B*35:01 | 217 | 225 | 9 | IVFGGFVAF | 0.699373 | 0.12 |
| HLA-A*02:06 | 363 | 371 | 9 | YTVGLLLLV | 0.683383 | 0.12 |
| HLA-A*23:01 | 214 | 222 | 9 | LYAIVFGGF | 0.575048 | 0.12 |
| HLA-A*26:01 | 338 | 346 | 9 | AAAGGLGGY | 0.498764 | 0.12 |
| HLA-A*32:01 | 200 | 209 | 10 | KAAARLPVTW | 0.46134 | 0.12 |
| HLA-A*31:01 | 175 | 183 | 9 | AVVAMVVLR | 0.70443 | 0.13 |
| HLA-B*35:01 | 86 | 94 | 9 | LAVGVAATM | 0.698431 | 0.13 |
| HLA-A*02:01 | 9 | 17 | 9 | VLATWISVV | 0.69631 | 0.13 |
| HLA-A*68:02 | 332 | 340 | 9 | SVTGIVAAA | 0.583936 | 0.13 |
| HLA-A*68:01 | 175 | 183 | 9 | AVVAMVVLR | 0.861067 | 0.14 |
| HLA-B*58:01 | 60 | 68 | 9 | VTGPLTDRF | 0.773947 | 0.14 |
| HLA-B*51:01 | 191 | 199 | 9 | NADPVLPRL | 0.571223 | 0.14 |
| HLA-A*33:01 | 264 | 272 | 9 | WLSDRIAPR | 0.539644 | 0.14 |
| HLA-B*53:01 | 4 | 13 | 10 | QAANLVLATW | 0.484373 | 0.14 |
| HLA-A*26:01 | 354 | 363 | 10 | ATYDPVDNDY | 0.451786 | 0.14 |
| HLA-A*26:01 | 217 | 225 | 9 | IVFGGFVAF | 0.447846 | 0.14 |
| HLA-A*32:01 | 71 | 79 | 9 | RAMLIAVTL | 0.442652 | 0.14 |
| HLA-B*35:01 | 24 | 32 | 9 | LIGPLSTSY | 0.637706 | 0.15 |
| HLA-A*02:06 | 191 | 199 | 9 | NADPVLPRL | 0.622099 | 0.15 |
| HLA-A*24:02 | 124 | 132 | 9 | WYQPARRGF | 0.591858 | 0.15 |
| HLA-A*68:02 | 108 | 116 | 9 | GVAGTIFAV | 0.559097 | 0.15 |
| HLA-A*23:01 | 124 | 132 | 9 | WYQPARRGF | 0.523896 | 0.15 |
| HLA-A*26:01 | 179 | 187 | 9 | MVVLRDAPY | 0.423236 | 0.15 |
| HLA-B*57:01 | 115 | 124 | 10 | AVGIPFANNW | 0.852635 | 0.16 |
| HLA-A*24:02 | 214 | 222 | 9 | LYAIVFGGF | 0.563251 | 0.16 |
| HLA-B*51:01 | 205 | 213 | 9 | LPVTWEMSF | 0.54268 | 0.16 |
| HLA-B*08:01 | 30 | 38 | 9 | TSYARDMSL | 0.479051 | 0.16 |
| HLA-B*53:01 | 347 | 356 | 10 | FPPLVMGATY | 0.452999 | 0.16 |
| HLA-A*32:01 | 256 | 264 | 9 | VLARPVGGW | 0.394249 | 0.16 |
| HLA-A*32:01 | 115 | 124 | 10 | AVGIPFANNW | 0.383257 | 0.16 |
| HLA-B*57:01 | 290 | 298 | 9 | ALQPPPEVW | 0.840434 | 0.17 |
| HLA-A*23:01 | 230 | 239 | 10 | PTYITTIYGF | 0.484881 | 0.17 |
| HLA-A*23:01 | 148 | 157 | 10 | AFFTPRFVRW | 0.482921 | 0.17 |
| HLA-A*32:01 | 216 | 225 | 10 | AIVFGGFVAF | 0.377086 | 0.17 |
| HLA-B*15:01 | 89 | 97 | 9 | GVAATMGSY | 0.650548 | 0.18 |
| HLA-A*24:02 | 153 | 161 | 9 | RFVRWFGLF | 0.540529 | 0.18 |
| HLA-B*53:01 | 289 | 298 | 10 | AALQPPPEVW | 0.415183 | 0.18 |
| HLA-B*58:01 | 207 | 215 | 9 | VTWEMSFLY | 0.702963 | 0.19 |
| HLA-B*58:01 | 146 | 154 | 9 | LSAFFTPRF | 0.701514 | 0.19 |
| HLA-A*68:02 | 39 | 47 | 9 | SSAEASLLV | 0.509085 | 0.19 |
| HLA-B*51:01 | 357 | 365 | 9 | DPVDNDYTV | 0.495572 | 0.19 |
| HLA-B*57:01 | 312 | 320 | 9 | VGTGGVFAW | 0.811638 | 0.2 |
| HLA-B*15:01 | 216 | 225 | 10 | AIVFGGFVAF | 0.617305 | 0.2 |
| HLA-A*02:01 | 45 | 53 | 9 | LLVATPILV | 0.600455 | 0.2 |
| HLA-A*02:06 | 234 | 242 | 9 | TTIYGFSTV | 0.549189 | 0.2 |
| HLA-B*51:01 | 289 | 297 | 9 | AALQPPPEV | 0.491833 | 0.2 |
| HLA-A*33:01 | 122 | 130 | 9 | NNWYQPARR | 0.466879 | 0.2 |
| HLA-B*08:01 | 267 | 276 | 10 | DRIAPRHVVL | 0.426834 | 0.2 |
| HLA-B*57:01 | 146 | 154 | 9 | LSAFFTPRF | 0.792469 | 0.21 |
| HLA-B*58:01 | 115 | 124 | 10 | AVGIPFANNW | 0.657338 | 0.21 |
| HLA-B*07:02 | 270 | 278 | 9 | APRHVVLAS | 0.550411 | 0.21 |
| HLA-A*02:03 | 252 | 261 | 10 | ALAAVLARPV | 0.534209 | 0.21 |
| HLA-A*68:02 | 161 | 169 | 9 | FTTHAIVAA | 0.484916 | 0.21 |
| HLA-A*33:01 | 180 | 189 | 10 | VVLRDAPYFR | 0.452007 | 0.21 |
| HLA-B*53:01 | 191 | 199 | 9 | NADPVLPRL | 0.379179 | 0.21 |
| HLA-B*57:01 | 207 | 215 | 9 | VTWEMSFLY | 0.783227 | 0.22 |
| HLA-A*68:01 | 316 | 324 | 9 | GVFAWVARR | 0.780287 | 0.22 |
| HLA-A*11:01 | 175 | 183 | 9 | AVVAMVVLR | 0.58442 | 0.22 |
| HLA-A*02:01 | 342 | 350 | 9 | GLGGYFPPL | 0.568473 | 0.22 |
| HLA-B*07:02 | 245 | 253 | 9 | GARTAGFAL | 0.521413 | 0.22 |
| HLA-B*08:01 | 196 | 205 | 10 | LPRLKAAARL | 0.410514 | 0.22 |
| HLA-A*11:01 | 377 | 386 | 10 | ACTYTALHAR | 0.567311 | 0.23 |
| HLA-A*02:03 | 45 | 53 | 9 | LLVATPILV | 0.515315 | 0.23 |
| HLA-B*35:01 | 179 | 187 | 9 | MVVLRDAPY | 0.496021 | 0.23 |
| HLA-A*24:02 | 230 | 239 | 10 | PTYITTIYGF | 0.446679 | 0.23 |
| HLA-A*24:02 | 362 | 370 | 9 | DYTVGLLLL | 0.443818 | 0.23 |
| HLA-B*53:01 | 116 | 124 | 9 | VGIPFANNW | 0.357715 | 0.23 |
| HLA-A*32:01 | 289 | 298 | 10 | AALQPPPEVW | 0.320191 | 0.23 |
| HLA-A*02:01 | 37 | 45 | 9 | SLSSAEASL | 0.531308 | 0.24 |
| HLA-A*68:02 | 147 | 155 | 9 | SAFFTPRFV | 0.437266 | 0.24 |
| HLA-A*30:02 | 223 | 232 | 10 | VAFSNYLPTY | 0.430391 | 0.24 |
| HLA-A*23:01 | 217 | 225 | 9 | IVFGGFVAF | 0.370554 | 0.24 |
| HLA-B*53:01 | 228 | 237 | 10 | YLPTYITTIY | 0.343243 | 0.24 |
| HLA-A*26:01 | 372 | 380 | 9 | ATALVACTY | 0.307372 | 0.24 |
| HLA-A*11:01 | 316 | 324 | 9 | GVFAWVARR | 0.548351 | 0.25 |
| HLA-B*15:01 | 372 | 380 | 9 | ATALVACTY | 0.538072 | 0.25 |
| HLA-A*02:06 | 81 | 90 | 10 | SILPVLAVGV | 0.49899 | 0.25 |
| HLA-B*07:02 | 151 | 160 | 10 | TPRFVRWFGL | 0.486436 | 0.25 |
| HLA-A*33:01 | 316 | 324 | 9 | GVFAWVARR | 0.423294 | 0.25 |
| HLA-A*01:01 | 24 | 32 | 9 | LIGPLSTSY | 0.407463 | 0.26 |
| HLA-A*32:01 | 255 | 264 | 10 | AVLARPVGGW | 0.290281 | 0.26 |
| HLA-A*32:01 | 92 | 100 | 9 | ATMGSYALL | 0.288208 | 0.26 |
| HLA-B*57:01 | 11 | 19 | 9 | ATWISVVNF | 0.7327 | 0.27 |
| HLA-B*40:01 | 209 | 217 | 9 | WEMSFLYAI | 0.501438 | 0.27 |
| HLA-A*02:01 | 44 | 52 | 9 | SLLVATPIL | 0.484156 | 0.27 |
| HLA-A*30:02 | 220 | 228 | 9 | GGFVAFSNY | 0.396516 | 0.27 |
| HLA-A*30:02 | 23 | 32 | 10 | NLIGPLSTSY | 0.39371 | 0.27 |
| HLA-A*30:02 | 355 | 363 | 9 | TYDPVDNDY | 0.392507 | 0.27 |
| HLA-A*23:01 | 290 | 298 | 9 | ALQPPPEVW | 0.32919 | 0.27 |
| HLA-A*23:01 | 149 | 158 | 10 | FFTPRFVRWF | 0.32309 | 0.27 |
| HLA-A*32:01 | 11 | 20 | 10 | ATWISVVNFW | 0.276343 | 0.27 |
| HLA-A*68:01 | 147 | 156 | 10 | SAFFTPRFVR | 0.724681 | 0.28 |
| HLA-B*58:01 | 180 | 188 | 9 | VVLRDAPYF | 0.554452 | 0.28 |
| HLA-A*02:03 | 37 | 45 | 9 | SLSSAEASL | 0.464178 | 0.28 |
| HLA-B*53:01 | 290 | 298 | 9 | ALQPPPEVW | 0.309236 | 0.28 |
| HLA-A*03:01 | 316 | 324 | 9 | GVFAWVARR | 0.549401 | 0.29 |
| HLA-A*02:01 | 283 | 291 | 9 | ALLAFAAAL | 0.465562 | 0.29 |
| HLA-B*07:02 | 324 | 333 | 10 | RAPAASVGSV | 0.458149 | 0.29 |
| HLA-A*68:02 | 92 | 100 | 9 | ATMGSYALL | 0.381718 | 0.29 |
| HLA-A*68:02 | 191 | 199 | 9 | NADPVLPRL | 0.381192 | 0.29 |
| HLA-A*24:02 | 148 | 157 | 10 | AFFTPRFVRW | 0.356618 | 0.29 |
| HLA-A*23:01 | 362 | 370 | 9 | DYTVGLLLL | 0.301948 | 0.29 |
| HLA-A*32:01 | 204 | 213 | 10 | RLPVTWEMSF | 0.265891 | 0.29 |
| HLA-A*68:01 | 174 | 183 | 10 | TAVVAMVVLR | 0.716891 | 0.3 |
| HLA-A*68:01 | 377 | 386 | 10 | ACTYTALHAR | 0.710028 | 0.3 |
| HLA-B*15:01 | 140 | 149 | 10 | GMVGTALSAF | 0.499173 | 0.3 |
| HLA-B*35:01 | 223 | 232 | 10 | VAFSNYLPTY | 0.424938 | 0.3 |
| HLA-A*26:01 | 234 | 242 | 9 | TTIYGFSTV | 0.247467 | 0.3 |
| HLA-B*57:01 | 60 | 68 | 9 | VTGPLTDRF | 0.694513 | 0.31 |
| HLA-B*15:01 | 278 | 287 | 10 | SLAGTALLAF | 0.491167 | 0.31 |
| HLA-A*02:06 | 9 | 17 | 9 | VLATWISVV | 0.455626 | 0.31 |
| HLA-A*26:01 | 207 | 215 | 9 | VTWEMSFLY | 0.243553 | 0.31 |
| HLA-A*68:02 | 299 | 307 | 9 | SAATFITLA | 0.364761 | 0.32 |
| HLA-A*01:01 | 355 | 363 | 9 | TYDPVDNDY | 0.344418 | 0.32 |
| HLA-A*24:02 | 290 | 298 | 9 | ALQPPPEVW | 0.323577 | 0.32 |
| HLA-A*23:01 | 123 | 132 | 10 | NWYQPARRGF | 0.273622 | 0.32 |
| HLA-A*32:01 | 180 | 188 | 9 | VVLRDAPYF | 0.245245 | 0.32 |
| HLA-B*58:01 | 11 | 19 | 9 | ATWISVVNF | 0.488262 | 0.33 |
| HLA-B*15:01 | 338 | 346 | 9 | AAAGGLGGY | 0.470263 | 0.33 |
| HLA-A*68:02 | 362 | 371 | 10 | DYTVGLLLLV | 0.356281 | 0.33 |
| HLA-A*32:01 | 112 | 120 | 9 | TIFAVGIPF | 0.229429 | 0.33 |
| HLA-A*32:01 | 95 | 103 | 9 | GSYALLVFF | 0.226134 | 0.33 |
| HLA-B*15:01 | 141 | 149 | 9 | MVGTALSAF | 0.46381 | 0.34 |
| HLA-A*11:01 | 354 | 363 | 10 | ATYDPVDNDY | 0.462094 | 0.34 |
| HLA-A*02:06 | 80 | 88 | 9 | ASILPVLAV | 0.430392 | 0.34 |
| HLA-B*35:01 | 191 | 199 | 9 | NADPVLPRL | 0.376272 | 0.34 |
| HLA-A*68:02 | 225 | 233 | 9 | FSNYLPTYI | 0.347384 | 0.34 |
| HLA-A*24:02 | 149 | 158 | 10 | FFTPRFVRWF | 0.303182 | 0.34 |
| HLA-A*32:01 | 146 | 154 | 9 | LSAFFTPRF | 0.223431 | 0.34 |
| HLA-B*57:01 | 95 | 103 | 9 | GSYALLVFF | 0.64922 | 0.35 |
| HLA-B*58:01 | 95 | 103 | 9 | GSYALLVFF | 0.463595 | 0.35 |
| HLA-B*15:01 | 354 | 363 | 10 | ATYDPVDNDY | 0.45891 | 0.35 |
| HLA-A*11:01 | 207 | 215 | 9 | VTWEMSFLY | 0.458533 | 0.35 |
| HLA-A*02:01 | 289 | 297 | 9 | AALQPPPEV | 0.414073 | 0.35 |
| HLA-A*02:03 | 159 | 167 | 9 | GLFTTHAIV | 0.39042 | 0.35 |
| HLA-A*24:02 | 123 | 132 | 10 | NWYQPARRGF | 0.296853 | 0.35 |
| HLA-B*35:01 | 279 | 287 | 9 | LAGTALLAF | 0.356009 | 0.36 |
| HLA-A*30:02 | 337 | 346 | 10 | VAAAGGLGGY | 0.333388 | 0.36 |
| HLA-A*33:01 | 251 | 259 | 9 | FALAAVLAR | 0.33106 | 0.36 |
| HLA-A*32:01 | 16 | 24 | 9 | VVNFWAWNL | 0.21193 | 0.36 |
| HLA-A*26:01 | 24 | 32 | 9 | LIGPLSTSY | 0.20925 | 0.36 |
| HLA-A*31:01 | 156 | 164 | 9 | RWFGLFTTH | 0.495421 | 0.37 |
| HLA-B*35:01 | 348 | 356 | 9 | PPLVMGATY | 0.344883 | 0.37 |
| HLA-A*30:02 | 156 | 164 | 9 | RWFGLFTTH | 0.325194 | 0.37 |
| HLA-A*68:01 | 180 | 189 | 10 | VVLRDAPYFR | 0.64982 | 0.38 |
| HLA-A*11:01 | 378 | 386 | 9 | CTYTALHAR | 0.430894 | 0.38 |
| HLA-A*30:01 | 247 | 255 | 9 | RTAGFALAA | 0.318571 | 0.38 |
| HLA-A*26:01 | 228 | 237 | 10 | YLPTYITTIY | 0.196987 | 0.38 |
| HLA-A*02:01 | 82 | 90 | 9 | ILPVLAVGV | 0.3841 | 0.39 |
| HLA-B*07:02 | 126 | 134 | 9 | QPARRGFST | 0.36125 | 0.39 |
| HLA-B*35:01 | 141 | 149 | 9 | MVGTALSAF | 0.334491 | 0.39 |
| HLA-A*23:01 | 105 | 114 | 10 | LFLGVAGTIF | 0.215807 | 0.39 |
| HLA-A*26:01 | 141 | 149 | 9 | MVGTALSAF | 0.191477 | 0.39 |
| HLA-B*57:01 | 255 | 264 | 10 | AVLARPVGGW | 0.602822 | 0.4 |
| HLA-A*03:01 | 145 | 153 | 9 | ALSAFFTPR | 0.459875 | 0.4 |
| HLA-B*58:01 | 256 | 264 | 9 | VLARPVGGW | 0.410305 | 0.4 |
| HLA-A*02:06 | 77 | 85 | 9 | VTLASILPV | 0.392782 | 0.4 |
| HLA-A*32:01 | 372 | 380 | 9 | ATALVACTY | 0.189502 | 0.4 |
| HLA-B*58:01 | 149 | 157 | 9 | FFTPRFVRW | 0.399257 | 0.41 |
| HLA-A*02:01 | 81 | 90 | 10 | SILPVLAVGV | 0.3653 | 0.41 |
| HLA-B*35:01 | 207 | 215 | 9 | VTWEMSFLY | 0.314411 | 0.41 |
| HLA-A*01:01 | 338 | 346 | 9 | AAAGGLGGY | 0.276918 | 0.41 |
| HLA-B*53:01 | 312 | 320 | 9 | VGTGGVFAW | 0.210245 | 0.41 |
| HLA-B*53:01 | 149 | 157 | 9 | FFTPRFVRW | 0.20638 | 0.41 |
| HLA-B*53:01 | 200 | 209 | 10 | KAAARLPVTW | 0.206367 | 0.41 |
| HLA-A*68:01 | 59 | 67 | 9 | IVTGPLTDR | 0.62665 | 0.42 |
| HLA-B*57:01 | 180 | 188 | 9 | VVLRDAPYF | 0.584043 | 0.42 |
| HLA-B*15:01 | 228 | 237 | 10 | YLPTYITTIY | 0.408572 | 0.42 |
| HLA-A*68:02 | 172 | 180 | 9 | ASTAVVAMV | 0.296406 | 0.42 |
| HLA-A*68:02 | 47 | 55 | 9 | VATPILVGA | 0.294702 | 0.42 |
| HLA-A*32:01 | 116 | 124 | 9 | VGIPFANNW | 0.185208 | 0.42 |
| HLA-A*31:01 | 58 | 67 | 10 | RIVTGPLTDR | 0.456443 | 0.43 |
| HLA-B*58:01 | 211 | 219 | 9 | MSFLYAIVF | 0.38045 | 0.43 |
| HLA-B*58:01 | 372 | 380 | 9 | ATALVACTY | 0.378168 | 0.43 |
| HLA-B*51:01 | 158 | 166 | 9 | FGLFTTHAI | 0.326924 | 0.43 |
| HLA-B*44:02 | 290 | 298 | 9 | ALQPPPEVW | 0.238274 | 0.43 |
| HLA-A*24:02 | 228 | 236 | 9 | YLPTYITTI | 0.228613 | 0.43 |
| HLA-A*23:01 | 11 | 20 | 10 | ATWISVVNFW | 0.195601 | 0.43 |
| HLA-B*15:01 | 112 | 120 | 9 | TIFAVGIPF | 0.391935 | 0.44 |
| HLA-B*58:01 | 71 | 79 | 9 | RAMLIAVTL | 0.364205 | 0.44 |
| HLA-A*02:01 | 44 | 53 | 10 | SLLVATPILV | 0.352894 | 0.44 |
| HLA-A*30:02 | 88 | 97 | 10 | VGVAATMGSY | 0.300432 | 0.44 |
| HLA-B*35:01 | 112 | 120 | 9 | TIFAVGIPF | 0.286192 | 0.44 |
| HLA-B*53:01 | 14 | 22 | 9 | ISVVNFWAW | 0.196375 | 0.44 |
| HLA-A*32:01 | 14 | 22 | 9 | ISVVNFWAW | 0.17387 | 0.44 |
| HLA-A*68:01 | 251 | 259 | 9 | FALAAVLAR | 0.608863 | 0.45 |
| HLA-B*58:01 | 201 | 210 | 10 | AAARLPVTWE | 0.362404 | 0.45 |
| HLA-A*02:01 | 363 | 371 | 9 | YTVGLLLLV | 0.348257 | 0.45 |
| HLA-A*02:03 | 82 | 90 | 9 | ILPVLAVGV | 0.328723 | 0.45 |
| HLA-A*02:03 | 51 | 59 | 9 | ILVGALGRI | 0.327446 | 0.45 |
| HLA-B*07:02 | 205 | 213 | 9 | LPVTWEMSF | 0.31697 | 0.45 |
| HLA-A*30:01 | 148 | 156 | 9 | AFFTPRFVR | 0.291021 | 0.45 |
| HLA-A*24:02 | 67 | 75 | 9 | RFGGRAMLI | 0.222926 | 0.45 |
| HLA-A*32:01 | 60 | 68 | 9 | VTGPLTDRF | 0.166515 | 0.45 |
| HLA-A*26:01 | 88 | 97 | 10 | VGVAATMGSY | 0.161544 | 0.45 |
| HLA-A*03:01 | 58 | 67 | 10 | RIVTGPLTDR | 0.413391 | 0.46 |
| HLA-A*02:03 | 278 | 286 | 9 | SLAGTALLA | 0.323725 | 0.46 |
| HLA-A*68:02 | 80 | 88 | 9 | ASILPVLAV | 0.281796 | 0.46 |
| HLA-B*53:01 | 348 | 356 | 9 | PPLVMGATY | 0.185414 | 0.46 |
| HLA-A*32:01 | 148 | 157 | 10 | AFFTPRFVRW | 0.162384 | 0.46 |
| HLA-B*57:01 | 256 | 264 | 9 | VLARPVGGW | 0.550896 | 0.47 |
| HLA-B*15:01 | 290 | 298 | 9 | ALQPPPEVW | 0.373477 | 0.47 |
| HLA-A*02:06 | 332 | 340 | 9 | SVTGIVAAA | 0.351612 | 0.47 |
| HLA-A*02:06 | 3 | 11 | 9 | GQAANLVLA | 0.348596 | 0.47 |
| HLA-A*02:01 | 93 | 101 | 9 | TMGSYALLV | 0.341144 | 0.47 |
| HLA-A*02:03 | 268 | 276 | 9 | RIAPRHVVL | 0.315553 | 0.47 |
| HLA-B*07:02 | 71 | 79 | 9 | RAMLIAVTL | 0.295693 | 0.47 |
| HLA-A*68:02 | 299 | 308 | 10 | SAATFITLAV | 0.27534 | 0.47 |
| HLA-A*01:01 | 265 | 273 | 9 | LSDRIAPRH | 0.240098 | 0.47 |
| HLA-A*23:01 | 180 | 188 | 9 | VVLRDAPYF | 0.180435 | 0.47 |
| HLA-A*26:01 | 216 | 225 | 10 | AIVFGGFVAF | 0.158549 | 0.47 |
| HLA-A*03:01 | 181 | 189 | 9 | VLRDAPYFR | 0.402423 | 0.48 |
| HLA-B*15:01 | 179 | 187 | 9 | MVVLRDAPY | 0.365141 | 0.48 |
| HLA-A*02:06 | 45 | 53 | 9 | LLVATPILV | 0.345213 | 0.48 |
| HLA-A*02:01 | 342 | 351 | 10 | GLGGYFPPLV | 0.334209 | 0.48 |
| HLA-A*02:03 | 170 | 178 | 9 | ALASTAVVA | 0.313661 | 0.48 |
| HLA-B*51:01 | 356 | 365 | 10 | YDPVDNDYTV | 0.290384 | 0.48 |
| HLA-A*68:02 | 162 | 171 | 10 | TTHAIVAAAL | 0.271645 | 0.48 |
| HLA-B*35:01 | 23 | 32 | 10 | NLIGPLSTSY | 0.263454 | 0.48 |
| HLA-B*08:01 | 71 | 79 | 9 | RAMLIAVTL | 0.237243 | 0.48 |
| HLA-A*32:01 | 211 | 219 | 9 | MSFLYAIVF | 0.155929 | 0.48 |
| HLA-B*15:01 | 106 | 114 | 9 | FLGVAGTIF | 0.364427 | 0.49 |
| HLA-B*51:01 | 75 | 83 | 9 | IAVTLASIL | 0.282724 | 0.49 |
| HLA-B*35:01 | 276 | 284 | 9 | LASLAGTAL | 0.259078 | 0.49 |
| HLA-A*01:01 | 191 | 199 | 9 | NADPVLPRL | 0.232315 | 0.49 |
| HLA-A*24:02 | 217 | 225 | 9 | IVFGGFVAF | 0.199647 | 0.49 |
| HLA-A*24:02 | 297 | 306 | 10 | VWSAATFITL | 0.19942 | 0.49 |

Rv1981c CTL epitope

| allele | start | end | length | peptide | score | percentile_rank |
| --- | --- | --- | --- | --- | --- | --- |
| HLA-A*02:03 | 4 | 12 | 9 | KLVERVHAI | 0.979822 | 0.01 |
| HLA-A*24:02 | 107 | 115 | 9 | SYSSIFSTL | 0.97312 | 0.01 |
| HLA-B*44:03 | 100 | 108 | 9 | MESVHAKSY | 0.972561 | 0.01 |
| HLA-B*44:02 | 100 | 108 | 9 | MESVHAKSY | 0.967305 | 0.01 |
| HLA-A*31:01 | 8 | 16 | 9 | RVHAINWNR | 0.965998 | 0.01 |
| HLA-A*01:01 | 236 | 244 | 9 | EIDYAHDLY | 0.95896 | 0.01 |
| HLA-A*23:01 | 107 | 115 | 9 | SYSSIFSTL | 0.942977 | 0.01 |
| HLA-B*44:02 | 216 | 224 | 9 | AERADHREY | 0.937088 | 0.01 |
| HLA-B*44:03 | 235 | 244 | 10 | NEIDYAHDLY | 0.957382 | 0.02 |
| HLA-A*02:01 | 4 | 12 | 9 | KLVERVHAI | 0.956762 | 0.02 |
| HLA-A*01:01 | 224 | 233 | 10 | YTCELLHTLY | 0.942907 | 0.02 |
| HLA-A*68:02 | 183 | 191 | 9 | NTADLIRLI | 0.907151 | 0.02 |
| HLA-A*32:01 | 61 | 69 | 9 | RVFTGLTLL | 0.785877 | 0.02 |
| HLA-B*44:03 | 216 | 224 | 9 | AERADHREY | 0.932474 | 0.03 |
| HLA-B*53:01 | 39 | 48 | 10 | IPLSNDLASW | 0.869184 | 0.03 |
| HLA-B*44:02 | 235 | 244 | 10 | NEIDYAHDLY | 0.851598 | 0.03 |
| HLA-B*08:01 | 150 | 158 | 9 | ALKRKASSV | 0.843452 | 0.03 |
| HLA-A*33:01 | 19 | 28 | 10 | DAKDLQVWER | 0.792063 | 0.03 |
| HLA-A*30:02 | 158 | 166 | 9 | VMLESFLFY | 0.791456 | 0.03 |
| HLA-A*30:02 | 196 | 204 | 9 | AVHGYYIGY | 0.772235 | 0.03 |
| HLA-A*02:06 | 4 | 12 | 9 | KLVERVHAI | 0.891611 | 0.04 |
| HLA-A*02:06 | 224 | 232 | 9 | YTCELLHTL | 0.88836 | 0.04 |
| HLA-A*02:01 | 17 | 25 | 9 | LLDAKDLQV | 0.880524 | 0.04 |
| HLA-A*68:02 | 58 | 66 | 9 | TTIRVFTGL | 0.852084 | 0.04 |
| HLA-A*26:01 | 195 | 204 | 10 | EAVHGYYIGY | 0.817275 | 0.04 |
| HLA-B*35:01 | 91 | 99 | 9 | EAVLTNMAF | 0.873062 | 0.05 |
| HLA-A*03:01 | 110 | 119 | 10 | SIFSTLCSTK | 0.85739 | 0.05 |
| HLA-B*44:02 | 54 | 63 | 10 | TEQQTTIRVF | 0.822105 | 0.05 |
| HLA-B*44:02 | 6 | 14 | 9 | VERVHAINW | 0.816866 | 0.05 |
| HLA-A*68:02 | 70 | 79 | 10 | DTAQATVGAV | 0.778033 | 0.05 |
| HLA-A*32:01 | 4 | 12 | 9 | KLVERVHAI | 0.649973 | 0.05 |
| HLA-A*02:01 | 163 | 171 | 9 | FLFYSGFYL | 0.845869 | 0.06 |
| HLA-B*44:03 | 54 | 63 | 10 | TEQQTTIRVF | 0.842382 | 0.06 |
| HLA-A*02:01 | 16 | 25 | 10 | RLLDAKDLQV | 0.84011 | 0.06 |
| HLA-A*26:01 | 196 | 204 | 9 | AVHGYYIGY | 0.71121 | 0.06 |
| HLA-B*44:03 | 6 | 14 | 9 | VERVHAINW | 0.823216 | 0.07 |
| HLA-A*02:01 | 68 | 76 | 9 | LLDTAQATV | 0.812903 | 0.07 |
| HLA-A*68:02 | 70 | 78 | 9 | DTAQATVGA | 0.743072 | 0.07 |
| HLA-A*33:01 | 200 | 208 | 9 | YYIGYKCQR | 0.698344 | 0.07 |
| HLA-A*01:01 | 235 | 244 | 10 | NEIDYAHDLY | 0.757824 | 0.08 |
| HLA-A*01:01 | 191 | 200 | 10 | IIRDEAVHGY | 0.754352 | 0.08 |
| HLA-B*08:01 | 4 | 12 | 9 | KLVERVHAI | 0.661261 | 0.08 |
| HLA-B*40:01 | 235 | 243 | 9 | NEIDYAHDL | 0.843868 | 0.09 |
| HLA-A*68:02 | 224 | 232 | 9 | YTCELLHTL | 0.683277 | 0.09 |
| HLA-B*40:01 | 89 | 97 | 9 | HEEAVLTNM | 0.815899 | 0.1 |
| HLA-A*02:06 | 61 | 69 | 9 | RVFTGLTLL | 0.718052 | 0.1 |
| HLA-A*02:03 | 150 | 158 | 9 | ALKRKASSV | 0.710694 | 0.1 |
| HLA-A*01:01 | 249 | 257 | 9 | WTDDVLPYM | 0.688609 | 0.1 |
| HLA-B*57:01 | 166 | 175 | 10 | YSGFYLPMYW | 0.903723 | 0.11 |
| HLA-B*58:01 | 167 | 175 | 9 | SGFYLPMYW | 0.821109 | 0.11 |
| HLA-B*44:03 | 235 | 243 | 9 | NEIDYAHDL | 0.74356 | 0.11 |
| HLA-A*24:02 | 106 | 115 | 10 | KSYSSIFSTL | 0.693948 | 0.11 |
| HLA-A*02:06 | 249 | 257 | 9 | WTDDVLPYM | 0.693304 | 0.11 |
| HLA-B*44:02 | 26 | 34 | 9 | WERLTGNFW | 0.687127 | 0.11 |
| HLA-A*30:02 | 137 | 145 | 9 | KAQIIVDYY | 0.596703 | 0.11 |
| HLA-A*30:01 | 98 | 106 | 9 | AFMESVHAK | 0.530634 | 0.11 |
| HLA-A*32:01 | 157 | 165 | 9 | SVMLESFLF | 0.481834 | 0.11 |
| HLA-B*58:01 | 166 | 175 | 10 | YSGFYLPMYW | 0.803393 | 0.12 |
| HLA-A*01:01 | 225 | 233 | 9 | TCELLHTLY | 0.636427 | 0.12 |
| HLA-B*08:01 | 133 | 141 | 9 | YLQRKAQII | 0.558281 | 0.12 |
| HLA-B*40:01 | 234 | 243 | 10 | ANEIDYAHDL | 0.75547 | 0.13 |
| HLA-A*33:01 | 7 | 16 | 10 | ERVHAINWNR | 0.545596 | 0.13 |
| HLA-B*57:01 | 167 | 175 | 9 | SGFYLPMYW | 0.87557 | 0.14 |
| HLA-A*02:06 | 17 | 25 | 9 | LLDAKDLQV | 0.627636 | 0.14 |
| HLA-A*23:01 | 106 | 115 | 10 | KSYSSIFSTL | 0.535252 | 0.14 |
| HLA-A*30:02 | 157 | 166 | 10 | SVMLESFLFY | 0.53255 | 0.14 |
| HLA-A*30:01 | 61 | 69 | 9 | RVFTGLTLL | 0.49815 | 0.14 |
| HLA-A*26:01 | 161 | 169 | 9 | ESFLFYSGF | 0.451659 | 0.14 |
| HLA-A*26:01 | 91 | 99 | 9 | EAVLTNMAF | 0.44171 | 0.14 |
| HLA-B*58:01 | 155 | 163 | 9 | ASSVMLESF | 0.745344 | 0.15 |
| HLA-A*03:01 | 196 | 205 | 10 | AVHGYYIGYK | 0.706738 | 0.15 |
| HLA-B*44:03 | 26 | 34 | 9 | WERLTGNFW | 0.676369 | 0.15 |
| HLA-A*02:06 | 163 | 171 | 9 | FLFYSGFYL | 0.614797 | 0.15 |
| HLA-A*02:03 | 163 | 171 | 9 | FLFYSGFYL | 0.59972 | 0.15 |
| HLA-A*33:01 | 169 | 178 | 10 | FYLPMYWSSR | 0.533841 | 0.15 |
| HLA-B*58:01 | 154 | 163 | 10 | KASSVMLESF | 0.734171 | 0.16 |
| HLA-B*15:01 | 72 | 81 | 10 | AQATVGAVAM | 0.668697 | 0.16 |
| HLA-B*15:01 | 191 | 200 | 10 | IIRDEAVHGY | 0.666952 | 0.16 |
| HLA-A*11:01 | 110 | 119 | 10 | SIFSTLCSTK | 0.664811 | 0.16 |
| HLA-A*11:01 | 196 | 205 | 10 | AVHGYYIGYK | 0.664697 | 0.16 |
| HLA-B*15:01 | 196 | 204 | 9 | AVHGYYIGY | 0.663311 | 0.16 |
| HLA-A*02:01 | 224 | 232 | 9 | YTCELLHTL | 0.649689 | 0.16 |
| HLA-A*68:02 | 277 | 285 | 9 | DTCQVNPAV | 0.547314 | 0.16 |
| HLA-A*33:01 | 277 | 286 | 10 | DTCQVNPAVR | 0.49512 | 0.16 |
| HLA-A*32:01 | 224 | 232 | 9 | YTCELLHTL | 0.394425 | 0.16 |
| HLA-B*15:01 | 55 | 63 | 9 | EQQTTIRVF | 0.662063 | 0.17 |
| HLA-B*44:03 | 90 | 99 | 10 | EEAVLTNMAF | 0.643102 | 0.17 |
| HLA-B*35:01 | 233 | 241 | 9 | YANEIDYAH | 0.600318 | 0.17 |
| HLA-B*44:02 | 90 | 99 | 10 | EEAVLTNMAF | 0.550538 | 0.17 |
| HLA-A*26:01 | 157 | 166 | 10 | SVMLESFLFY | 0.406292 | 0.17 |
| HLA-B*35:01 | 73 | 81 | 9 | QATVGAVAM | 0.580805 | 0.18 |
| HLA-A*02:06 | 16 | 25 | 10 | RLLDAKDLQV | 0.579836 | 0.18 |
| HLA-B*35:01 | 104 | 112 | 9 | HAKSYSSIF | 0.568059 | 0.18 |
| HLA-A*01:01 | 99 | 108 | 10 | FMESVHAKSY | 0.538596 | 0.18 |
| HLA-A*01:01 | 166 | 174 | 9 | YSGFYLPMY | 0.526068 | 0.18 |
| HLA-B*44:02 | 235 | 243 | 9 | NEIDYAHDL | 0.525545 | 0.18 |
| HLA-A*30:02 | 299 | 307 | 9 | FFSGSGSSY | 0.482896 | 0.18 |
| HLA-B*57:01 | 154 | 163 | 10 | KASSVMLESF | 0.823415 | 0.19 |
| HLA-B*58:01 | 119 | 127 | 9 | KQIDDAFDW | 0.692564 | 0.19 |
| HLA-B*15:01 | 158 | 166 | 9 | VMLESFLFY | 0.630647 | 0.19 |
| HLA-A*02:01 | 67 | 76 | 10 | TLLDTAQATV | 0.61551 | 0.19 |
| HLA-A*02:03 | 61 | 69 | 9 | RVFTGLTLL | 0.559715 | 0.19 |
| HLA-A*33:01 | 8 | 16 | 9 | RVHAINWNR | 0.468926 | 0.19 |
| HLA-B*53:01 | 91 | 99 | 9 | EAVLTNMAF | 0.401074 | 0.19 |
| HLA-A*32:01 | 158 | 166 | 9 | VMLESFLFY | 0.358599 | 0.19 |
| HLA-B*35:01 | 239 | 247 | 9 | YAHDLYDEL | 0.554974 | 0.2 |
| HLA-A*02:06 | 68 | 76 | 9 | LLDTAQATV | 0.549695 | 0.2 |
| HLA-A*68:02 | 223 | 232 | 10 | EYTCELLHTL | 0.491691 | 0.2 |
| HLA-A*32:01 | 119 | 127 | 9 | KQIDDAFDW | 0.349237 | 0.2 |
| HLA-A*68:01 | 7 | 16 | 10 | ERVHAINWNR | 0.802306 | 0.21 |
| HLA-A*11:01 | 8 | 16 | 9 | RVHAINWNR | 0.60371 | 0.21 |
| HLA-A*11:01 | 157 | 166 | 10 | SVMLESFLFY | 0.589844 | 0.21 |
| HLA-A*68:02 | 1 | 9 | 9 | MTGKLVERV | 0.483933 | 0.21 |
| HLA-A*68:01 | 97 | 106 | 10 | MAFMESVHAK | 0.783928 | 0.22 |
| HLA-A*30:02 | 192 | 200 | 9 | IRDEAVHGY | 0.44428 | 0.22 |
| HLA-A*24:02 | 223 | 232 | 10 | EYTCELLHTL | 0.438935 | 0.23 |
| HLA-A*32:01 | 106 | 115 | 10 | KSYSSIFSTL | 0.320919 | 0.23 |
| HLA-B*15:01 | 104 | 112 | 9 | HAKSYSSIF | 0.560907 | 0.24 |
| HLA-B*44:03 | 54 | 62 | 9 | TEQQTTIRV | 0.519265 | 0.24 |
| HLA-A*02:03 | 16 | 25 | 10 | RLLDAKDLQV | 0.505442 | 0.24 |
| HLA-A*30:02 | 136 | 144 | 9 | RKAQIIVDY | 0.426176 | 0.24 |
| HLA-A*03:01 | 158 | 166 | 9 | VMLESFLFY | 0.599224 | 0.25 |
| HLA-B*40:01 | 54 | 62 | 9 | TEQQTTIRV | 0.543671 | 0.25 |
| HLA-A*68:02 | 74 | 82 | 9 | ATVGAVAMI | 0.431146 | 0.25 |
| HLA-A*30:02 | 231 | 239 | 9 | TLYANEIDY | 0.417623 | 0.25 |
| HLA-A*01:01 | 250 | 259 | 10 | TDDVLPYMRY | 0.417395 | 0.25 |
| HLA-A*30:02 | 191 | 200 | 10 | IIRDEAVHGY | 0.416071 | 0.25 |
| HLA-B*44:02 | 25 | 34 | 10 | VWERLTGNFW | 0.415967 | 0.25 |
| HLA-B*44:02 | 54 | 62 | 9 | TEQQTTIRV | 0.412594 | 0.25 |
| HLA-A*23:01 | 157 | 165 | 9 | SVMLESFLF | 0.368244 | 0.25 |
| HLA-A*23:01 | 223 | 232 | 10 | EYTCELLHTL | 0.344613 | 0.25 |
| HLA-A*26:01 | 224 | 233 | 10 | YTCELLHTLY | 0.301057 | 0.25 |
| HLA-A*32:01 | 167 | 175 | 9 | SGFYLPMYW | 0.296063 | 0.25 |
| HLA-A*32:01 | 196 | 204 | 9 | AVHGYYIGY | 0.293113 | 0.25 |
| HLA-B*44:03 | 234 | 243 | 10 | ANEIDYAHDL | 0.471112 | 0.26 |
| HLA-A*30:02 | 162 | 170 | 9 | SFLFYSGFY | 0.404846 | 0.26 |
| HLA-A*24:02 | 25 | 33 | 9 | VWERLTGNF | 0.390578 | 0.26 |
| HLA-A*26:01 | 236 | 244 | 9 | EIDYAHDLY | 0.2915 | 0.26 |
| HLA-B*57:01 | 155 | 163 | 9 | ASSVMLESF | 0.736224 | 0.27 |
| HLA-B*51:01 | 239 | 247 | 9 | YAHDLYDEL | 0.422047 | 0.27 |
| HLA-A*68:02 | 183 | 192 | 10 | NTADLIRLII | 0.402141 | 0.27 |
| HLA-B*44:02 | 99 | 108 | 10 | FMESVHAKSY | 0.400714 | 0.27 |
| HLA-A*26:01 | 161 | 170 | 10 | ESFLFYSGFY | 0.282168 | 0.27 |
| HLA-A*68:01 | 19 | 28 | 10 | DAKDLQVWER | 0.731193 | 0.28 |
| HLA-A*68:01 | 8 | 16 | 9 | RVHAINWNR | 0.730596 | 0.28 |
| HLA-B*58:01 | 224 | 232 | 9 | YTCELLHTL | 0.57153 | 0.28 |
| HLA-A*11:01 | 196 | 204 | 9 | AVHGYYIGY | 0.519319 | 0.28 |
| HLA-A*02:06 | 85 | 93 | 9 | AVTPHEEAV | 0.476768 | 0.28 |
| HLA-A*02:01 | 61 | 69 | 9 | RVFTGLTLL | 0.472646 | 0.28 |
| HLA-A*02:03 | 17 | 25 | 9 | LLDAKDLQV | 0.455217 | 0.28 |
| HLA-A*26:01 | 58 | 66 | 9 | TTIRVFTGL | 0.264404 | 0.28 |
| HLA-B*58:01 | 137 | 145 | 9 | KAQIIVDYY | 0.537967 | 0.29 |
| HLA-B*15:01 | 231 | 239 | 9 | TLYANEIDY | 0.50561 | 0.29 |
| HLA-A*02:03 | 68 | 76 | 9 | LLDTAQATV | 0.437595 | 0.29 |
| HLA-B*35:01 | 299 | 307 | 9 | FFSGSGSSY | 0.437533 | 0.29 |
| HLA-B*44:02 | 234 | 243 | 10 | ANEIDYAHDL | 0.371478 | 0.29 |
| HLA-B*53:01 | 167 | 175 | 9 | SGFYLPMYW | 0.296815 | 0.29 |
| HLA-A*23:01 | 173 | 181 | 9 | MYWSSRGKL | 0.284479 | 0.3 |
| HLA-A*68:01 | 277 | 286 | 10 | DTCQVNPAVR | 0.703092 | 0.31 |
| HLA-B*15:01 | 117 | 125 | 9 | STKQIDDAF | 0.490813 | 0.31 |
| HLA-A*30:01 | 8 | 16 | 9 | RVHAINWNR | 0.351703 | 0.31 |
| HLA-A*24:02 | 132 | 140 | 9 | PYLQRKAQI | 0.342113 | 0.31 |
| HLA-B*57:01 | 224 | 232 | 9 | YTCELLHTL | 0.676286 | 0.32 |
| HLA-B*58:01 | 17 | 26 | 10 | LLDAKDLQVW | 0.495559 | 0.32 |
| HLA-B*44:03 | 25 | 34 | 10 | VWERLTGNFW | 0.397765 | 0.32 |
| HLA-B*44:03 | 99 | 108 | 10 | FMESVHAKSY | 0.396558 | 0.32 |
| HLA-A*30:01 | 196 | 205 | 10 | AVHGYYIGYK | 0.348877 | 0.32 |
| HLA-A*01:01 | 215 | 224 | 10 | DAERADHREY | 0.334859 | 0.32 |
| HLA-A*24:02 | 157 | 165 | 9 | SVMLESFLF | 0.322889 | 0.32 |
| HLA-A*23:01 | 25 | 33 | 9 | VWERLTGNF | 0.272544 | 0.32 |
| HLA-B*57:01 | 106 | 115 | 10 | KSYSSIFSTL | 0.668566 | 0.33 |
| HLA-A*31:01 | 138 | 146 | 9 | AQIIVDYYR | 0.524421 | 0.33 |
| HLA-A*31:01 | 200 | 208 | 9 | YYIGYKCQR | 0.52254 | 0.33 |
| HLA-B*15:01 | 54 | 63 | 10 | TEQQTTIRVF | 0.467445 | 0.33 |
| HLA-B*44:03 | 55 | 63 | 9 | EQQTTIRVF | 0.384125 | 0.33 |
| HLA-B*51:01 | 184 | 192 | 9 | TADLIRLII | 0.377166 | 0.33 |
| HLA-A*30:02 | 192 | 201 | 10 | IRDEAVHGYY | 0.359647 | 0.33 |
| HLA-A*30:01 | 106 | 114 | 9 | KSYSSIFST | 0.346195 | 0.33 |
| HLA-B*44:02 | 55 | 63 | 9 | EQQTTIRVF | 0.332579 | 0.33 |
| HLA-A*30:02 | 224 | 233 | 10 | YTCELLHTLY | 0.351814 | 0.34 |
| HLA-A*68:02 | 182 | 191 | 10 | TNTADLIRLI | 0.340698 | 0.34 |
| HLA-A*26:01 | 298 | 307 | 10 | DFFSGSGSSY | 0.216632 | 0.34 |
| HLA-A*02:03 | 224 | 232 | 9 | YTCELLHTL | 0.391426 | 0.35 |
| HLA-A*24:02 | 173 | 181 | 9 | MYWSSRGKL | 0.290781 | 0.35 |
| HLA-B*53:01 | 313 | 322 | 10 | QPTTDTDWDF | 0.250355 | 0.35 |
| HLA-A*02:03 | 67 | 76 | 10 | TLLDTAQATV | 0.385743 | 0.36 |
| HLA-B*44:03 | 89 | 97 | 9 | HEEAVLTNM | 0.355855 | 0.36 |
| HLA-A*01:01 | 137 | 145 | 9 | KAQIIVDYY | 0.310185 | 0.36 |
| HLA-B*53:01 | 239 | 247 | 9 | YAHDLYDEL | 0.242345 | 0.36 |
| HLA-B*57:01 | 119 | 127 | 9 | KQIDDAFDW | 0.629688 | 0.37 |
| HLA-A*03:01 | 8 | 16 | 9 | RVHAINWNR | 0.480009 | 0.37 |
| HLA-A*23:01 | 132 | 140 | 9 | PYLQRKAQI | 0.23274 | 0.37 |
| HLA-A*26:01 | 249 | 257 | 9 | WTDDVLPYM | 0.203128 | 0.37 |
| HLA-B*57:01 | 224 | 233 | 10 | YTCELLHTLY | 0.619204 | 0.38 |
| HLA-A*31:01 | 137 | 146 | 10 | KAQIIVDYYR | 0.489374 | 0.38 |
| HLA-A*68:02 | 239 | 247 | 9 | YAHDLYDEL | 0.324279 | 0.38 |
| HLA-A*26:01 | 117 | 125 | 9 | STKQIDDAF | 0.19858 | 0.38 |
| HLA-A*26:01 | 183 | 191 | 9 | NTADLIRLI | 0.198383 | 0.38 |
| HLA-B*58:01 | 106 | 115 | 10 | KSYSSIFSTL | 0.427597 | 0.39 |
| HLA-A*02:06 | 239 | 247 | 9 | YAHDLYDEL | 0.394195 | 0.39 |
| HLA-A*30:02 | 136 | 145 | 10 | RKAQIIVDYY | 0.319592 | 0.39 |
| HLA-A*01:01 | 158 | 166 | 9 | VMLESFLFY | 0.284186 | 0.39 |
| HLA-A*26:01 | 299 | 307 | 9 | FFSGSGSSY | 0.193268 | 0.39 |
| HLA-A*68:02 | 249 | 257 | 9 | WTDDVLPYM | 0.310728 | 0.4 |
| HLA-B*44:02 | 215 | 224 | 10 | DAERADHREY | 0.265876 | 0.4 |
| HLA-B*44:02 | 18 | 26 | 9 | LDAKDLQVW | 0.256536 | 0.4 |
| HLA-A*68:01 | 53 | 61 | 9 | STEQQTTIR | 0.635536 | 0.41 |
| HLA-B*57:01 | 5 | 14 | 10 | LVERVHAINW | 0.596136 | 0.41 |
| HLA-A*02:01 | 249 | 257 | 9 | WTDDVLPYM | 0.369067 | 0.41 |
| HLA-B*08:01 | 149 | 158 | 10 | DALKRKASSV | 0.269355 | 0.41 |
| HLA-B*57:01 | 17 | 26 | 10 | LLDAKDLQVW | 0.585687 | 0.42 |
| HLA-A*03:01 | 196 | 204 | 9 | AVHGYYIGY | 0.443033 | 0.42 |
| HLA-B*15:01 | 299 | 307 | 9 | FFSGSGSSY | 0.401336 | 0.43 |
| HLA-A*33:01 | 170 | 178 | 9 | YLPMYWSSR | 0.302261 | 0.43 |
| HLA-A*30:01 | 176 | 184 | 9 | SSRGKLTNT | 0.298895 | 0.43 |
| HLA-A*01:01 | 193 | 201 | 9 | RDEAVHGYY | 0.262329 | 0.43 |
| HLA-A*03:01 | 171 | 180 | 10 | LPMYWSSRGK | 0.425115 | 0.44 |
| HLA-B*15:01 | 99 | 108 | 10 | FMESVHAKSY | 0.386892 | 0.44 |
| HLA-A*11:01 | 158 | 166 | 9 | VMLESFLFY | 0.384934 | 0.44 |
| HLA-A*01:01 | 157 | 166 | 10 | SVMLESFLFY | 0.253592 | 0.44 |
| HLA-B*44:02 | 89 | 97 | 9 | HEEAVLTNM | 0.232068 | 0.44 |
| HLA-A*32:01 | 154 | 163 | 10 | KASSVMLESF | 0.170464 | 0.44 |
| HLA-B*40:01 | 54 | 63 | 10 | TEQQTTIRVF | 0.331037 | 0.45 |
| HLA-A*30:02 | 166 | 174 | 9 | YSGFYLPMY | 0.297708 | 0.45 |
| HLA-B*08:01 | 150 | 159 | 10 | ALKRKASSVM | 0.242772 | 0.46 |
| HLA-A*23:01 | 165 | 173 | 9 | FYSGFYLPM | 0.185049 | 0.46 |
| HLA-B*44:03 | 215 | 224 | 10 | DAERADHREY | 0.251441 | 0.47 |
| HLA-A*26:01 | 224 | 232 | 9 | YTCELLHTL | 0.156258 | 0.47 |
| HLA-B*07:02 | 280 | 289 | 10 | QVNPAVRAAL | 0.288983 | 0.48 |
| HLA-A*24:02 | 165 | 173 | 9 | FYSGFYLPM | 0.203865 | 0.48 |
| HLA-A*02:06 | 67 | 76 | 10 | TLLDTAQATV | 0.338833 | 0.49 |
| HLA-A*02:03 | 189 | 197 | 9 | RLIIRDEAV | 0.3066 | 0.49 |
| HLA-A*02:03 | 3 | 12 | 10 | GKLVERVHAI | 0.304935 | 0.49 |
| HLA-A*30:01 | 176 | 185 | 10 | SSRGKLTNTA | 0.273736 | 0.49 |
| HLA-A*26:01 | 157 | 165 | 9 | SVMLESFLF | 0.15289 | 0.49 |
| HLA-A*32:01 | 155 | 163 | 9 | ASSVMLESF | 0.152136 | 0.49 |

Rv2659c CTL epitope

| allele | start | end | length | peptide | score | percentile_rank |
| --- | --- | --- | --- | --- | --- | --- |
| HLA-B*51:01 | 257 | 265 | 9 | IPPHLIPAI | 0.952839 | 0.01 |
| HLA-A*32:01 | 33 | 41 | 9 | RVYIAPKTF | 0.91288 | 0.01 |
| HLA-A*30:01 | 229 | 237 | 9 | RVRRAVVRV | 0.86449 | 0.01 |
| HLA-A*30:02 | 343 | 351 | 9 | STAGAALRY | 0.8353 | 0.01 |
| HLA-A*01:01 | 343 | 351 | 9 | STAGAALRY | 0.949947 | 0.02 |
| HLA-A*02:03 | 332 | 340 | 9 | TLAELMQRL | 0.929207 | 0.02 |
| HLA-B*08:01 | 136 | 144 | 9 | TMRAHSYSL | 0.907439 | 0.02 |
| HLA-A*11:01 | 331 | 339 | 9 | ATLAELMQR | 0.906834 | 0.02 |
| HLA-A*68:01 | 130 | 138 | 9 | TAVGTPTMR | 0.953149 | 0.03 |
| HLA-B*07:02 | 310 | 318 | 9 | RPDLRVHDL | 0.950462 | 0.03 |
| HLA-A*68:01 | 129 | 138 | 10 | TTAVGTPTMR | 0.949039 | 0.03 |
| HLA-A*68:01 | 117 | 125 | 9 | DITPAAVRR | 0.948548 | 0.03 |
| HLA-B*35:01 | 134 | 142 | 9 | TPTMRAHSY | 0.9349 | 0.03 |
| HLA-A*02:01 | 332 | 340 | 9 | TLAELMQRL | 0.9215 | 0.03 |
| HLA-A*68:02 | 361 | 369 | 9 | EIAALLSKL | 0.884833 | 0.03 |
| HLA-B*51:01 | 192 | 200 | 9 | MPDPYQAFV | 0.84428 | 0.03 |
| HLA-A*26:01 | 343 | 351 | 9 | STAGAALRY | 0.84306 | 0.03 |
| HLA-A*33:01 | 79 | 88 | 10 | EYAEGWLKQR | 0.795937 | 0.03 |
| HLA-B*58:01 | 190 | 199 | 10 | KAMPDPYQAF | 0.946283 | 0.04 |
| HLA-A*31:01 | 14 | 22 | 9 | RIRQFNSGR | 0.861888 | 0.04 |
| HLA-A*30:01 | 93 | 101 | 9 | RTRAHYRKL | 0.715487 | 0.04 |
| HLA-B*35:01 | 202 | 210 | 9 | MAAWLAMRY | 0.884976 | 0.05 |
| HLA-A*02:06 | 35 | 43 | 9 | YIAPKTFNA | 0.867835 | 0.05 |
| HLA-A*31:01 | 138 | 146 | 9 | RAHSYSLLR | 0.847552 | 0.05 |
| HLA-B*15:01 | 191 | 199 | 9 | AMPDPYQAF | 0.840513 | 0.05 |
| HLA-A*31:01 | 331 | 339 | 9 | ATLAELMQR | 0.83659 | 0.05 |
| HLA-A*02:03 | 35 | 43 | 9 | YIAPKTFNA | 0.827312 | 0.05 |
| HLA-A*24:02 | 191 | 199 | 9 | AMPDPYQAF | 0.812506 | 0.05 |
| HLA-B*51:01 | 194 | 202 | 9 | DPYQAFVLM | 0.766175 | 0.05 |
| HLA-B*08:01 | 215 | 223 | 9 | ELRRKDIDL | 0.755698 | 0.05 |
| HLA-B*53:01 | 75 | 84 | 10 | APFGEYAEGW | 0.713243 | 0.05 |
| HLA-B*57:01 | 190 | 199 | 10 | KAMPDPYQAF | 0.953385 | 0.06 |
| HLA-B*57:01 | 118 | 126 | 9 | ITPAAVRRW | 0.952847 | 0.06 |
| HLA-A*02:06 | 180 | 188 | 9 | ATLDELETI | 0.858125 | 0.06 |
| HLA-A*02:06 | 332 | 340 | 9 | TLAELMQRL | 0.843143 | 0.06 |
| HLA-A*01:01 | 27 | 35 | 9 | YTGPDGRVY | 0.822267 | 0.06 |
| HLA-A*33:01 | 117 | 125 | 9 | DITPAAVRR | 0.733551 | 0.06 |
| HLA-B*08:01 | 310 | 318 | 9 | RPDLRVHDL | 0.720495 | 0.06 |
| HLA-B*53:01 | 42 | 50 | 9 | NAKIDAEAW | 0.702531 | 0.06 |
| HLA-B*53:01 | 274 | 282 | 9 | NPGRESLLF | 0.697421 | 0.06 |
| HLA-A*30:01 | 168 | 176 | 9 | ASTARRVHK | 0.631662 | 0.06 |
| HLA-B*58:01 | 118 | 126 | 9 | ITPAAVRRW | 0.860941 | 0.08 |
| HLA-A*11:01 | 295 | 303 | 9 | SALYRMFYK | 0.789586 | 0.08 |
| HLA-B*15:01 | 33 | 41 | 9 | RVYIAPKTF | 0.78438 | 0.08 |
| HLA-A*31:01 | 291 | 299 | 9 | HLAPSALYR | 0.767019 | 0.08 |
| HLA-A*68:02 | 226 | 234 | 9 | EVARVRRAV | 0.718241 | 0.08 |
| HLA-A*23:01 | 191 | 199 | 9 | AMPDPYQAF | 0.716537 | 0.08 |
| HLA-A*26:01 | 342 | 351 | 10 | HSTAGAALRY | 0.6039 | 0.08 |
| HLA-B*53:01 | 192 | 201 | 10 | MPDPYQAFVL | 0.599604 | 0.08 |
| HLA-A*26:01 | 361 | 369 | 9 | EIAALLSKL | 0.595778 | 0.08 |
| HLA-A*02:01 | 35 | 43 | 9 | YIAPKTFNA | 0.790634 | 0.09 |
| HLA-A*03:01 | 290 | 299 | 10 | RHLAPSALYR | 0.789219 | 0.09 |
| HLA-A*03:01 | 291 | 299 | 9 | HLAPSALYR | 0.788456 | 0.09 |
| HLA-B*07:02 | 272 | 280 | 9 | HVNPGRESL | 0.772576 | 0.09 |
| HLA-A*01:01 | 342 | 351 | 10 | HSTAGAALRY | 0.732871 | 0.09 |
| HLA-B*40:01 | 360 | 369 | 10 | REIAALLSKL | 0.80786 | 0.1 |
| HLA-A*02:01 | 100 | 108 | 9 | KLLDNHILA | 0.752772 | 0.1 |
| HLA-B*35:01 | 194 | 202 | 9 | DPYQAFVLM | 0.739567 | 0.1 |
| HLA-B*53:01 | 192 | 200 | 9 | MPDPYQAFV | 0.565183 | 0.1 |
| HLA-B*07:02 | 37 | 45 | 9 | APKTFNAKI | 0.740907 | 0.11 |
| HLA-B*44:02 | 69 | 77 | 9 | QEDRPGAPF | 0.689284 | 0.11 |
| HLA-A*02:03 | 207 | 216 | 10 | AMRYGELTEL | 0.659182 | 0.11 |
| HLA-B*51:01 | 249 | 257 | 9 | DAGVRDISI | 0.6296 | 0.11 |
| HLA-A*30:02 | 290 | 298 | 9 | RHLAPSALY | 0.602441 | 0.11 |
| HLA-A*30:02 | 27 | 35 | 9 | YTGPDGRVY | 0.59756 | 0.11 |
| HLA-A*33:01 | 156 | 164 | 9 | DLIDSNPCR | 0.588465 | 0.11 |
| HLA-B*08:01 | 174 | 182 | 9 | VHKIRPATL | 0.566935 | 0.11 |
| HLA-A*26:01 | 202 | 210 | 9 | MAAWLAMRY | 0.505674 | 0.11 |
| HLA-B*44:03 | 69 | 77 | 9 | QEDRPGAPF | 0.730503 | 0.12 |
| HLA-A*02:06 | 256 | 264 | 9 | SIPPHLIPA | 0.678486 | 0.12 |
| HLA-A*68:02 | 254 | 262 | 9 | DISIPPHLI | 0.60739 | 0.12 |
| HLA-A*30:01 | 138 | 146 | 9 | RAHSYSLLR | 0.518854 | 0.12 |
| HLA-B*53:01 | 134 | 142 | 9 | TPTMRAHSY | 0.512881 | 0.12 |
| HLA-A*32:01 | 190 | 199 | 10 | KAMPDPYQAF | 0.474222 | 0.12 |
| HLA-A*31:01 | 1 | 9 | 9 | VTQTGKRQR | 0.706228 | 0.13 |
| HLA-B*07:02 | 293 | 301 | 9 | APSALYRMF | 0.68316 | 0.13 |
| HLA-A*33:01 | 291 | 299 | 9 | HLAPSALYR | 0.559532 | 0.13 |
| HLA-B*53:01 | 197 | 205 | 9 | QAFVLMAAW | 0.497861 | 0.13 |
| HLA-A*68:01 | 342 | 350 | 9 | HSTAGAALR | 0.85794 | 0.14 |
| HLA-B*40:01 | 277 | 285 | 9 | RESLLFPSV | 0.749324 | 0.14 |
| HLA-A*02:06 | 100 | 108 | 9 | KLLDNHILA | 0.638218 | 0.14 |
| HLA-A*30:01 | 173 | 181 | 9 | RVHKIRPAT | 0.491197 | 0.14 |
| HLA-B*40:01 | 69 | 77 | 9 | QEDRPGAPF | 0.712975 | 0.15 |
| HLA-A*11:01 | 343 | 351 | 9 | STAGAALRY | 0.686716 | 0.15 |
| HLA-B*35:01 | 274 | 282 | 9 | NPGRESLLF | 0.619647 | 0.15 |
| HLA-A*24:02 | 190 | 199 | 10 | KAMPDPYQAF | 0.592281 | 0.15 |
| HLA-A*68:02 | 35 | 43 | 9 | YIAPKTFNA | 0.565275 | 0.15 |
| HLA-A*30:02 | 342 | 351 | 10 | HSTAGAALRY | 0.527021 | 0.15 |
| HLA-A*30:02 | 202 | 210 | 9 | MAAWLAMRY | 0.526462 | 0.15 |
| HLA-A*68:01 | 291 | 299 | 9 | HLAPSALYR | 0.842765 | 0.16 |
| HLA-A*68:01 | 108 | 116 | 9 | ATFADTDLR | 0.838026 | 0.16 |
| HLA-B*08:01 | 205 | 213 | 9 | WLAMRYGEL | 0.483651 | 0.16 |
| HLA-B*53:01 | 293 | 301 | 9 | APSALYRMF | 0.456146 | 0.16 |
| HLA-B*58:01 | 197 | 205 | 9 | QAFVLMAAW | 0.721472 | 0.17 |
| HLA-A*03:01 | 348 | 356 | 9 | ALRYQHAAK | 0.694784 | 0.17 |
| HLA-B*35:01 | 72 | 80 | 9 | RPGAPFGEY | 0.597675 | 0.17 |
| HLA-B*35:01 | 119 | 127 | 9 | TPAAVRRWY | 0.592285 | 0.17 |
| HLA-A*02:06 | 190 | 198 | 9 | KAMPDPYQA | 0.587033 | 0.17 |
| HLA-B*57:01 | 33 | 41 | 9 | RVYIAPKTF | 0.833465 | 0.18 |
| HLA-A*31:01 | 209 | 217 | 9 | RYGELTELR | 0.647907 | 0.18 |
| HLA-A*68:02 | 272 | 280 | 9 | HVNPGRESL | 0.516533 | 0.18 |
| HLA-A*30:01 | 348 | 356 | 9 | ALRYQHAAK | 0.45457 | 0.18 |
| HLA-A*30:01 | 176 | 185 | 10 | KIRPATLDEL | 0.450989 | 0.18 |
| HLA-B*57:01 | 197 | 205 | 9 | QAFVLMAAW | 0.823244 | 0.19 |
| HLA-A*68:02 | 284 | 292 | 9 | SVNDPNRHL | 0.504114 | 0.19 |
| HLA-A*23:01 | 190 | 199 | 10 | KAMPDPYQAF | 0.470149 | 0.19 |
| HLA-B*53:01 | 194 | 202 | 9 | DPYQAFVLM | 0.402094 | 0.19 |
| HLA-B*58:01 | 33 | 41 | 9 | RVYIAPKTF | 0.688594 | 0.2 |
| HLA-A*31:01 | 290 | 299 | 10 | RHLAPSALYR | 0.628902 | 0.2 |
| HLA-B*15:01 | 190 | 199 | 10 | KAMPDPYQAF | 0.622469 | 0.2 |
| HLA-B*35:01 | 192 | 201 | 10 | MPDPYQAFVL | 0.551668 | 0.2 |
| HLA-A*68:02 | 129 | 137 | 9 | TTAVGTPTM | 0.489669 | 0.2 |
| HLA-A*30:01 | 14 | 22 | 9 | RIRQFNSGR | 0.437923 | 0.2 |
| HLA-A*30:01 | 234 | 242 | 9 | VVRVGEGFK | 0.433161 | 0.2 |
| HLA-A*26:01 | 129 | 137 | 9 | TTAVGTPTM | 0.361971 | 0.2 |
| HLA-A*11:01 | 25 | 33 | 9 | ASYTGPDGR | 0.602538 | 0.21 |
| HLA-A*11:01 | 108 | 116 | 9 | ATFADTDLR | 0.598667 | 0.21 |
| HLA-A*02:01 | 207 | 216 | 10 | AMRYGELTEL | 0.586234 | 0.21 |
| HLA-A*02:03 | 256 | 264 | 9 | SIPPHLIPA | 0.539632 | 0.21 |
| HLA-A*01:01 | 19 | 27 | 9 | NSGRWQASY | 0.474241 | 0.21 |
| HLA-A*31:01 | 164 | 172 | 9 | RISGASTAR | 0.616333 | 0.22 |
| HLA-B*15:01 | 27 | 35 | 9 | YTGPDGRVY | 0.582581 | 0.22 |
| HLA-B*35:01 | 27 | 35 | 9 | YTGPDGRVY | 0.53438 | 0.22 |
| HLA-A*30:02 | 26 | 35 | 10 | SYTGPDGRVY | 0.444203 | 0.22 |
| HLA-A*68:01 | 156 | 164 | 9 | DLIDSNPCR | 0.773077 | 0.23 |
| HLA-A*11:01 | 291 | 299 | 9 | HLAPSALYR | 0.565165 | 0.23 |
| HLA-A*02:03 | 331 | 340 | 10 | ATLAELMQRL | 0.514003 | 0.23 |
| HLA-A*33:01 | 48 | 56 | 9 | EAWLTDRRR | 0.429621 | 0.23 |
| HLA-A*30:01 | 295 | 303 | 9 | SALYRMFYK | 0.418959 | 0.23 |
| HLA-B*53:01 | 202 | 210 | 9 | MAAWLAMRY | 0.356638 | 0.23 |
| HLA-A*26:01 | 27 | 35 | 9 | YTGPDGRVY | 0.316732 | 0.23 |
| HLA-A*32:01 | 191 | 199 | 9 | AMPDPYQAF | 0.316041 | 0.23 |
| HLA-B*15:01 | 89 | 98 | 10 | GIKDRTRAHY | 0.56173 | 0.24 |
| HLA-A*02:03 | 221 | 230 | 10 | IDLHGEVARV | 0.501885 | 0.24 |
| HLA-B*35:01 | 192 | 200 | 9 | MPDPYQAFV | 0.471044 | 0.24 |
| HLA-A*68:02 | 169 | 177 | 9 | STARRVHKI | 0.442418 | 0.24 |
| HLA-A*30:02 | 188 | 196 | 9 | ITKAMPDPY | 0.431782 | 0.24 |
| HLA-A*68:02 | 332 | 340 | 9 | TLAELMQRL | 0.431505 | 0.24 |
| HLA-A*31:01 | 350 | 358 | 9 | RYQHAAKGR | 0.583232 | 0.25 |
| HLA-B*40:01 | 253 | 261 | 9 | RDISIPPHL | 0.531373 | 0.25 |
| HLA-B*07:02 | 192 | 201 | 10 | MPDPYQAFVL | 0.480309 | 0.25 |
| HLA-A*23:01 | 33 | 41 | 9 | RVYIAPKTF | 0.341008 | 0.25 |
| HLA-A*30:01 | 33 | 41 | 9 | RVYIAPKTF | 0.396862 | 0.26 |
| HLA-A*68:01 | 239 | 247 | 9 | EGFKVTTPK | 0.736205 | 0.27 |
| HLA-A*03:01 | 14 | 22 | 9 | RIRQFNSGR | 0.566063 | 0.27 |
| HLA-A*03:01 | 35 | 44 | 10 | YIAPKTFNAK | 0.563008 | 0.27 |
| HLA-A*02:03 | 144 | 152 | 9 | LLRAIMQTA | 0.471446 | 0.27 |
| HLA-B*08:01 | 227 | 235 | 9 | VARVRRAVV | 0.368193 | 0.27 |
| HLA-B*53:01 | 257 | 265 | 9 | IPPHLIPAI | 0.317061 | 0.27 |
| HLA-A*32:01 | 14 | 23 | 10 | RIRQFNSGRW | 0.273873 | 0.27 |
| HLA-A*26:01 | 191 | 199 | 9 | AMPDPYQAF | 0.271783 | 0.27 |
| HLA-A*02:03 | 191 | 200 | 10 | AMPDPYQAFV | 0.458994 | 0.28 |
| HLA-A*02:03 | 100 | 108 | 9 | KLLDNHILA | 0.451039 | 0.28 |
| HLA-B*08:01 | 272 | 280 | 9 | HVNPGRESL | 0.364942 | 0.28 |
| HLA-B*15:01 | 343 | 351 | 9 | STAGAALRY | 0.504813 | 0.29 |
| HLA-A*02:01 | 180 | 188 | 9 | ATLDELETI | 0.46663 | 0.29 |
| HLA-A*02:06 | 196 | 204 | 9 | YQAFVLMAA | 0.465927 | 0.29 |
| HLA-B*07:02 | 72 | 80 | 9 | RPGAPFGEY | 0.449032 | 0.29 |
| HLA-A*01:01 | 202 | 210 | 9 | MAAWLAMRY | 0.377037 | 0.29 |
| HLA-B*08:01 | 317 | 325 | 9 | DLRHSGAVL | 0.35626 | 0.29 |
| HLA-A*32:01 | 140 | 148 | 9 | HSYSLLRAI | 0.26141 | 0.29 |
| HLA-A*11:01 | 138 | 146 | 9 | RAHSYSLLR | 0.499319 | 0.3 |
| HLA-B*53:01 | 119 | 127 | 9 | TPAAVRRWY | 0.286 | 0.3 |
| HLA-A*32:01 | 118 | 126 | 9 | ITPAAVRRW | 0.251679 | 0.3 |
| HLA-B*58:01 | 42 | 50 | 9 | NAKIDAEAW | 0.521903 | 0.31 |
| HLA-B*07:02 | 136 | 144 | 9 | TMRAHSYSL | 0.433998 | 0.31 |
| HLA-A*68:01 | 331 | 339 | 9 | ATLAELMQR | 0.696931 | 0.32 |
| HLA-A*03:01 | 331 | 339 | 9 | ATLAELMQR | 0.514321 | 0.32 |
| HLA-A*33:01 | 130 | 138 | 9 | TAVGTPTMR | 0.369319 | 0.32 |
| HLA-A*33:01 | 297 | 305 | 9 | LYRMFYKAR | 0.367125 | 0.32 |
| HLA-A*03:01 | 295 | 303 | 9 | SALYRMFYK | 0.503348 | 0.33 |
| HLA-B*58:01 | 27 | 35 | 9 | YTGPDGRVY | 0.484285 | 0.33 |
| HLA-A*11:01 | 35 | 44 | 10 | YIAPKTFNAK | 0.469865 | 0.33 |
| HLA-A*11:01 | 168 | 176 | 9 | ASTARRVHK | 0.469116 | 0.33 |
| HLA-A*30:02 | 33 | 41 | 9 | RVYIAPKTF | 0.358304 | 0.33 |
| HLA-A*32:01 | 169 | 177 | 9 | STARRVHKI | 0.233109 | 0.33 |
| HLA-A*02:01 | 260 | 269 | 10 | HLIPAIEDHL | 0.419674 | 0.34 |
| HLA-B*51:01 | 254 | 262 | 9 | DISIPPHLI | 0.371505 | 0.34 |
| HLA-A*30:02 | 19 | 27 | 9 | NSGRWQASY | 0.351857 | 0.34 |
| HLA-A*33:01 | 201 | 209 | 9 | LMAAWLAMR | 0.349453 | 0.34 |
| HLA-A*32:01 | 173 | 182 | 10 | RVHKIRPATL | 0.223389 | 0.34 |
| HLA-A*02:01 | 256 | 264 | 9 | SIPPHLIPA | 0.416199 | 0.35 |
| HLA-A*02:01 | 191 | 200 | 10 | AMPDPYQAFV | 0.407718 | 0.35 |
| HLA-A*02:03 | 136 | 144 | 9 | TMRAHSYSL | 0.387991 | 0.35 |
| HLA-B*35:01 | 293 | 301 | 9 | APSALYRMF | 0.366912 | 0.35 |
| HLA-A*01:01 | 26 | 35 | 10 | SYTGPDGRVY | 0.312701 | 0.35 |
| HLA-A*01:01 | 294 | 302 | 9 | PSALYRMFY | 0.312642 | 0.35 |
| HLA-A*02:01 | 331 | 340 | 10 | ATLAELMQRL | 0.403873 | 0.36 |
| HLA-B*44:02 | 265 | 273 | 9 | IEDHLHKHV | 0.294271 | 0.36 |
| HLA-B*08:01 | 50 | 58 | 9 | WLTDRRREI | 0.28582 | 0.36 |
| HLA-B*53:01 | 41 | 50 | 10 | FNAKIDAEAW | 0.240674 | 0.36 |
| HLA-A*32:01 | 284 | 292 | 9 | SVNDPNRHL | 0.210017 | 0.36 |
| HLA-B*51:01 | 140 | 148 | 9 | HSYSLLRAI | 0.361097 | 0.37 |
| HLA-A*30:01 | 299 | 307 | 9 | RMFYKARKA | 0.323643 | 0.37 |
| HLA-A*68:01 | 48 | 56 | 9 | EAWLTDRRR | 0.648771 | 0.38 |
| HLA-A*31:01 | 25 | 33 | 9 | ASYTGPDGR | 0.49137 | 0.38 |
| HLA-A*31:01 | 306 | 314 | 9 | KAAGRPDLR | 0.489267 | 0.38 |
| HLA-B*58:01 | 41 | 50 | 10 | FNAKIDAEAW | 0.44041 | 0.38 |
| HLA-A*11:01 | 233 | 242 | 10 | AVVRVGEGFK | 0.432409 | 0.38 |
| HLA-B*35:01 | 129 | 137 | 9 | TTAVGTPTM | 0.337349 | 0.38 |
| HLA-B*35:01 | 262 | 270 | 9 | IPAIEDHLH | 0.337182 | 0.38 |
| HLA-A*30:01 | 314 | 322 | 9 | RVHDLRHSG | 0.321606 | 0.38 |
| HLA-A*31:01 | 116 | 125 | 10 | RDITPAAVRR | 0.480929 | 0.39 |
| HLA-B*15:01 | 188 | 196 | 9 | ITKAMPDPY | 0.439461 | 0.39 |
| HLA-A*24:02 | 33 | 41 | 9 | RVYIAPKTF | 0.260556 | 0.39 |
| HLA-A*03:01 | 164 | 172 | 9 | RISGASTAR | 0.461259 | 0.4 |
| HLA-B*40:01 | 265 | 273 | 9 | IEDHLHKHV | 0.381578 | 0.4 |
| HLA-A*02:03 | 296 | 304 | 9 | ALYRMFYKA | 0.3596 | 0.4 |
| HLA-B*07:02 | 176 | 185 | 10 | KIRPATLDEL | 0.353386 | 0.4 |
| HLA-B*51:01 | 192 | 201 | 10 | MPDPYQAFVL | 0.347669 | 0.4 |
| HLA-A*30:01 | 39 | 47 | 9 | KTFNAKIDA | 0.310983 | 0.4 |
| HLA-B*58:01 | 202 | 210 | 9 | MAAWLAMRY | 0.405143 | 0.41 |
| HLA-B*58:01 | 343 | 351 | 9 | STAGAALRY | 0.397576 | 0.41 |
| HLA-A*02:01 | 100 | 109 | 10 | KLLDNHILAT | 0.372074 | 0.41 |
| HLA-B*51:01 | 37 | 45 | 9 | APKTFNAKI | 0.341859 | 0.41 |
| HLA-A*03:01 | 138 | 146 | 9 | RAHSYSLLR | 0.44192 | 0.42 |
| HLA-B*15:01 | 233 | 241 | 9 | AVVRVGEGF | 0.407074 | 0.42 |
| HLA-A*33:01 | 222 | 231 | 10 | DLHGEVARVR | 0.304975 | 0.42 |
| HLA-B*35:01 | 343 | 351 | 9 | STAGAALRY | 0.297483 | 0.42 |
| HLA-B*44:03 | 183 | 192 | 10 | DELETITKAM | 0.289014 | 0.42 |
| HLA-A*32:01 | 272 | 280 | 9 | HVNPGRESL | 0.18382 | 0.42 |
| HLA-A*32:01 | 233 | 241 | 9 | AVVRVGEGF | 0.183812 | 0.42 |
| HLA-A*32:01 | 136 | 144 | 9 | TMRAHSYSL | 0.180468 | 0.42 |
| HLA-B*57:01 | 42 | 50 | 9 | NAKIDAEAW | 0.578423 | 0.43 |
| HLA-B*07:02 | 257 | 265 | 9 | IPPHLIPAI | 0.336985 | 0.43 |
| HLA-A*30:02 | 89 | 98 | 10 | GIKDRTRAHY | 0.303419 | 0.43 |
| HLA-B*35:01 | 325 | 333 | 9 | LAASTGATL | 0.293924 | 0.43 |
| HLA-A*01:01 | 188 | 196 | 9 | ITKAMPDPY | 0.265198 | 0.43 |
| HLA-B*57:01 | 14 | 23 | 10 | RIRQFNSGRW | 0.573623 | 0.44 |
| HLA-A*02:03 | 260 | 269 | 10 | HLIPAIEDHL | 0.335509 | 0.44 |
| HLA-A*33:01 | 301 | 310 | 10 | FYKARKAAGR | 0.295785 | 0.44 |
| HLA-A*32:01 | 180 | 188 | 9 | ATLDELETI | 0.174406 | 0.44 |
| HLA-A*32:01 | 229 | 237 | 9 | RVRRAVVRV | 0.170196 | 0.44 |
| HLA-A*26:01 | 284 | 292 | 9 | SVNDPNRHL | 0.167925 | 0.44 |
| HLA-A*68:01 | 35 | 44 | 10 | YIAPKTFNAK | 0.607046 | 0.45 |
| HLA-B*57:01 | 41 | 50 | 10 | FNAKIDAEAW | 0.561354 | 0.45 |
| HLA-A*31:01 | 209 | 218 | 10 | RYGELTELRR | 0.439637 | 0.45 |
| HLA-A*02:01 | 101 | 109 | 9 | LLDNHILAT | 0.351351 | 0.45 |
| HLA-A*02:01 | 296 | 304 | 9 | ALYRMFYKA | 0.351346 | 0.45 |
| HLA-B*40:01 | 68 | 77 | 10 | GQEDRPGAPF | 0.325408 | 0.45 |
| HLA-A*68:02 | 256 | 264 | 9 | SIPPHLIPA | 0.287313 | 0.45 |
| HLA-A*24:02 | 141 | 149 | 9 | SYSLLRAIM | 0.224139 | 0.45 |
| HLA-B*53:01 | 190 | 199 | 10 | KAMPDPYQAF | 0.189165 | 0.45 |
| HLA-A*32:01 | 343 | 351 | 9 | STAGAALRY | 0.167251 | 0.45 |
| HLA-A*68:02 | 244 | 252 | 9 | TTPKSDAGV | 0.284725 | 0.46 |
| HLA-A*32:01 | 93 | 101 | 9 | RTRAHYRKL | 0.162505 | 0.46 |
| HLA-A*31:01 | 201 | 209 | 9 | LMAAWLAMR | 0.429731 | 0.47 |
| HLA-A*11:01 | 290 | 299 | 10 | RHLAPSALYR | 0.369081 | 0.47 |
| HLA-A*02:03 | 284 | 292 | 9 | SVNDPNRHL | 0.316498 | 0.47 |
| HLA-A*30:01 | 314 | 323 | 10 | RVHDLRHSGA | 0.283421 | 0.47 |
| HLA-A*30:01 | 173 | 182 | 10 | RVHKIRPATL | 0.282038 | 0.47 |
| HLA-B*44:02 | 277 | 285 | 9 | RESLLFPSV | 0.20855 | 0.47 |
| HLA-A*26:01 | 233 | 241 | 9 | AVVRVGEGF | 0.157835 | 0.47 |
| HLA-A*02:06 | 207 | 216 | 10 | AMRYGELTEL | 0.345252 | 0.48 |
| HLA-A*02:01 | 143 | 151 | 9 | SLLRAIMQT | 0.333921 | 0.48 |
| HLA-A*30:01 | 93 | 102 | 10 | RTRAHYRKLL | 0.277789 | 0.48 |
| HLA-B*08:01 | 134 | 142 | 9 | TPTMRAHSY | 0.235958 | 0.48 |
| HLA-A*01:01 | 89 | 98 | 10 | GIKDRTRAHY | 0.235739 | 0.48 |
| HLA-A*02:06 | 284 | 292 | 9 | SVNDPNRHL | 0.336518 | 0.49 |
| HLA-B*44:03 | 265 | 273 | 9 | IEDHLHKHV | 0.238707 | 0.49 |
| HLA-B*44:03 | 277 | 285 | 9 | RESLLFPSV | 0.237504 | 0.49 |
| HLA-A*01:01 | 133 | 142 | 10 | GTPTMRAHSY | 0.230645 | 0.49 |

Rv2660

| allele | start | end | length | peptide | score | rank |
| --- | --- | --- | --- | --- | --- | --- |
| HLA-A*32:01 | 41 | 49 | 9 | VVAPSQFTF | 0.90762 | 0.01 |
| HLA-B*58:01 | 41 | 49 | 9 | VVAPSQFTF | 0.952816 | 0.03 |
| HLA-A*24:02 | 41 | 49 | 9 | VVAPSQFTF | 0.821483 | 0.05 |
| HLA-A*23:01 | 41 | 49 | 9 | VVAPSQFTF | 0.803451 | 0.05 |
| HLA-B*15:01 | 41 | 49 | 9 | VVAPSQFTF | 0.809266 | 0.06 |
| HLA-B*53:01 | 56 | 65 | 10 | FVDETAGQSW | 0.6602 | 0.07 |
| HLA-B*57:01 | 41 | 49 | 9 | VVAPSQFTF | 0.925143 | 0.09 |
| HLA-B*53:01 | 41 | 49 | 9 | VVAPSQFTF | 0.559762 | 0.1 |
| HLA-A*32:01 | 40 | 49 | 10 | SVVAPSQFTF | 0.488822 | 0.11 |
| HLA-B*58:01 | 39 | 47 | 9 | LSVVAPSQF | 0.791753 | 0.12 |
| HLA-B*35:01 | 41 | 49 | 9 | VVAPSQFTF | 0.712026 | 0.12 |
| HLA-A*68:02 | 59 | 68 | 10 | ETAGQSWCAI | 0.555502 | 0.15 |
| HLA-A*68:02 | 59 | 67 | 9 | ETAGQSWCA | 0.551131 | 0.15 |
| HLA-B*58:01 | 56 | 65 | 10 | FVDETAGQSW | 0.693881 | 0.19 |
| HLA-A*26:01 | 41 | 49 | 9 | VVAPSQFTF | 0.358403 | 0.2 |
| HLA-B*15:01 | 40 | 49 | 10 | SVVAPSQFTF | 0.595732 | 0.21 |
| HLA-B*57:01 | 39 | 47 | 9 | LSVVAPSQF | 0.785713 | 0.22 |
| HLA-B*44:02 | 57 | 65 | 9 | VDETAGQSW | 0.431898 | 0.24 |
| HLA-A*26:01 | 40 | 49 | 10 | SVVAPSQFTF | 0.301533 | 0.25 |
| HLA-A*24:02 | 48 | 56 | 9 | TFSSRSPDF | 0.349917 | 0.3 |
| HLA-B*58:01 | 40 | 49 | 10 | SVVAPSQFTF | 0.517263 | 0.31 |
| HLA-A*30:02 | 41 | 49 | 9 | VVAPSQFTF | 0.369945 | 0.31 |
| HLA-B*44:03 | 57 | 65 | 9 | VDETAGQSW | 0.362921 | 0.35 |
| HLA-A*23:01 | 48 | 56 | 9 | TFSSRSPDF | 0.235105 | 0.37 |
| HLA-B*57:01 | 56 | 65 | 10 | FVDETAGQSW | 0.62058 | 0.38 |
| HLA-B*57:01 | 40 | 49 | 10 | SVVAPSQFTF | 0.586662 | 0.42 |
| HLA-B*40:01 | 34 | 42 | 9 | TEQRNLSVV | 0.351512 | 0.43 |
| HLA-A*68:02 | 22 | 30 | 9 | ASGGVTVGV | 0.279336 | 0.47 |
| HLA-A*68:01 | 10 | 18 | 9 | AATGQASQR | 0.593476 | 0.49 |
| HLA-A*31:01 | 43 | 52 | 10 | APSQFTFSSR | 0.418592 | 0.49 |

Rv3879c CTL epitope

| allele | start | end | length | peptide | score | percentile_rank |
| --- | --- | --- | --- | --- | --- | --- |
| HLA-B*07:02 | 325 | 333 | 9 | APHVKPAAL | 0.996311 | 0.01 |
| HLA-B*53:01 | 562 | 570 | 9 | YPVLAVQAW | 0.982768 | 0.01 |
| HLA-A*68:01 | 696 | 705 | 10 | HTATDAAVQR | 0.978639 | 0.01 |
| HLA-B*44:03 | 157 | 166 | 10 | AERVLESKNW | 0.973225 | 0.01 |
| HLA-B*44:02 | 157 | 166 | 10 | AERVLESKNW | 0.971546 | 0.01 |
| HLA-A*11:01 | 589 | 597 | 9 | ASSDPGVAK | 0.962478 | 0.01 |
| HLA-B*08:01 | 325 | 333 | 9 | APHVKPAAL | 0.960859 | 0.01 |
| HLA-A*02:01 | 83 | 91 | 9 | TLQDYLATV | 0.973291 | 0.02 |
| HLA-A*68:01 | 673 | 681 | 9 | EAAHLRAFR | 0.966798 | 0.02 |
| HLA-A*68:01 | 155 | 164 | 10 | ETAERVLESK | 0.963776 | 0.02 |
| HLA-A*68:01 | 697 | 705 | 9 | TATDAAVQR | 0.960506 | 0.02 |
| HLA-A*02:03 | 83 | 91 | 9 | TLQDYLATV | 0.946474 | 0.02 |
| HLA-A*26:01 | 26 | 35 | 10 | DTFYDRAQEY | 0.924946 | 0.02 |
| HLA-B*44:02 | 672 | 680 | 9 | REAAHLRAF | 0.912342 | 0.02 |
| HLA-A*33:01 | 673 | 681 | 9 | EAAHLRAFR | 0.849197 | 0.02 |
| HLA-B*58:01 | 706 | 714 | 9 | VAVADWLYW | 0.963877 | 0.03 |
| HLA-B*40:01 | 536 | 544 | 9 | MELPNKVYL | 0.954549 | 0.03 |
| HLA-A*68:01 | 646 | 654 | 9 | DVNPPGDER | 0.952433 | 0.03 |
| HLA-A*02:06 | 83 | 91 | 9 | TLQDYLATV | 0.926133 | 0.03 |
| HLA-B*44:03 | 672 | 680 | 9 | REAAHLRAF | 0.922083 | 0.03 |
| HLA-B*35:01 | 523 | 531 | 9 | VANSYGLAY | 0.919238 | 0.03 |
| HLA-A*23:01 | 86 | 94 | 9 | DYLATVITW | 0.875582 | 0.03 |
| HLA-B*51:01 | 485 | 493 | 9 | DALRLARRI | 0.850655 | 0.03 |
| HLA-B*51:01 | 534 | 542 | 9 | DGMELPNKV | 0.840317 | 0.03 |
| HLA-B*07:02 | 296 | 304 | 9 | APSPGPQPV | 0.937057 | 0.04 |
| HLA-B*07:02 | 203 | 211 | 9 | TPITPGTPI | 0.926891 | 0.04 |
| HLA-B*07:02 | 209 | 217 | 9 | TPITPGTPI | 0.926891 | 0.04 |
| HLA-B*07:02 | 284 | 292 | 9 | APAPHPQPA | 0.899656 | 0.04 |
| HLA-A*01:01 | 499 | 507 | 9 | ASDNNAGDY | 0.886387 | 0.04 |
| HLA-A*24:02 | 86 | 94 | 9 | DYLATVITW | 0.879832 | 0.04 |
| HLA-A*68:02 | 618 | 626 | 9 | EVVDPSAAA | 0.85899 | 0.04 |
| HLA-A*31:01 | 458 | 466 | 9 | SMIPVSAAR | 0.851783 | 0.04 |
| HLA-A*24:02 | 530 | 538 | 9 | AYIPDGMEL | 0.841756 | 0.04 |
| HLA-A*30:02 | 27 | 35 | 9 | TFYDRAQEY | 0.757854 | 0.04 |
| HLA-B*57:01 | 706 | 714 | 9 | VAVADWLYW | 0.959367 | 0.05 |
| HLA-B*35:01 | 562 | 570 | 9 | YPVLAVQAW | 0.889506 | 0.05 |
| HLA-B*07:02 | 243 | 251 | 9 | KPVTPVTPV | 0.886962 | 0.05 |
| HLA-A*23:01 | 530 | 538 | 9 | AYIPDGMEL | 0.773808 | 0.05 |
| HLA-A*30:01 | 159 | 167 | 9 | RVLESKNWK | 0.683981 | 0.05 |
| HLA-B*58:01 | 705 | 714 | 10 | RVAVADWLYW | 0.899827 | 0.06 |
| HLA-B*07:02 | 273 | 281 | 9 | TPATPATPV | 0.856955 | 0.06 |
| HLA-A*02:01 | 635 | 643 | 9 | RLLDLLPPA | 0.852738 | 0.06 |
| HLA-B*07:02 | 181 | 190 | 10 | SPPPPDVPTL | 0.85226 | 0.06 |
| HLA-A*03:01 | 159 | 167 | 9 | RVLESKNWK | 0.834078 | 0.06 |
| HLA-A*01:01 | 708 | 716 | 9 | VADWLYWQY | 0.828662 | 0.06 |
| HLA-B*51:01 | 130 | 138 | 9 | DADERHTAI | 0.739063 | 0.06 |
| HLA-B*51:01 | 700 | 708 | 9 | DAAVQRVAV | 0.722589 | 0.06 |
| HLA-A*30:02 | 523 | 531 | 9 | VANSYGLAY | 0.705251 | 0.06 |
| HLA-A*30:02 | 2 | 10 | 9 | SITRPTGSY | 0.698509 | 0.06 |
| HLA-B*53:01 | 561 | 570 | 10 | TYPVLAVQAW | 0.691909 | 0.06 |
| HLA-A*30:01 | 589 | 597 | 9 | ASSDPGVAK | 0.631064 | 0.06 |
| HLA-A*30:01 | 3 | 11 | 9 | ITRPTGSYA | 0.624954 | 0.06 |
| HLA-A*68:01 | 549 | 558 | 10 | HAIPVDEIAR | 0.904462 | 0.07 |
| HLA-B*07:02 | 286 | 294 | 9 | APHPQPAPA | 0.847566 | 0.07 |
| HLA-A*02:06 | 635 | 643 | 9 | RLLDLLPPA | 0.830667 | 0.07 |
| HLA-B*07:02 | 197 | 205 | 9 | TPGTPGTPI | 0.825961 | 0.07 |
| HLA-B*07:02 | 237 | 245 | 9 | TPVTPGKPV | 0.823304 | 0.07 |
| HLA-A*68:02 | 449 | 457 | 9 | ESADDGTPV | 0.749738 | 0.07 |
| HLA-B*53:01 | 503 | 511 | 9 | NAGDYGFFW | 0.662198 | 0.07 |
| HLA-B*57:01 | 705 | 714 | 10 | RVAVADWLYW | 0.928475 | 0.08 |
| HLA-A*03:01 | 589 | 597 | 9 | ASSDPGVAK | 0.810236 | 0.08 |
| HLA-B*07:02 | 240 | 248 | 9 | TPGKPVTPV | 0.801666 | 0.08 |
| HLA-A*33:01 | 458 | 466 | 9 | SMIPVSAAR | 0.67197 | 0.08 |
| HLA-B*53:01 | 706 | 714 | 9 | VAVADWLYW | 0.603479 | 0.08 |
| HLA-A*26:01 | 2 | 10 | 9 | SITRPTGSY | 0.589459 | 0.08 |
| HLA-A*02:01 | 543 | 551 | 9 | YLASADHAI | 0.788323 | 0.09 |
| HLA-B*07:02 | 270 | 278 | 9 | APATPATPA | 0.783443 | 0.09 |
| HLA-A*11:01 | 159 | 167 | 9 | RVLESKNWK | 0.776764 | 0.09 |
| HLA-B*07:02 | 5 | 14 | 10 | RPTGSYARQM | 0.769851 | 0.09 |
| HLA-A*01:01 | 20 | 29 | 10 | WVEADEDTFY | 0.727985 | 0.09 |
| HLA-B*51:01 | 203 | 211 | 9 | TPITPGTPI | 0.665884 | 0.09 |
| HLA-B*51:01 | 209 | 217 | 9 | TPITPGTPI | 0.665884 | 0.09 |
| HLA-A*33:01 | 646 | 654 | 9 | DVNPPGDER | 0.646287 | 0.09 |
| HLA-A*30:02 | 705 | 713 | 9 | RVAVADWLY | 0.620329 | 0.09 |
| HLA-A*31:01 | 3 | 12 | 10 | ITRPTGSYAR | 0.739622 | 0.1 |
| HLA-A*24:02 | 561 | 570 | 10 | TYPVLAVQAW | 0.698726 | 0.1 |
| HLA-A*02:03 | 543 | 551 | 9 | YLASADHAI | 0.684906 | 0.1 |
| HLA-A*68:02 | 152 | 160 | 9 | LVAETAERV | 0.659965 | 0.1 |
| HLA-A*01:01 | 523 | 531 | 9 | VANSYGLAY | 0.659034 | 0.1 |
| HLA-A*23:01 | 85 | 94 | 10 | QDYLATVITW | 0.651633 | 0.1 |
| HLA-B*51:01 | 220 | 228 | 9 | IPGAPVTPI | 0.647369 | 0.1 |
| HLA-A*30:02 | 519 | 527 | 9 | GSIVVANSY | 0.615303 | 0.1 |
| HLA-A*02:01 | 639 | 647 | 9 | LLPPAPVDV | 0.736757 | 0.11 |
| HLA-B*07:02 | 288 | 296 | 9 | HPQPAPAPA | 0.718973 | 0.11 |
| HLA-A*02:03 | 488 | 496 | 9 | RLARRIAAA | 0.67176 | 0.11 |
| HLA-A*23:01 | 561 | 570 | 10 | TYPVLAVQAW | 0.603661 | 0.11 |
| HLA-A*30:02 | 707 | 716 | 10 | AVADWLYWQY | 0.596358 | 0.11 |
| HLA-A*30:02 | 522 | 531 | 10 | VVANSYGLAY | 0.570521 | 0.11 |
| HLA-B*08:01 | 130 | 138 | 9 | DADERHTAI | 0.568049 | 0.11 |
| HLA-A*68:01 | 473 | 481 | 9 | TAAASARQR | 0.866279 | 0.12 |
| HLA-B*40:01 | 535 | 544 | 10 | GMELPNKVYL | 0.778098 | 0.12 |
| HLA-A*03:01 | 243 | 252 | 10 | KPVTPVTPVK | 0.766111 | 0.12 |
| HLA-B*07:02 | 420 | 428 | 9 | APSTRAASA | 0.699493 | 0.12 |
| HLA-A*02:03 | 639 | 647 | 9 | LLPPAPVDV | 0.645869 | 0.12 |
| HLA-A*33:01 | 88 | 96 | 9 | LATVITWHR | 0.57214 | 0.12 |
| HLA-A*30:01 | 243 | 252 | 10 | KPVTPVTPVK | 0.513143 | 0.12 |
| HLA-A*26:01 | 707 | 716 | 10 | AVADWLYWQY | 0.497877 | 0.12 |
| HLA-B*15:01 | 2 | 10 | 9 | SITRPTGSY | 0.697358 | 0.13 |
| HLA-B*07:02 | 212 | 220 | 9 | TPGTPITPI | 0.681228 | 0.13 |
| HLA-A*02:06 | 639 | 647 | 9 | LLPPAPVDV | 0.659989 | 0.13 |
| HLA-A*24:02 | 682 | 690 | 9 | AYAAHSQEI | 0.646528 | 0.13 |
| HLA-A*24:02 | 85 | 94 | 10 | QDYLATVITW | 0.636905 | 0.13 |
| HLA-B*51:01 | 181 | 190 | 10 | SPPPPDVPTL | 0.596168 | 0.13 |
| HLA-A*30:02 | 1 | 10 | 10 | MSITRPTGSY | 0.554979 | 0.13 |
| HLA-B*07:02 | 220 | 228 | 9 | IPGAPVTPI | 0.672651 | 0.14 |
| HLA-B*07:02 | 276 | 284 | 9 | TPATPVTPA | 0.670495 | 0.14 |
| HLA-B*58:01 | 503 | 511 | 9 | NAGDYGFFW | 0.739628 | 0.15 |
| HLA-B*40:01 | 672 | 680 | 9 | REAAHLRAF | 0.723532 | 0.15 |
| HLA-B*44:03 | 554 | 562 | 9 | DEIARCATY | 0.677374 | 0.15 |
| HLA-B*15:01 | 523 | 531 | 9 | VANSYGLAY | 0.675562 | 0.15 |
| HLA-B*07:02 | 231 | 239 | 9 | TPGTPVTPV | 0.653743 | 0.15 |
| HLA-A*02:06 | 543 | 551 | 9 | YLASADHAI | 0.616544 | 0.15 |
| HLA-A*02:03 | 73 | 81 | 9 | ALGANINQL | 0.605835 | 0.15 |
| HLA-B*44:02 | 671 | 680 | 10 | GREAAHLRAF | 0.56814 | 0.15 |
| HLA-A*68:01 | 571 | 579 | 9 | AAFHDMTLR | 0.842733 | 0.16 |
| HLA-A*02:03 | 661 | 669 | 9 | LMKPMTSTA | 0.589183 | 0.16 |
| HLA-A*26:01 | 618 | 626 | 9 | EVVDPSAAA | 0.419921 | 0.16 |
| HLA-B*44:03 | 536 | 544 | 9 | MELPNKVYL | 0.625272 | 0.17 |
| HLA-A*02:06 | 580 | 588 | 9 | AVIGTAEQL | 0.591358 | 0.17 |
| HLA-A*02:03 | 635 | 643 | 9 | RLLDLLPPA | 0.58319 | 0.17 |
| HLA-B*44:02 | 554 | 562 | 9 | DEIARCATY | 0.54285 | 0.17 |
| HLA-A*68:02 | 97 | 105 | 9 | HIAGLIEQA | 0.532267 | 0.17 |
| HLA-A*68:02 | 357 | 365 | 9 | ESAASVTPA | 0.528436 | 0.17 |
| HLA-A*30:01 | 427 | 435 | 9 | SARTAPPAR | 0.459345 | 0.17 |
| HLA-B*44:03 | 21 | 29 | 9 | VEADEDTFY | 0.624629 | 0.18 |
| HLA-A*02:06 | 152 | 160 | 9 | LVAETAERV | 0.582349 | 0.18 |
| HLA-B*35:01 | 565 | 573 | 9 | LAVQAWAAF | 0.579954 | 0.18 |
| HLA-A*02:06 | 698 | 706 | 9 | ATDAAVQRV | 0.576833 | 0.18 |
| HLA-A*02:03 | 719 | 727 | 9 | GLLDRALAA | 0.572225 | 0.18 |
| HLA-A*01:01 | 498 | 507 | 10 | NASDNNAGDY | 0.544196 | 0.18 |
| HLA-A*26:01 | 27 | 35 | 9 | TFYDRAQEY | 0.392091 | 0.18 |
| HLA-B*15:01 | 519 | 527 | 9 | GSIVVANSY | 0.634034 | 0.19 |
| HLA-B*15:01 | 522 | 531 | 10 | VVANSYGLAY | 0.623136 | 0.19 |
| HLA-A*02:01 | 73 | 81 | 9 | ALGANINQL | 0.617123 | 0.19 |
| HLA-A*01:01 | 26 | 35 | 10 | DTFYDRAQEY | 0.510734 | 0.19 |
| HLA-B*44:02 | 536 | 544 | 9 | MELPNKVYL | 0.504463 | 0.19 |
| HLA-B*08:01 | 165 | 173 | 9 | NWKPPKNAL | 0.450548 | 0.19 |
| HLA-A*11:01 | 243 | 252 | 10 | KPVTPVTPVK | 0.605985 | 0.2 |
| HLA-A*31:01 | 427 | 435 | 9 | SARTAPPAR | 0.62798 | 0.21 |
| HLA-B*15:01 | 535 | 543 | 9 | GMELPNKVY | 0.612954 | 0.21 |
| HLA-B*07:02 | 489 | 497 | 9 | LARRIAAAL | 0.543096 | 0.21 |
| HLA-A*68:02 | 142 | 150 | 9 | VTATHGANV | 0.474524 | 0.21 |
| HLA-A*24:02 | 529 | 538 | 10 | LAYIPDGMEL | 0.465177 | 0.21 |
| HLA-A*24:02 | 34 | 42 | 9 | EYSQVLQRV | 0.463532 | 0.21 |
| HLA-A*26:01 | 1 | 10 | 10 | MSITRPTGSY | 0.3454 | 0.21 |
| HLA-A*31:01 | 571 | 579 | 9 | AAFHDMTLR | 0.609884 | 0.22 |
| HLA-A*31:01 | 669 | 678 | 10 | ATGREAAHLR | 0.607324 | 0.22 |
| HLA-B*44:03 | 671 | 680 | 10 | GREAAHLRAF | 0.537865 | 0.22 |
| HLA-B*07:02 | 272 | 281 | 10 | ATPATPATPV | 0.523821 | 0.22 |
| HLA-B*51:01 | 273 | 281 | 9 | TPATPATPV | 0.467868 | 0.22 |
| HLA-A*32:01 | 705 | 714 | 10 | RVAVADWLYW | 0.325646 | 0.22 |
| HLA-B*15:01 | 479 | 487 | 9 | RQRGRGDAL | 0.5732 | 0.23 |
| HLA-A*02:01 | 719 | 727 | 9 | GLLDRALAA | 0.559531 | 0.23 |
| HLA-B*07:02 | 591 | 600 | 10 | SDPGVAKIVL | 0.511868 | 0.23 |
| HLA-B*35:01 | 27 | 35 | 9 | TFYDRAQEY | 0.492164 | 0.23 |
| HLA-B*44:02 | 21 | 29 | 9 | VEADEDTFY | 0.445382 | 0.23 |
| HLA-A*23:01 | 682 | 690 | 9 | AYAAHSQEI | 0.383255 | 0.23 |
| HLA-B*53:01 | 562 | 571 | 10 | YPVLAVQAWA | 0.353127 | 0.23 |
| HLA-B*51:01 | 562 | 570 | 9 | YPVLAVQAW | 0.445604 | 0.24 |
| HLA-A*01:01 | 707 | 716 | 10 | AVADWLYWQY | 0.442046 | 0.24 |
| HLA-A*30:01 | 2 | 11 | 10 | SITRPTGSYA | 0.40846 | 0.24 |
| HLA-A*23:01 | 529 | 538 | 10 | LAYIPDGMEL | 0.376044 | 0.24 |
| HLA-A*68:01 | 458 | 466 | 9 | SMIPVSAAR | 0.758748 | 0.25 |
| HLA-A*68:01 | 234 | 243 | 10 | TPVTPVTPGK | 0.751785 | 0.25 |
| HLA-A*31:01 | 41 | 50 | 10 | RVTDVLDTCR | 0.588906 | 0.25 |
| HLA-A*11:01 | 234 | 243 | 10 | TPVTPVTPGK | 0.554339 | 0.25 |
| HLA-B*07:02 | 325 | 334 | 10 | APHVKPAALA | 0.489315 | 0.25 |
| HLA-B*35:01 | 218 | 226 | 9 | TPIPGAPVT | 0.467314 | 0.25 |
| HLA-B*51:01 | 212 | 220 | 9 | TPGTPITPI | 0.444553 | 0.25 |
| HLA-A*68:02 | 358 | 366 | 9 | SAASVTPAA | 0.427958 | 0.25 |
| HLA-A*68:02 | 545 | 553 | 9 | ASADHAIPV | 0.422116 | 0.25 |
| HLA-A*23:01 | 34 | 42 | 9 | EYSQVLQRV | 0.357862 | 0.25 |
| HLA-A*23:01 | 712 | 720 | 9 | LYWQYVTGL | 0.356281 | 0.25 |
| HLA-A*32:01 | 144 | 152 | 9 | ATHGANVSL | 0.304159 | 0.25 |
| HLA-A*32:01 | 705 | 713 | 9 | RVAVADWLY | 0.303603 | 0.25 |
| HLA-A*11:01 | 571 | 579 | 9 | AAFHDMTLR | 0.541027 | 0.26 |
| HLA-A*02:01 | 587 | 595 | 9 | QLASSDPGV | 0.504221 | 0.26 |
| HLA-B*44:03 | 688 | 696 | 9 | QEIALHQAH | 0.475372 | 0.26 |
| HLA-B*51:01 | 296 | 304 | 9 | APSPGPQPV | 0.432853 | 0.26 |
| HLA-B*51:01 | 237 | 245 | 9 | TPVTPGKPV | 0.427878 | 0.26 |
| HLA-A*33:01 | 697 | 705 | 9 | TATDAAVQR | 0.414518 | 0.26 |
| HLA-A*30:01 | 429 | 437 | 9 | RTAPPARPP | 0.393618 | 0.26 |
| HLA-A*26:01 | 518 | 527 | 10 | DGSIVVANSY | 0.294828 | 0.26 |
| HLA-A*31:01 | 672 | 681 | 10 | REAAHLRAFR | 0.572048 | 0.27 |
| HLA-A*31:01 | 457 | 466 | 10 | VSMIPVSAAR | 0.5716 | 0.27 |
| HLA-A*11:01 | 588 | 597 | 10 | LASSDPGVAK | 0.525006 | 0.27 |
| HLA-B*35:01 | 708 | 716 | 9 | VADWLYWQY | 0.442964 | 0.28 |
| HLA-B*53:01 | 86 | 94 | 9 | DYLATVITW | 0.309625 | 0.28 |
| HLA-B*53:01 | 649 | 658 | 10 | PPGDERHMLW | 0.303841 | 0.28 |
| HLA-A*03:01 | 655 | 663 | 9 | HMLWFELMK | 0.553012 | 0.29 |
| HLA-A*11:01 | 172 | 180 | 9 | ALEDLLQQK | 0.508793 | 0.29 |
| HLA-A*02:01 | 80 | 88 | 9 | QLMTLQDYL | 0.463024 | 0.29 |
| HLA-A*02:03 | 151 | 160 | 10 | SLVAETAERV | 0.449357 | 0.29 |
| HLA-B*51:01 | 240 | 248 | 9 | TPGKPVTPV | 0.411559 | 0.29 |
| HLA-A*01:01 | 1 | 10 | 10 | MSITRPTGSY | 0.38098 | 0.29 |
| HLA-A*32:01 | 12 | 20 | 9 | RQMLDPGGW | 0.268685 | 0.29 |
| HLA-A*68:01 | 457 | 466 | 10 | VSMIPVSAAR | 0.714207 | 0.3 |
| HLA-A*68:01 | 88 | 96 | 9 | LATVITWHR | 0.712573 | 0.3 |
| HLA-A*68:01 | 472 | 481 | 10 | ATAAASARQR | 0.710765 | 0.3 |
| HLA-A*03:01 | 172 | 180 | 9 | ALEDLLQQK | 0.531466 | 0.3 |
| HLA-B*07:02 | 324 | 333 | 10 | PAPHVKPAAL | 0.437727 | 0.3 |
| HLA-A*01:01 | 698 | 706 | 9 | ATDAAVQRV | 0.362653 | 0.3 |
| HLA-A*01:01 | 705 | 713 | 9 | RVAVADWLY | 0.358544 | 0.3 |
| HLA-B*08:01 | 489 | 497 | 9 | LARRIAAAL | 0.34756 | 0.3 |
| HLA-B*40:01 | 257 | 265 | 9 | GEPTPITPV | 0.456537 | 0.31 |
| HLA-A*02:06 | 545 | 553 | 9 | ASADHAIPV | 0.455898 | 0.31 |
| HLA-A*02:03 | 719 | 728 | 10 | GLLDRALAAA | 0.421514 | 0.31 |
| HLA-A*68:02 | 135 | 144 | 10 | HTAINSLVTA | 0.367155 | 0.31 |
| HLA-A*68:01 | 44 | 53 | 10 | DVLDTCRQQK | 0.70163 | 0.32 |
| HLA-A*68:01 | 366 | 374 | 9 | AASGVPGAR | 0.698954 | 0.32 |
| HLA-A*68:01 | 470 | 479 | 10 | DAATAAASAR | 0.694978 | 0.32 |
| HLA-B*58:01 | 705 | 713 | 9 | RVAVADWLY | 0.496119 | 0.32 |
| HLA-B*07:02 | 300 | 308 | 9 | GPQPVTPAT | 0.41859 | 0.32 |
| HLA-B*07:02 | 167 | 176 | 10 | KPPKNALEDL | 0.415066 | 0.32 |
| HLA-A*02:03 | 82 | 91 | 10 | MTLQDYLATV | 0.414669 | 0.32 |
| HLA-A*02:03 | 587 | 595 | 9 | QLASSDPGV | 0.414534 | 0.32 |
| HLA-B*07:02 | 223 | 231 | 9 | APVTPITPT | 0.413009 | 0.32 |
| HLA-B*35:01 | 288 | 296 | 9 | HPQPAPAPA | 0.39796 | 0.32 |
| HLA-B*51:01 | 243 | 251 | 9 | KPVTPVTPV | 0.3794 | 0.32 |
| HLA-A*68:02 | 700 | 708 | 9 | DAAVQRVAV | 0.366108 | 0.32 |
| HLA-A*68:02 | 136 | 144 | 9 | TAINSLVTA | 0.362356 | 0.32 |
| HLA-A*31:01 | 32 | 41 | 10 | AQEYSQVLQR | 0.524855 | 0.33 |
| HLA-B*15:01 | 1 | 10 | 10 | MSITRPTGSY | 0.468526 | 0.33 |
| HLA-A*33:01 | 3 | 12 | 10 | ITRPTGSYAR | 0.358402 | 0.33 |
| HLA-A*68:02 | 144 | 152 | 9 | ATHGANVSL | 0.353799 | 0.33 |
| HLA-A*68:02 | 357 | 366 | 10 | ESAASVTPAA | 0.353534 | 0.33 |
| HLA-A*30:02 | 708 | 716 | 9 | VADWLYWQY | 0.35299 | 0.33 |
| HLA-A*30:01 | 144 | 152 | 9 | ATHGANVSL | 0.342962 | 0.33 |
| HLA-A*68:01 | 627 | 635 | 9 | QLADTTDQR | 0.677729 | 0.34 |
| HLA-B*57:01 | 503 | 511 | 9 | NAGDYGFFW | 0.659436 | 0.34 |
| HLA-A*02:01 | 82 | 91 | 10 | MTLQDYLATV | 0.422942 | 0.34 |
| HLA-B*07:02 | 202 | 211 | 10 | GTPITPGTPI | 0.401426 | 0.34 |
| HLA-B*07:02 | 208 | 217 | 10 | GTPITPGTPI | 0.401426 | 0.34 |
| HLA-A*02:03 | 140 | 148 | 9 | SLVTATHGA | 0.400543 | 0.34 |
| HLA-B*51:01 | 354 | 362 | 9 | HADESAASV | 0.372304 | 0.34 |
| HLA-A*24:02 | 712 | 720 | 9 | LYWQYVTGL | 0.30302 | 0.34 |
| HLA-B*08:01 | 612 | 620 | 9 | TGRSRLEVV | 0.302701 | 0.34 |
| HLA-A*24:02 | 542 | 551 | 10 | VYLASADHAI | 0.300771 | 0.34 |
| HLA-B*07:02 | 370 | 378 | 9 | VPGARAAAA | 0.39452 | 0.35 |
| HLA-B*35:01 | 203 | 211 | 9 | TPITPGTPI | 0.366569 | 0.35 |
| HLA-B*35:01 | 209 | 217 | 9 | TPITPGTPI | 0.366569 | 0.35 |
| HLA-A*33:01 | 549 | 558 | 10 | HAIPVDEIAR | 0.343819 | 0.35 |
| HLA-A*33:01 | 87 | 96 | 10 | YLATVITWHR | 0.34281 | 0.35 |
| HLA-B*44:02 | 688 | 696 | 9 | QEIALHQAH | 0.310129 | 0.35 |
| HLA-B*08:01 | 700 | 708 | 9 | DAAVQRVAV | 0.290529 | 0.35 |
| HLA-A*02:06 | 560 | 569 | 10 | ATYPVLAVQA | 0.41441 | 0.36 |
| HLA-A*02:06 | 331 | 339 | 9 | AALAEQPGV | 0.413556 | 0.36 |
| HLA-A*02:06 | 97 | 105 | 9 | HIAGLIEQA | 0.413272 | 0.36 |
| HLA-B*07:02 | 298 | 307 | 10 | SPGPQPVTPA | 0.381152 | 0.36 |
| HLA-A*33:01 | 670 | 678 | 9 | TGREAAHLR | 0.335439 | 0.36 |
| HLA-A*68:02 | 155 | 163 | 9 | ETAERVLES | 0.333387 | 0.36 |
| HLA-A*68:02 | 361 | 370 | 10 | SVTPAAASGV | 0.332007 | 0.36 |
| HLA-A*68:01 | 97 | 106 | 10 | HIAGLIEQAK | 0.656284 | 0.37 |
| HLA-B*35:01 | 325 | 333 | 9 | APHVKPAAL | 0.349084 | 0.37 |
| HLA-A*30:02 | 535 | 543 | 9 | GMELPNKVY | 0.329739 | 0.37 |
| HLA-A*33:01 | 427 | 435 | 9 | SARTAPPAR | 0.323251 | 0.37 |
| HLA-B*44:02 | 85 | 94 | 10 | QDYLATVITW | 0.285659 | 0.37 |
| HLA-B*53:01 | 181 | 190 | 10 | SPPPPDVPTL | 0.23796 | 0.37 |
| HLA-A*26:01 | 522 | 531 | 10 | VVANSYGLAY | 0.20336 | 0.37 |
| HLA-A*02:03 | 152 | 160 | 9 | LVAETAERV | 0.373025 | 0.38 |
| HLA-B*51:01 | 183 | 191 | 9 | PPPDVPTLV | 0.355806 | 0.38 |
| HLA-B*51:01 | 231 | 239 | 9 | TPGTPVTPV | 0.354321 | 0.38 |
| HLA-A*68:02 | 82 | 91 | 10 | MTLQDYLATV | 0.326061 | 0.38 |
| HLA-A*68:02 | 560 | 569 | 10 | ATYPVLAVQA | 0.323292 | 0.38 |
| HLA-A*68:02 | 151 | 160 | 10 | SLVAETAERV | 0.32087 | 0.38 |
| HLA-A*01:01 | 522 | 531 | 10 | VVANSYGLAY | 0.297854 | 0.38 |
| HLA-B*53:01 | 203 | 211 | 9 | TPITPGTPI | 0.23309 | 0.38 |
| HLA-B*53:01 | 209 | 217 | 9 | TPITPGTPI | 0.23309 | 0.38 |
| HLA-B*53:01 | 523 | 531 | 9 | VANSYGLAY | 0.230955 | 0.38 |
| HLA-A*32:01 | 707 | 716 | 10 | AVADWLYWQY | 0.198458 | 0.38 |
| HLA-A*32:01 | 702 | 711 | 10 | AVQRVAVADW | 0.195716 | 0.38 |
| HLA-A*03:01 | 45 | 53 | 9 | VLDTCRQQK | 0.468602 | 0.39 |
| HLA-A*03:01 | 32 | 41 | 10 | AQEYSQVLQR | 0.46827 | 0.39 |
| HLA-A*02:03 | 97 | 105 | 9 | HIAGLIEQA | 0.36495 | 0.39 |
| HLA-B*35:01 | 282 | 290 | 9 | TPAPAPHPQ | 0.326564 | 0.39 |
| HLA-B*44:03 | 33 | 42 | 10 | QEYSQVLQRV | 0.319175 | 0.39 |
| HLA-A*68:02 | 515 | 523 | 9 | VTTDGSIVV | 0.315547 | 0.39 |
| HLA-A*01:01 | 590 | 598 | 9 | SSDPGVAKI | 0.288186 | 0.39 |
| HLA-B*15:01 | 27 | 35 | 9 | TFYDRAQEY | 0.437763 | 0.4 |
| HLA-A*68:02 | 404 | 412 | 9 | HAATGRAPV | 0.309346 | 0.4 |
| HLA-A*68:02 | 354 | 362 | 9 | HADESAASV | 0.306938 | 0.4 |
| HLA-A*31:01 | 426 | 435 | 10 | ASARTAPPAR | 0.470752 | 0.41 |
| HLA-B*51:01 | 260 | 269 | 10 | TPITPVTPPV | 0.341439 | 0.41 |
| HLA-A*33:01 | 696 | 705 | 10 | HTATDAAVQR | 0.309995 | 0.41 |
| HLA-B*35:01 | 229 | 237 | 9 | TPTPGTPVT | 0.302628 | 0.41 |
| HLA-A*68:02 | 263 | 272 | 10 | TPVTPPVAPA | 0.30126 | 0.41 |
| HLA-A*26:01 | 20 | 29 | 10 | WVEADEDTFY | 0.181532 | 0.41 |
| HLA-A*68:01 | 589 | 597 | 9 | ASSDPGVAK | 0.628044 | 0.42 |
| HLA-A*01:01 | 631 | 639 | 9 | TTDQRLLDL | 0.269229 | 0.42 |
| HLA-A*68:01 | 365 | 374 | 10 | AAASGVPGAR | 0.622283 | 0.43 |
| HLA-B*15:01 | 79 | 87 | 9 | NQLMTLQDY | 0.397585 | 0.43 |
| HLA-A*11:01 | 472 | 481 | 10 | ATAAASARQR | 0.396055 | 0.43 |
| HLA-B*58:01 | 519 | 527 | 9 | GSIVVANSY | 0.371998 | 0.43 |
| HLA-A*02:06 | 82 | 91 | 10 | MTLQDYLATV | 0.371604 | 0.43 |
| HLA-A*02:03 | 577 | 585 | 9 | TLRAVIGTA | 0.341625 | 0.43 |
| HLA-B*07:02 | 308 | 316 | 9 | TPGPSGPAT | 0.332213 | 0.43 |
| HLA-B*07:02 | 460 | 468 | 9 | IPVSAARAA | 0.33191 | 0.43 |
| HLA-B*07:02 | 270 | 279 | 10 | APATPATPAT | 0.331285 | 0.43 |
| HLA-B*07:02 | 292 | 300 | 9 | APAPAPSPG | 0.330794 | 0.43 |
| HLA-B*51:01 | 182 | 190 | 9 | PPPPDVPTL | 0.327732 | 0.43 |
| HLA-A*68:02 | 90 | 98 | 9 | TVITWHRHI | 0.292037 | 0.43 |
| HLA-A*68:02 | 143 | 152 | 10 | TATHGANVSL | 0.290783 | 0.43 |
| HLA-A*01:01 | 2 | 10 | 9 | SITRPTGSY | 0.26217 | 0.43 |
| HLA-B*08:01 | 479 | 487 | 9 | RQRGRGDAL | 0.25902 | 0.43 |
| HLA-B*53:01 | 708 | 716 | 9 | VADWLYWQY | 0.197757 | 0.43 |
| HLA-B*57:01 | 702 | 711 | 10 | AVQRVAVADW | 0.573803 | 0.44 |
| HLA-A*02:06 | 590 | 598 | 9 | SSDPGVAKI | 0.3667 | 0.44 |
| HLA-B*35:01 | 450 | 459 | 10 | SADDGTPVSM | 0.285318 | 0.44 |
| HLA-B*44:02 | 33 | 42 | 10 | QEYSQVLQRV | 0.234621 | 0.44 |
| HLA-A*23:01 | 542 | 551 | 10 | VYLASADHAI | 0.194102 | 0.44 |
| HLA-A*02:03 | 627 | 636 | 10 | QLADTTDQRL | 0.328143 | 0.45 |
| HLA-A*30:01 | 422 | 431 | 10 | STRAASARTA | 0.290895 | 0.45 |
| HLA-B*35:01 | 296 | 304 | 9 | APSPGPQPV | 0.277692 | 0.45 |
| HLA-B*44:03 | 85 | 94 | 10 | QDYLATVITW | 0.265224 | 0.45 |
| HLA-A*03:01 | 234 | 243 | 10 | TPVTPVTPGK | 0.412804 | 0.46 |
| HLA-A*11:01 | 32 | 41 | 10 | AQEYSQVLQR | 0.377289 | 0.46 |
| HLA-B*07:02 | 275 | 284 | 10 | ATPATPVTPA | 0.312786 | 0.46 |
| HLA-B*07:02 | 377 | 385 | 9 | AAAPSGTAV | 0.305919 | 0.46 |
| HLA-A*68:02 | 580 | 588 | 9 | AVIGTAEQL | 0.281093 | 0.46 |
| HLA-A*02:06 | 377 | 385 | 9 | AAAPSGTAV | 0.351688 | 0.47 |
| HLA-A*02:06 | 354 | 362 | 9 | HADESAASV | 0.348508 | 0.47 |
| HLA-B*07:02 | 431 | 439 | 9 | APPARPPST | 0.301279 | 0.47 |
| HLA-B*07:02 | 31 | 39 | 9 | RAQEYSQVL | 0.299787 | 0.47 |
| HLA-A*68:02 | 537 | 545 | 9 | ELPNKVYLA | 0.27958 | 0.47 |
| HLA-A*68:01 | 645 | 654 | 10 | VDVNPPGDER | 0.597785 | 0.48 |
| HLA-A*03:01 | 458 | 466 | 9 | SMIPVSAAR | 0.403481 | 0.48 |
| HLA-A*02:06 | 587 | 595 | 9 | QLASSDPGV | 0.344983 | 0.48 |
| HLA-A*02:06 | 73 | 81 | 9 | ALGANINQL | 0.340957 | 0.48 |
| HLA-A*02:01 | 719 | 728 | 10 | GLLDRALAAA | 0.332641 | 0.48 |
| HLA-A*30:01 | 429 | 438 | 10 | RTAPPARPPS | 0.280421 | 0.48 |
| HLA-A*31:01 | 159 | 167 | 9 | RVLESKNWK | 0.420136 | 0.49 |
| HLA-B*07:02 | 260 | 269 | 10 | TPITPVTPPV | 0.285577 | 0.49 |
| HLA-A*68:02 | 149 | 157 | 9 | NVSLVAETA | 0.269478 | 0.49 |
| HLA-B*44:03 | 334 | 343 | 10 | AEQPGVPGQH | 0.238781 | 0.49 |
| HLA-A*01:01 | 535 | 543 | 9 | GMELPNKVY | 0.230665 | 0.49 |
