# Supplementary material for: In silico Analysis of Peptide-Based Biomarkers for the Diagnosis and Prevention of Latent Tuberculosis Infection
Source: Front Microbiol. 2022 Jun 28;13:947852. doi: 10.3389/fmicb.2022.947852 (PMC9273951; doi:10.3389/fmicb.2022.947852)
Supplement: Supplementary Figure 1 — The polypeptide molecular sequence was electronically cloned into the pET30a expression vector by SnapGene software. The diagnostic antigen is the gene coding for the insertion of antigen molecules, and the rest is the expression vector. [file Data_Sheet_1.zip › Supplementary Files/Table S3.docx]

Rv1737c CTL epitope

| epitope | length | Immunogenicity | antigenicity |
| --- | --- | --- | --- |
| VVNFWAWNL | 9 | 0.55139 | 2.3392 |
| ISVVNFWAW | 9 | 0.36864 | 1.4305 |
| LPVTWEMSF | 9 | 0.11755 | 1.4137 |
| MSFLYAIVF | 9 | 0.19651 | 1.3973 |
| RLPVTWEMSF | 10 | 0.14083 | 1.1827 |
| GSYALLVFF | 9 | 0.12017 | 1.1592 |
| AAAGGLGGY | 9 | 0.11776 | 1.146 |
| GARTAGFAL | 9 | 0.24752 | 1.1123 |
| LLVATPILV | 9 | 0.18597 | 1.0723 |
| LSDRIAPRH | 9 | 0.24659 | 1.0661 |
| GQAANLVLA | 9 | 0.06369 | 1.0443 |

Rv1981c CTL epitope

| epitope | length | immunogenicity | antigenicity |
| --- | --- | --- | --- |
| DTAQATVGA | 9 | 0.02542 | 1.3115 |
| DAERADHREY | 10 | 0.27691 | 1.2323 |
| TEQQTTIRV | 9 | 0.06274 | 1.2253 |
| AERADHREY | 9 | 0.2104 | 1.1519 |
| IRDEAVHGY | 9 | 0.23201 | 1.1446 |
| VMLESFLFY | 9 | 0.10529 | 1.1423 |
| SVMLESFLFY | 10 | 0.03748 | 1.0329 |

Rv2659c CTL epitope

| epitope | length | immunogenicity | antigenicity |
| --- | --- | --- | --- |
| RPDLRVHDL | 9 | 0.12556 | 2.4462 |
| DLHGEVARVR | 10 | 0.28693 | 1.806 |
| HSTAGAALR | 9 | 0.14834 | 1.7454 |
| DISIPPHLI | 9 | 0.0798 | 1.6447 |
| SIPPHLIPA | 9 | 0.11214 | 1.6289 |
| FNAKIDAEAW | 10 | 0.09109 | 1.6286 |
| ELRRKDIDL | 9 | 0.00504 | 1.6241 |
| IPPHLIPAI | 9 | 0.15693 | 1.6133 |
| DAGVRDISI | 9 | 0.13948 | 1.5829 |
| HSTAGAALRY | 10 | 0.18078 | 1.5629 |
| STAGAALRY | 9 | 0.14261 | 1.5517 |
| IDLHGEVARV | 10 | 0.26157 | 1.4721 |
| NAKIDAEAW | 9 | 0.22971 | 1.3562 |
| YTGPDGRVY | 9 | 0.12114 | 1.3388 |
| KAAGRPDLR | 9 | 0.099 | 1.3142 |
| SYTGPDGRVY | 10 | 0.1572 | 1.1877 |
| RTRAHYRKL | 9 | 0.00187 | 1.0337 |
| ASYTGPDGR | 9 | 0.09894 | 1.0059 |

Rv2660c CTL epitope

| epitope | length | immunogenicity | antigenicity |
| --- | --- | --- | --- |
| ASGGVTVGV | 9 | 0.17648 | 1.8481 |

Rv3873 CTL epitope

| epitope | length | immunogenicity | antigenicity |
| --- | --- | --- | --- |
| TATHGANVSL | 10 | 0.04834 | 1.8613 |
| ATHGANVSL | 9 | 0.01479 | 1.8216 |
| STRAASARTA | 10 | 0.03636 | 1.5717 |
| VTATHGANV | 9 | 0.1444 | 1.5705 |
| QLADTTDQR | 9 | 0.0604 | 1.4586 |
| LLPPAPVDV | 9 | 0.0607 | 1.4273 |
| QLADTTDQRL | 10 | 0.06398 | 1.3968 |
| TATDAAVQR | 9 | 0.07701 | 1.3947 |
| NASDNNAGDY | 10 | 0.03441 | 1.2801 |
| ASSDPGVAK | 9 | 0.04742 | 1.271 |
| ATGREAAHLR | 10 | 0.25633 | 1.2641 |
| HTATDAAVQR | 10 | 0.11545 | 1.2446 |
| TGREAAHLR | 9 | 0.2133 | 1.1986 |
| ASDNNAGDY | 9 | 0.07278 | 1.1931 |
| DAATAAASAR | 10 | 0.04803 | 1.1863 |
| AASGVPGAR | 9 | 0.06162 | 1.1349 |
| APSTRAASA | 9 | 0.00895 | 1.0843 |
| VADWLYWQY | 9 | 0.33507 | 1.0776 |
| DADERHTAI | 9 | 0.24442 | 1.0676 |
| AVADWLYWQY | 10 | 0.3557 | 1.0645 |
| RQRGRGDAL | 9 | 0.17478 | 1.0645 |
| ATDAAVQRV | 9 | 0.05601 | 1.0449 |
| EAAHLRAFR | 9 | 0.18459 | 1.032 |
