# Supplementary material for: In silico Analysis of Peptide-Based Biomarkers for the Diagnosis and Prevention of Latent Tuberculosis Infection
Source: Front Microbiol. 2022 Jun 28;13:947852. doi: 10.3389/fmicb.2022.947852 (PMC9273951; doi:10.3389/fmicb.2022.947852)
Supplement: Supplementary Figure 1 — The polypeptide molecular sequence was electronically cloned into the pET30a expression vector by SnapGene software. The diagnostic antigen is the gene coding for the insertion of antigen molecules, and the rest is the expression vector. [file Data_Sheet_1.zip › Supplementary Files/Table S4.docx]

Rv1737c B-cell epitope

| Sequence | Start position | Score |
| --- | --- | --- |
| YLPTYITTIYGFSTVDAGAR | 228 | 0.89 |
| DRIAPRHVVLASLAGTALLA | 267 | 0.86 |
| RAMLIAVTLASILPVLAVGV | 71 | 0.82 |
| PVGGWLSDRIAPRHVVLASL | 260 | 0.82 |
| LSAFFTPRFVRWFGLFTTHA | 146 | 0.81 |
| GFVAFSNYLPTYITTIYGFS | 221 | 0.8 |
| LASTAVVAMVVLRDAPYFRP | 171 | 0.8 |
| GGVFAWVARRAPAASVGSVT | 315 | 0.79 |
| DAGARTAGFALAAVLARPVG | 243 | 0.78 |
| NLIGPLSTSYARDMSLSSAE | 23 | 0.78 |
| EVWSAATFITLAVCLGVGTG | 296 | 0.76 |
| AALQPPPEVWSAATFITLAV | 289 | 0.76 |
| PRLKAAARLPVTWEMSFLYA | 197 | 0.76 |
| LTDRFGGRAMLIAVTLASIL | 64 | 0.74 |
| SLAGTALLAFAAALQPPPEV | 278 | 0.74 |
| GVFGMGMVGTALSAFFTPRF | 135 | 0.74 |
| FLYAIVFGGFVAFSNYLPTY | 213 | 0.73 |
| DAPYFRPNADPVLPRLKAAA | 184 | 0.73 |
| SVVNFWAWNLIGPLSTSYAR | 15 | 0.73 |
| GATYDPVDNDYTVGLLLLVA | 353 | 0.71 |
| TGIVAAAGGLGGYFPPLVMG | 334 | 0.71 |
| EASLLVATPILVGALGRIVT | 42 | 0.68 |
| ALGRIVTGPLTDRFGGRAML | 55 | 0.67 |
| DNDYTVGLLLLVATALVACT | 360 | 0.67 |
| PAASVGSVTGIVAAAGGLGG | 326 | 0.64 |
| PFANNWYQPARRGFSTGVFG | 119 | 0.64 |
| VAGTIFAVGIPFANNWYQPA | 109 | 0.63 |
| LVFFGLFLGVAGTIFAVGIP | 100 | 0.55 |

Rv1981c B-cell epitope

| Sequence | Start position | Score |
| --- | --- | --- |
| RGDDALKRKASSVMLESFLF | 146 | 0.95 |
| SNDLASWQTLSSTEQQTTIR | 42 | 0.87 |
| NEIDYAHDLYDELGWTDDVL | 235 | 0.85 |
| YLQRKAQIIVDYYRGDDALK | 133 | 0.85 |
| INWNRLLDAKDLQVWERLTG | 12 | 0.84 |
| RDTCQVNPAVRAALDPGAGE | 276 | 0.82 |
| GNFWLPEKIPLSNDLASWQT | 31 | 0.81 |
| TLCSTKQIDDAFDWSEQNPY | 114 | 0.81 |
| SYSSIFSTLCSTKQIDDAFD | 107 | 0.79 |
| STEQQTTIRVFTGLTLLDTA | 53 | 0.78 |
| QRGLADLTDAERADHREYTC | 207 | 0.77 |
| LPMYWSSRGKLTNTADLIRL | 171 | 0.77 |
| SSVMLESFLFYSGFYLPMYW | 156 | 0.75 |
| YNANKALANLGYQPAFDRDT | 259 | 0.74 |
| LIRLIIRDEAVHGYYIGYKC | 187 | 0.74 |
| DAFDWSEQNPYLQRKAQIIV | 123 | 0.73 |
| FLFYSGFYLPMYWSSRGKLT | 163 | 0.71 |
| GENHDFFSGSGSSYVMGTHQ | 294 | 0.7 |
| PHEEAVLTNMAFMESVHAKS | 88 | 0.69 |
| DTAQATVGAVAMIDDAVTPH | 70 | 0.69 |
| GSGSSYVMGTHQPTTDTDWD | 302 | 0.68 |
| MTGKLVERVHAINWNRLLDA | 1 | 0.67 |
| IRVFTGLTLLDTAQATVGAV | 60 | 0.65 |
| VAMIDDAVTPHEEAVLTNMA | 79 | 0.62 |
| HREYTCELLHTLYANEIDYA | 221 | 0.59 |
| TNMAFMESVHAKSYSSIFST | 95 | 0.58 |
| YDELGWTDDVLPYMRYNANK | 244 | 0.53 |
| DEAVHGYYIGYKCQRGLADL | 194 | 0.51 |

Rv2659c B-cell epitope

| Sequence | Start position | Score |
| --- | --- | --- |
| RRKFGRIRQFNSGRWQASYT | 9 | 0.92 |
| RVHDLRHSGAVLAASTGATL | 314 | 0.88 |
| TRAHYRKLLDNHILATFADT | 94 | 0.86 |
| GELTELRRKDIDLHGEVARV | 211 | 0.86 |
| AWLAMRYGELTELRRKDIDL | 204 | 0.86 |
| ATLAELMQRLGHSTAGAALR | 331 | 0.84 |
| PSALYRMFYKARKAAGRPDL | 294 | 0.84 |
| TGPDGRVYIAPKTFNAKIDA | 28 | 0.84 |
| ASGQEDRPGAPFGEYAEGWL | 66 | 0.81 |
| APKTFNAKIDAEAWLTDRRR | 37 | 0.81 |
| SGAVLAASTGATLAELMQRL | 321 | 0.81 |
| GEYAEGWLKQRGIKDRTRAH | 78 | 0.8 |
| DTDLRDITPAAVRRWYATTA | 112 | 0.8 |
| FPSVNDPNRHLAPSALYRMF | 282 | 0.79 |
| RYQHAAKGRDREIAALLSKL | 350 | 0.78 |
| RKAAGRPDLRVHDLRHSGAV | 305 | 0.77 |
| QFNSGRWQASYTGPDGRVYI | 17 | 0.77 |
| NPCRISGASTARRVHKIRPA | 161 | 0.77 |
| IDLHGEVARVRRAVVRVGEG | 221 | 0.76 |
| YSLLRAIMQTALADDLIDSN | 142 | 0.75 |
| GHSTAGAALRYQHAAKGRDR | 341 | 0.74 |
| PDPYQAFVLMAAWLAMRYGE | 193 | 0.74 |
| LADDLIDSNPCRISGASTAR | 153 | 0.74 |
| LDNHILATFADTDLRDITPA | 102 | 0.74 |
| HVNPGRESLLFPSVNDPNRH | 272 | 0.72 |
| ISIPPHLIPAIEDHLHKHVN | 255 | 0.71 |
| DELETITKAMPDPYQAFVLM | 183 | 0.68 |
| RAVVRVGEGFKVTTPKSDAG | 232 | 0.65 |
| PTMRAHSYSLLRAIMQTALA | 135 | 0.65 |
| PKSDAGVRDISIPPHLIPAI | 246 | 0.64 |
| YATTAVGTPTMRAHSYSLLR | 127 | 0.64 |
| AWLTDRRREIDRQLWSPASG | 49 | 0.6 |
| TARRVHKIRPATLDELETIT | 170 | 0.58 |
| AIEDHLHKHVNPGRESLLFP | 264 | 0.57 |

Rv2660c B-cell epitope

| Sequence | Start position | Score |
| --- | --- | --- |
| GGVTVGVGVGTEQRNLSVVA | 24 | 0.88 |
| SSRSPDFVDETAGQSWCAIL | 50 | 0.73 |
| ASQRAAGASGGVTVGVGVGT | 15 | 0.72 |
| RNLSVVAPSQFTFSSRSPDF | 37 | 0.66 |

Rv3879c B-cell epitope

| Sequence | Start position | Score |
| --- | --- | --- |
| AASVTPAAASGVPGARAAAA | 359 | 0.93 |
| QSGPAHADESAASVTPAAAS | 349 | 0.91 |
| SGPATPGTPGGEPAPHVKPA | 312 | 0.91 |
| GTPGEPTPITPVTPPVAPAT | 254 | 0.91 |
| AAAAPSGTAVGAGARSSVGT | 376 | 0.9 |
| GVPGARAAAAAPSGTAVGAG | 369 | 0.9 |
| PVTPGKPVTPVTPVKPGTPG | 238 | 0.89 |
| TATDAAVQRVAVADWLYWQY | 697 | 0.88 |
| ADHAIPVDEIARCATYPVLA | 547 | 0.88 |
| NALEDLLQQKSPPPPDVPTL | 171 | 0.88 |
| TAAASGAGSHAATGRAPVAT | 395 | 0.87 |
| PGPQPVTPATPGPSGPATPG | 299 | 0.87 |
| PATPATPATPVTPAPAPHPQ | 271 | 0.87 |
| FRAYAAHSQEIALHQAHTAT | 680 | 0.86 |
| LWSGGAANAANGALGANINQ | 61 | 0.86 |
| ASARTAPPARPPSTDHIDKP | 426 | 0.86 |
| GAPVTPITPTPGTPVTPVTP | 222 | 0.86 |
| GTPITPGTPITPGTPITPIP | 202 | 0.86 |
| RSRLEVVDPSAAAQLADTTD | 614 | 0.85 |
| SHAATGRAPVATSDKAAAPS | 403 | 0.85 |
| PHPQPAPAPAPSPGPQPVTP | 287 | 0.84 |
| TPTPGTPVTPVTPGKPVTPV | 229 | 0.84 |
| DAATAAASARQRGRGDALRL | 470 | 0.83 |
| AAAPSTRAASARTAPPARPP | 418 | 0.83 |
| TDQRLLDLLPPAPVDVNPPG | 632 | 0.82 |
| AEQPGVPGQHAGGGTQSGPA | 334 | 0.82 |
| GSYARQMLDPGGWVEADEDT | 8 | 0.81 |
| TAINSLVTATHGANVSLVAE | 136 | 0.81 |
| QREIDILENDPSLDADERHT | 117 | 0.81 |
| ITPVTPPVAPATPATPATPV | 262 | 0.8 |
| TPVTPAPAPHPQPAPAPAPS | 279 | 0.79 |
| PSLDADERHTAINSLVTATH | 127 | 0.79 |
| LMKPMTSTATGREAAHLRAF | 661 | 0.78 |
| IPDGMELPNKVYLASADHAI | 532 | 0.78 |
| HVKPAALAEQPGVPGQHAGG | 327 | 0.78 |
| ANSYGLAYIPDGMELPNKVY | 524 | 0.77 |
| LARRIAAALNASDNNAGDYG | 489 | 0.77 |
| ESKNWKPPKNALEDLLQQKS | 162 | 0.77 |
| GDYGFFWITAVTTDGSIVVA | 505 | 0.76 |
| VTDVLDTCRQQKGHVFEGGL | 42 | 0.76 |
| RAQEYSQVLQRVTDVLDTCR | 31 | 0.76 |
| TLVVPSPGTPGTPGTPITPG | 189 | 0.75 |
| GALGANINQLMTLQDYLATV | 72 | 0.74 |
| QDYLATVITWHRHIAGLIEQ | 85 | 0.73 |
| GHVFEGGLWSGGAANAANGA | 54 | 0.73 |
| TYPVLAVQAWAAFHDMTLRA | 561 | 0.72 |
| PPSTDHIDKPDRSESADDGT | 436 | 0.72 |
| LVAETAERVLESKNWKPPKN | 152 | 0.72 |
| DERHMLWFELMKPMTSTATG | 652 | 0.71 |
| AAFHDMTLRAVIGTAEQLAS | 571 | 0.71 |
| PVDVNPPGDERHMLWFELMK | 644 | 0.7 |
| IVLEPDDIPESGKMTGRSRL | 598 | 0.7 |
| GNNVDGAQREIDILENDPSL | 110 | 0.69 |
| TGREAAHLRAFRAYAAHSQE | 670 | 0.68 |
| VSMIPVSAARAARDAATAAA | 457 | 0.67 |
| SADDGTPVSMIPVSAARAAR | 450 | 0.67 |
| ITWHRHIAGLIEQAKSDIGN | 92 | 0.63 |
| RQRGRGDALRLARRIAAALN | 479 | 0.63 |
| TPITPGTPITPIPGAPVTPI | 209 | 0.62 |
| GTAEQLASSDPGVAKIVLEP | 583 | 0.6 |
