# Supplementary figures and images for: In silico Analysis of Peptide-Based Biomarkers for the Diagnosis and Prevention of Latent Tuberculosis Infection
Source: Front Microbiol. 2022 Jun 28;13:947852. doi: 10.3389/fmicb.2022.947852 (PMC9273951; doi:10.3389/fmicb.2022.947852)

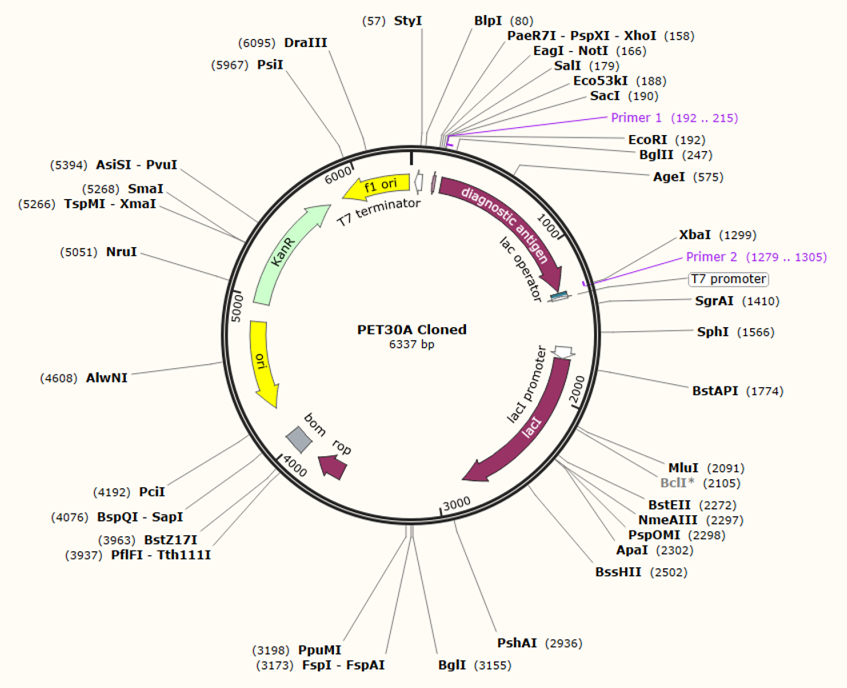

Supplement: Supplementary Figure 1 — The polypeptide molecular sequence was electronically cloned into the pET30a expression vector by SnapGene software. The diagnostic antigen is the gene coding for the insertion of antigen molecules, and the rest is the expression vector. [file Data_Sheet_1.zip › Supplementary Files/Figure S1.TIF]
